# Safety and efficacy of intravenous belimumab in children with systemic lupus erythematosus: results from a randomised, placebo-controlled trial

**DOI:** 10.1136/annrheumdis-2020-217101

**Published:** 2020-07-22

**Authors:** Hermine I Brunner, Carlos Abud-Mendoza, Diego O Viola, Inmaculada Calvo Penades, Deborah Levy, Jordi Anton, Julia E Calderon, Vyacheslav G Chasnyk, Manuel A Ferrandiz, Vladimir Keltsev, Maria E Paz Gastanaga, Michael Shishov, Alina Lucica Boteanu, Michael Henrickson, Damon Bass, Kenneth Clark, Anne Hammer, Beulah N Ji, Antonio Nino, David A Roth, Herbert Struemper, Mei-Lun Wang, Alberto Martini, Daniel Lovell, Nicolino Ruperto, in collaboration with the Paediatric Rheumatology International Trials Organisation (PRINTO) and the Pediatric Rheumatology Collaborative Study Group (PRCSG), Rubén Cuttica

**Affiliations:** 1 Cincinnati Children's Hospital Medical Center, Division of Rheumatology, University of Cincinnati, Cincinnati, Ohio, USA; 2 Hospital Central “Dr Ignacio Morones Prieto”, Unidad Regional de Reumatologia y Osteoporosis, Hospital Central and Facultad de Medicina de la Universidad Autónoma de San Luis Potosí, San Luis Potosí, Mexico; 3 Reumatologia, Instituto CAICI, Rosario, Argentina; 4 Pediatric Rheumatology Unit, Hospital Universitario y Politecnico la Fe, Valencia, Spain; 5 Rheumatology, Hospital for Sick Children and Univeristy of Toronto, Toronto, Ontario, Canada; 6 Division of Pediatric Rheumatology, Hospital Sant Joan de Déu, Universitat de Barcelona, Barcelona, Spain; 7 El Derby, Instituto de Ginecologia y Reproduccion, Lima, Peru; 8 Department of Hospital Pediatrics, Saint Petersburg State Pediatric Medical University, Saint Petersburg, Russian Federation; 9 Reumatologia, Instituto Nacional de Salud del Niño, Lima, Peru; 10 Pediatric Department, Togliatti City Clinical Hospital №5, Togliatti, Russian Federation; 11 Servicio de Reumatologia, Clinica Anglo Americana, Lima, Peru; 12 Phoenix Children's Hospital, Phoenix, Arizona, USA; 13 Pediatric Rheumatology Unit, University Hospital Ramón y Cajal, Madrid, Spain; 14 GSK, Collegevile, Pennsylvania, USA; 15 GSK, Stevenage, UK; 16 GSK, Research Triangle Park, North Carolina, USA; 17 Dipartimento di Neuroscienze, Riabilitazione, Oftalmologia, Genetica e Scienze Materno-Infantili (DiNOGMI), Università degli Studi di Genova, Genova, Liguria, Italy; 18 Clinica Pediatrica e Reumatologia, PRINTO, IRCCS Istituto Giannina Gaslini, Genoa, Italy

**Keywords:** systemic lupus erythematosus, treatment, DMARDs (biologic)

## Abstract

**Objectives:**

This ongoing Phase-2, randomised, placebo-controlled, double-blind study evaluated the efficacy, safety and pharmacokinetics of intravenous belimumab in childhood-onset systemic lupus erythematosus (cSLE).

**Methods:**

Patients (5 to 17 years) were randomised to belimumab 10 mg/kg intravenous or placebo every 4 weeks, plus standard SLE therapy. Primary endpoint: SLE Responder Index (SRI4) response rate (Week 52). Key major secondary endpoints: proportion of patients achieving the Paediatric Rheumatology International Trials Organisation/American College of Rheumatology (PRINTO/ACR) response using 50 and ‘30 alternative’ definitions (Week 52), and sustained response (Weeks 44 to 52) by SRI4 and Parent Global Assessment of well-being (Parent-global). Safety and pharmacokinetics were assessed. Study not powered for statistical testing.

**Results:**

Ninety-three patients were randomised (belimumab, n=53; placebo, n=40). At Week 52, there were numerically more SRI4 responders with belimumab versus placebo (52.8% vs 43.6%; OR 1.49 (95% CI 0.64 to 3.46)). PRINTO/ACR 30 alternative (52.8% vs 27.5%; OR 2.92 (95% CI 1.19 to 7.17)) and PRINTO/ACR 50 (60.4% vs 35.0%; OR 2.74 (95% CI 1.15 to 6.54)) responses were more frequent with belimumab than placebo, as were sustained responses for SRI4 (belimumab, 43.4%; placebo, 41.0%; OR 1.08 (95% CI 0.46 to 2.52)) and Parent-global (belimumab, 59.1%; placebo, 33.3%; OR 3.49 (95% CI 1.23 to 9.91)). Serious adverse events were reported in 17.0% of belimumab patients and 35.0% of placebo patients; one death occurred (placebo). Week-52, geometric mean (95% CI) belimumab trough concentration was 56.2 (45.2 to 69.8) µg/mL.

**Conclusion:**

The belimumab intravenous pharmacokinetics and benefit–risk profile in cSLE are consistent with adult belimumab studies and the 10 mg/kg every 4 weeks dose is appropriate.

**Trial registration number:**

NCT01649765.

**Key messages**

**What is already known about this subject?**

- Paediatric patients with childhood-onset systemic lupus erythematosus (cSLE) have higher disease activity and faster damage accrual over time compared with those diagnosed with SLE in adulthood. Very few drugs have been studied in cSLE.
- Belimumab targets B cell-activating factor.

**What does this study add?**

- Our study (PLUTO) is the first trial of intravenous belimumab in children with active cSLE; we evaluated the efficacy, safety, pharmacokinetics (PK) and pharmacodynamics (PD) of intravenous belimumab 10 mg/kg, plus standard SLE therapy versus placebo.
- At Week 52, compared with placebo, numerically higher proportions of patients receiving belimumab met the primary efficacy endpoint of SLE Responder Index 4 response rate, classically used in adult trials. The major secondary endpoints, including the Paediatric Rheumatology International Trials Organisation/American College of Rheumatology response criteria, also favoured belimumab over placebo. Overall, belimumab was well tolerated by paediatric patients, and the PK, PD and safety profiles were similar to those of adults with SLE. A 10 mg/kg dose administered intravenously on Days 0, 14 and 28, then every 28 days, is appropriate for use in cSLE.

**Key messages**

**How might this impact on clinical practice or future developments?**

- The favourable results of the PLUTO trial, taken in context with the results from belimumab studies in adults, played a fundamental role in the approval of belimumab as add-on therapy in children with cSLE.
- While the results of the double-blind treatment phase are reported herein, the ongoing follow-up phase will provide further evidence regarding long-term (up to 10 years) safety and efficacy of belimumab in children with cSLE.

## Introduction

Systemic lupus erythematosus (SLE) is a relapsing, chronic, inflammatory autoimmune disease with diverse clinical and laboratory manifestations.^1^ Childhood-onset SLE (cSLE) is rare, with estimated annual incidence of 0.3 to 0.9/100 000 children.^2^ Compared with SLE starting in adulthood, there is higher disease activity; increased rates of renal, neurological and haematological involvement; and faster damage accrual over time with cSLE.^2 3^ Paediatric patients are typically treated with combinations of corticosteroids, immunosuppressants, antimalarials and non-steroidal anti-inflammatory drugs, although none are approved.^4^

Patients with SLE have elevated B cell-activating factor (BAFF) levels promoting abnormal B cell activation and differentiation.^5^ Belimumab is a recombinant, immunoglobulin G1λ human monoclonal antibody that antagonises biological activity of soluble BAFF.^6^ Belimumab is the first treatment approved for children with cSLE.^7–9^

Double-blind, placebo-controlled trials are rarely performed in cSLE, making it difficult to determine new treatment benefits over placebo or current standard SLE therapy in this population. This is the first belimumab trial in cSLE and was done to evaluate the efficacy, safety, tolerability, pharmacokinetics (PK) and pharmacodynamics (PD) of intravenous belimumab 10 mg/kg versus placebo, plus standard SLE therapy, in patients with cSLE ages 5 to 17 years without severe lupus nephritis. We report results from the 52 week, double-blind treatment period (Part A) of this ongoing trial, which contributed to belimumab’s recent approval an add-on therapy in children with cSLE.

## Methods

### Study design

This Phase 2, multicentre, randomised, double-blind, placebo-controlled study in paediatric patients with active cSLE (PLUTO Part A; NCT01649765; GSK study BEL114055) consisted of three parts: 52-week double-blind period where patients were randomised to receive either belimumab or placebo (Part A); open-label extension of ≤10 years, where all Part A completers receive belimumab (Part B); and long-term safety follow-up for patients who withdraw anytime from Parts A or B (Part C); Parts B and C are ongoing. For Part A, 29 centres, most from the Paediatric Rheumatology International Trials Organisation (PRINTO) and Pediatric Rheumatology Collaborative Study Group (PRCSG) networks,^10 11^ recruited patients in 10 countries from North, Central and South America, Europe and Japan (9/2012–1/2017; Online supplementary table S1). Patients were discontinued from the study for pregnancy, receiving prohibited therapy, treatment failure, unacceptable toxicity, serious infection, ≥3 consecutive doses of study treatment missed or patient/legal representative decision.

10.1136/annrheumdis-2020-217101.supp1 Supplementary data

This report conforms to the CONsolidated Standards of Reporting Trials (CONSORT) guideline.^12^

### Patient inclusion and exclusion criteria

Eligible patients were ages 5 to 17 years with clinically active SLE disease, defined as Safety of Estrogens in Lupus Erythematosus National Assessment-SLE Disease Activity Index (SELENA-SLEDAI) score ≥6 at screening,^13^ fulfilled ≥4 of 11 American College of Rheumatology (ACR) criteria for classification of SLE^14^ and had an unequivocally double positive test result for antinuclear antibody ≥1:80 and/or anti-double-stranded (ds)DNA ≥30 IU/mL antibody, either from two independent time points within the screening period or one positive historical test result and one positive test result during the screening period. Main exclusionary criteria were: active central nervous system SLE or acute severe lupus nephritis (LN), or systemic prednisone (or equivalent) >1.5 mg/kg/day, B cell-targeted therapy within 1 year or prior belimumab use (see online supplementary protocol for complete eligibility criteria).

### Randomisation and masking

Patients were assigned a unique patient number at screening and were randomised centrally using an interactive response system. Based on age and enrolment sequence, patients were randomised to one of the three cohorts and received belimumab 10 mg/kg intravenous or placebo on Days 0, 14 and 28, then every 28 days until Week 48, with a final evaluation at Week 52 (online supplementary figure S1). Enrolment commenced with patients ages 12 to 17 years (Cohort 1, n=12; belimumab, n=10; placebo, n=2) followed by those ages 5 to 11 years (Cohort 2, n=13; belimumab, n=10; placebo, n=3); Cohort 3 included 68 patients (belimumab, n=33; placebo n=35) ages 12 to 17 years. On confirmation of belimumab dosing that resulted in belimumab exposure similar to adults in PK analyses of Cohort 1, enrolment to Cohorts 2 and 3 occurred. Cohort 3 was initially designed to have patient ages of 5 to 17 years; however, the overall study enrolment target was achieved before the PK analyses for Cohort 2 were completed. Randomisation in Cohort 3 was stratified by age (5 to 11 vs 12 to 17 years) and screening SELENA-SLEDAI scores (6 to 12 vs ≥13) (online supplementary figure S1). Belimumab-to-placebo ratio was 5:1 (Cohorts 1 and 2) and 1:1 (Cohort 3). Patients continued to receive standard SLE therapy, including immunosuppressants or corticosteroids, with progressive restrictions on permitted medication changes, but no forced taper (online supplementary figure S2). Except for a pharmacist who prepared the intravenous injections, all study site personnel, patients and the sponsor’s study team remained blinded to the study agent (belimumab or placebo) received; blinded treatment was administered over a minimum of 1 hour.

### Endpoints

The primary endpoint was SLE Responder Index 4 (SRI4) response rate at Week 52, defined as ≥4-point reduction from baseline in SELENA-SLEDAI score, no worsening in Physician’s Global Assessment of cSLE activity (PGA), that is, PGA increase <0.30 points from baseline, no new British Isles Lupus Assessment Group (BILAG) A organ domain score; and no two new BILAG B organ domain scores compared with baseline.^15 16^ Major secondary endpoints at Week 52 included: proportion of patients responding to therapy defined by PRINTO/ACR cSLE criteria,^17–19^ which consider percentage changes from baseline of the five multidimensional core components (PGA (scale 0 to 3), Parent Global Assessment of patient overall well-being (Parent-global, scale 0 to 10), SELENA-SLEDAI, Paediatric Quality of Life inventory (PedsQL; physical-functioning domain, scale 0 to 100) and proteinuria. Improvement in PRINTO/ACR 30 alternative definition is the proportion of patients with ≥30% improvement in three of five cSLE core response criteria and with ≤1 of the remaining worsening by >30%, and in PRINTO/ACR 50 as the proportion of patients with ≥50% improvement in any two of five cSLE core response criteria and ≤1 of the remaining worsening by >30%.^19^ Major secondary endpoints also included the proportion of patients with sustained response in SRI4 and Parent-global, defined as a response at Weeks 44, 48 and 52 (response in Parent-global: improvement of >0.7 (minimally clinically important difference)).

Other efficacy endpoints at Week 52 included: components of SRI4, SRI6 response rate (identical to SRI4, except for higher threshold of improvement for SELENA-SLEDAI ≥6), time to first severe flare (measured using the SLE flare index, modified to exclude the single criterion of increased SELENA–SLEDAI score to >12),^13 20^ mean change from baseline in average daily corticosteroid dose and the proportion of patients with average corticosteroid dose reduction ≥25% from baseline to Weeks 44 to 52, percentage of patients with organ improvement by BILAG at Week 52 among patients with grade A or B domain score at baseline, percentage of patients with organ worsening by BILAG at Week 52 among patients without grade A domain score at baseline, percentage of patients with organ improvement by SELENA SLEDAI at Week 52 among patients with organ system involvement at baseline, percentage of patients with organ worsening by SELENA-SLEDAI at Week 52 among patients without organ system involvement at baseline. Renal endpoints included: proportion of patients with renal flare over 52 weeks among those with high proteinuria (>0.5 mg/mg) at baseline, and proteinuria shifts from high (>0.5 mg/mg) to normal (≤0.5 mg/mg) over 52 weeks. Subgroup analysis of SRI4 response at Week 52 by baseline age is also reported. Efficacy endpoints are further summarised in online supplementary table S2.

Safety was assessed by treatment-emergent adverse events (AEs), serious AEs (SAEs), AEs of special interest (AESI) including malignancies, infusion/anaphylaxis/hypersensitivity reactions, all infections of special interest, depression/suicide/self-injury and deaths. Immunogenicity was measured. PK endpoints included observed belimumab concentrations (geometric means) at Weeks 24 and 52. PD endpoints included B cell subsets, immunoglobulins and SLE biomarkers (anti-dsDNA antibodies, complement C3/C4) at Week 52.

### Statistical analyses

A statistically powered double-blinded study with an appropriate sample size was not deemed feasible. Instead, the study was designed to descriptively evaluate efficacy and safety of belimumab in cSLE, without planned/formal statistical hypothesis testing; no p values are presented. Unless otherwise stated, analyses included the intention-to-treat population, that is, all patients randomised and treated with ≥1 dose of study agent. The analysis considered patients who withdrew prior to Week 52 or those deemed treatment failures (using prohibited medication/non-allowed dose of permitted medication (online supplementary figure S2)) as non-responders. For the primary efficacy analysis and all endpoints with modelling, we used logistic regression to estimate the odds (OR with 95% CIs) of response for belimumab versus placebo (online supplementary table S3). For major secondary efficacy analyses, we used analysis of covariance where appropriate (online supplementary table S3). We used descriptive statistics to summarise continuous and categorical variables. AEs were categorised by system organ class and coded using Medical Dictionary for Regulatory Activities V.20.1. Extrapolation of the primary efficacy endpoint (SRI4 response rate at Week 52) in adults to the paediatric population was performed using a Bayesian statistical approach^21^ and data from two Phase 3 trials of belimumab 10 mg/kg intravenous.^22 23^

### Patient and public involvement

Patients and/or the public were not involved in the design, or conduct, or reporting, or dissemination plans of our research. Patients’ parent/legal guardians were invited to complete the Parent Global Assessment form regarding the patient’s overall well-being.

## Results

### Study population and patient demographics

**Figure 1:**
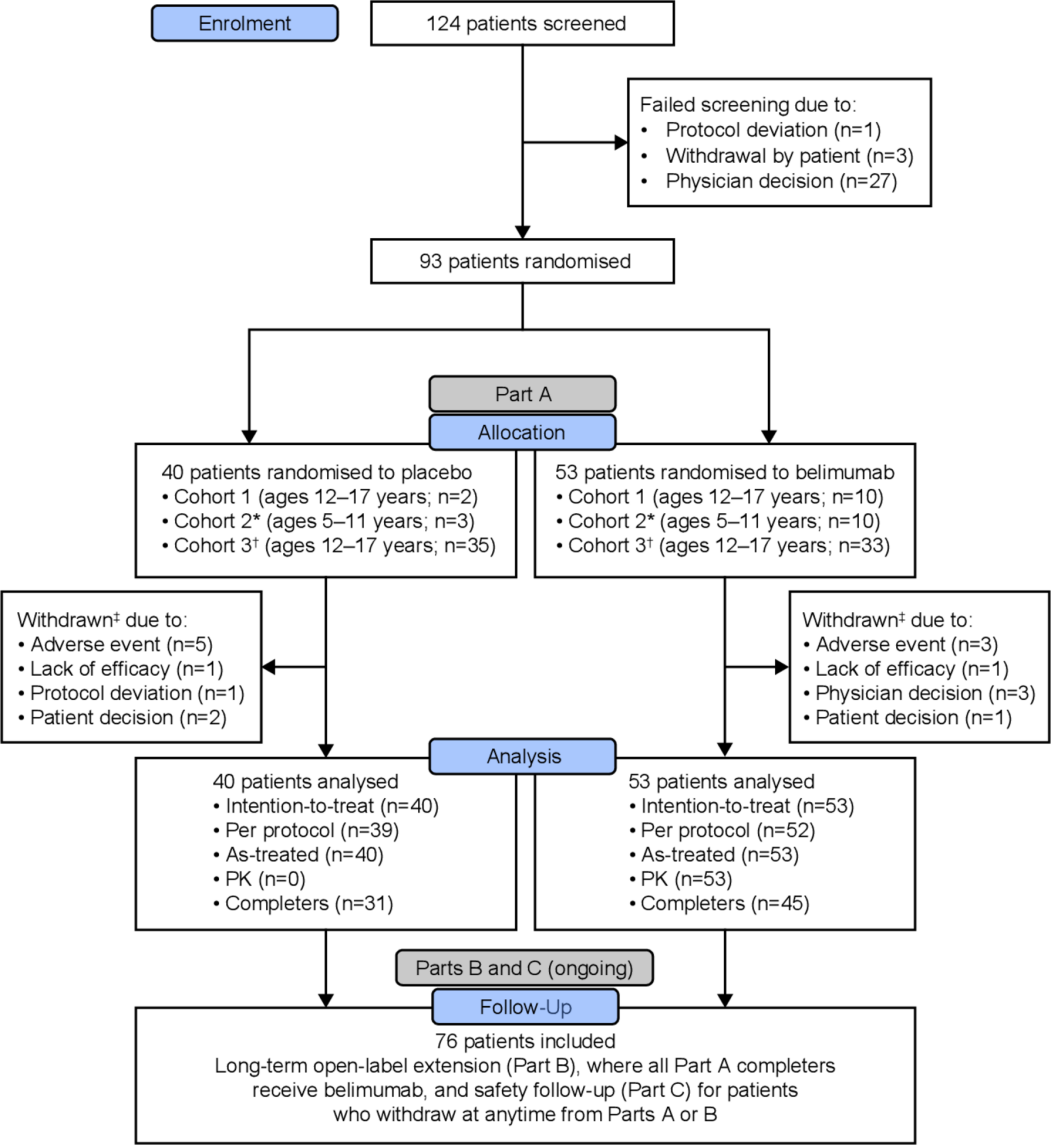
CONSORT diagram of patient disposition. *Initiate Cohort 2 after confirmed/adjusted dose from Cohort 1 PK review; ^†^Cohort 3 was designed to have patient ages of 5 to 17 years, however overall study enrolment target was achieved before Cohort 2 PK analyses completion; ^‡^Patients withdrawn from the study prior to Week 52 are considered treatment failures. CONSORT, CONsolidated Standards of Reporting Trials; PK, pharmacokinetics.

Of 93 randomised patients (belimumab, n=53; placebo, n=40), 76 (81.7%) completed the study through Week 52 (figure 1). The majority were female (88/93 (94.6%)) and 13 were 5 to 11 years (table 1). Baseline characteristics were similar between treatment groups, except for median (IQR) corticosteroid dose (prednisone-equivalent), which was lower in the belimumab group versus placebo (7.50 (5.00 to 10.00) vs 10.0 (7.50 to 16.25) mg/day). Baseline SELENA-SLEDAI organ involvement, BILAG organ domains and ACR classification criteria are presented in online supplementary table S4-S6, respectively.

**Table 1:** Patient demographics and clinical characteristics at baseline

	Placebo (n=40)	Belimumab 10 mg/kg intravenous (n=53)
Age 5–11 years, n (%)	3 (7.5)	10 (18.9)
Age 12–17 years, n (%)	37 (92.5)	43 (81.1)
Female, n (%)	39 (97.5)	49 (92.5)
Weight, kg, median (IQR)	53.30 (47.1 to 60.0)	52.30 (38.7 to 67.0)
Age, years, median (IQR)	15.0 (14.00 to 16.00)	14.0 (12.00 to 15.00)
Disease duration, years (median, IQR)	1.97 (1.30 to 3.57)	1.48 (0.79 to 2.46)
SELENA-SLEDAI score, median (IQR)	10.0 (8.00 to 12.00)	10.0 (8.00 to 12.00)
SELENA-SLEDAI score, n (%)		
≤12	33 (84.6)	43 (81.1)
≥13	6 (15.4)	10 (18.9)
BILAG 1A or 2B domain score at baseline, n (%)	29 (72.5)	37 (69.8)
Physician’s Global Assessment of cSLE activity (PGA)*, median (IQR)	1.3 (1.07 to 1.73)	1.4 (1.05 to 1.50)
Parent-global of patient overall well-being†, median (IQR)	5.0 (3.00 to 6.50)	4.5 (2.50 to 6.50)
Proteinuria‡, mg/mg, median (IQR)	0.12 (0.07 to 0.29)	0.13 (0.08 to 0.21)
Anti-dsDNA antibody positive (≥30 IU/mL), n (%)	27 (67.5)	38 (71.7)
Low complement C3 (<90 mg/dL), n (%)	12 (30.0)	20 (37.7)
Low complement C4 (<10 mg/dL), n (%)	15 (37.5)	21 (39.6)
Anti-dsDNA antibody positive and low complement C3 or C4, n (%)	17 (42.5)	22 (41.5)
PedsQL physical functioning domain score§, median (IQR)	64.1 (45.31 to 79.69)	59.4 (43.75 to 78.13)
Medication usage; n (%)		
Any systemic corticosteroid¶	38 (95.0)	50 (94.3)
Corticosteroid dose, mg/day, median (IQR)**	10.00 (7.50 to 16.25)	7.50 (5.00 to 10.00)
Any immunosuppressant††	27 (67.5)	33 (62.3)
Antimalarial‡‡	31 (77.5)	44 (83.0)
NSAID§§	12 (30.0)	11 (20.8)

*Scale 0 to 3, a lower score indicates better patient well-being.

†Scale 0 to 10, a lower score indicates better patient well-being.

‡Estimated by urinary protein/creatinine ratio.

§Scale 0 to 100.

¶Corticosteroids (prednisone-equivalent) included: deflazacort, meprednisone, methylprednisolone, prednisolone, prednisone, prednisone acetate.

**Calculated for 40 placebo and 53 belimumab patients; 0.00 was imputed for those not receiving corticosteroids at baseline.

††Immunosuppressants included: azathioprine, leflunomide, methotrexate, mycophenolate mofetil, mycophenolate sodium, mycophenolic acid, tacrolimus.

‡‡Antimalarials included: chloroquine, hydroxychloroquine and hydroxychloroquine sulphate.

§§NSAIDs included: diclofenac, ibuprofen, loxoprofen sodium, meloxicam, naproxen, nimesulide.

BILAG, British Isles Lupus Assessment Group; cSLE, childhood-onset systemic lupus erythematosus; dsDNA, double-stranded DNA; NSAID, non-steroidal anti-inflammatory drug; Parent-global, Parent Global Assessment of patient overall well-being; PedsQL, Paediatric Quality of Life inventory generic core scale; PGA, Physician’s Global Assessment of cSLE activity; SELENA-SLEDAI, Safety of Estrogens in Lupus Erythematosus National Assessment-SLE Disease Activity Index.

### Primary endpoint

**Figure 2:**
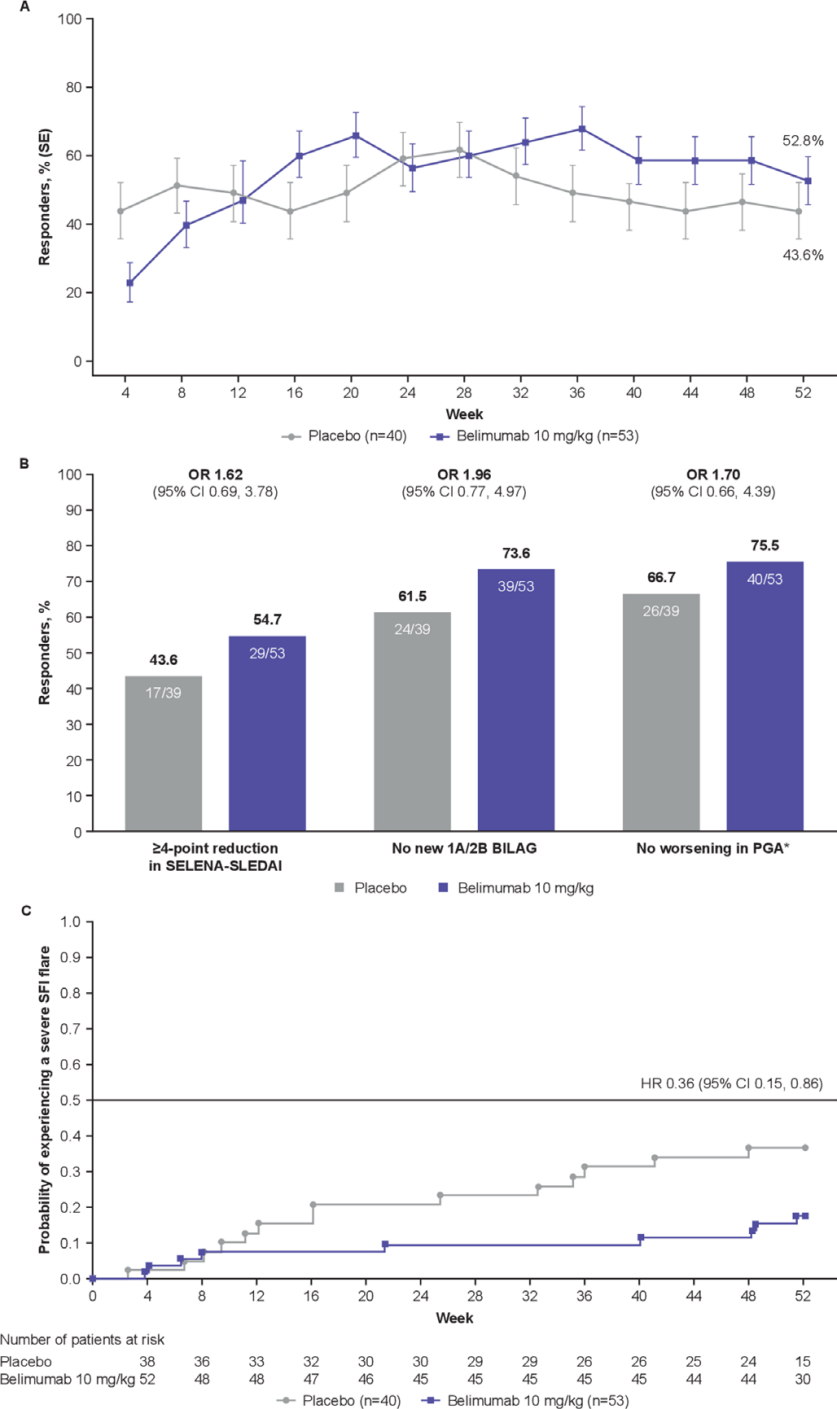
SRI4 response and severe flares. (A) SRI4 response by visit and at Week 52 (ITT population, n=93 for all time points). (B) Components of the SRI4 response at Week 52. (C) Severe flares over 52 weeks. *Defined as increase of<0.30 points from baseline. BILAG, British Isles Lupus Assessment Group; cSLE, childhood-onset systemic lupus erythematosus; ITT, intent-to-treat; PGA, Physician’s Global Assessment of cSLE activity; SELENA-SLEDAI, Safety of Estrogens in Lupus Erythematosus National Assessment-SLE Disease Activity Index; SRI4, SLE Responder Index 4.

More belimumab patients were SRI4 responders compared with placebo (n=28 (52.8%) vs n=17 (43.6%); OR 1.49 (95% CI 0.64 to 3.46)) (figure 2A).

### Major secondary efficacy endpoints

**Figure 3:**
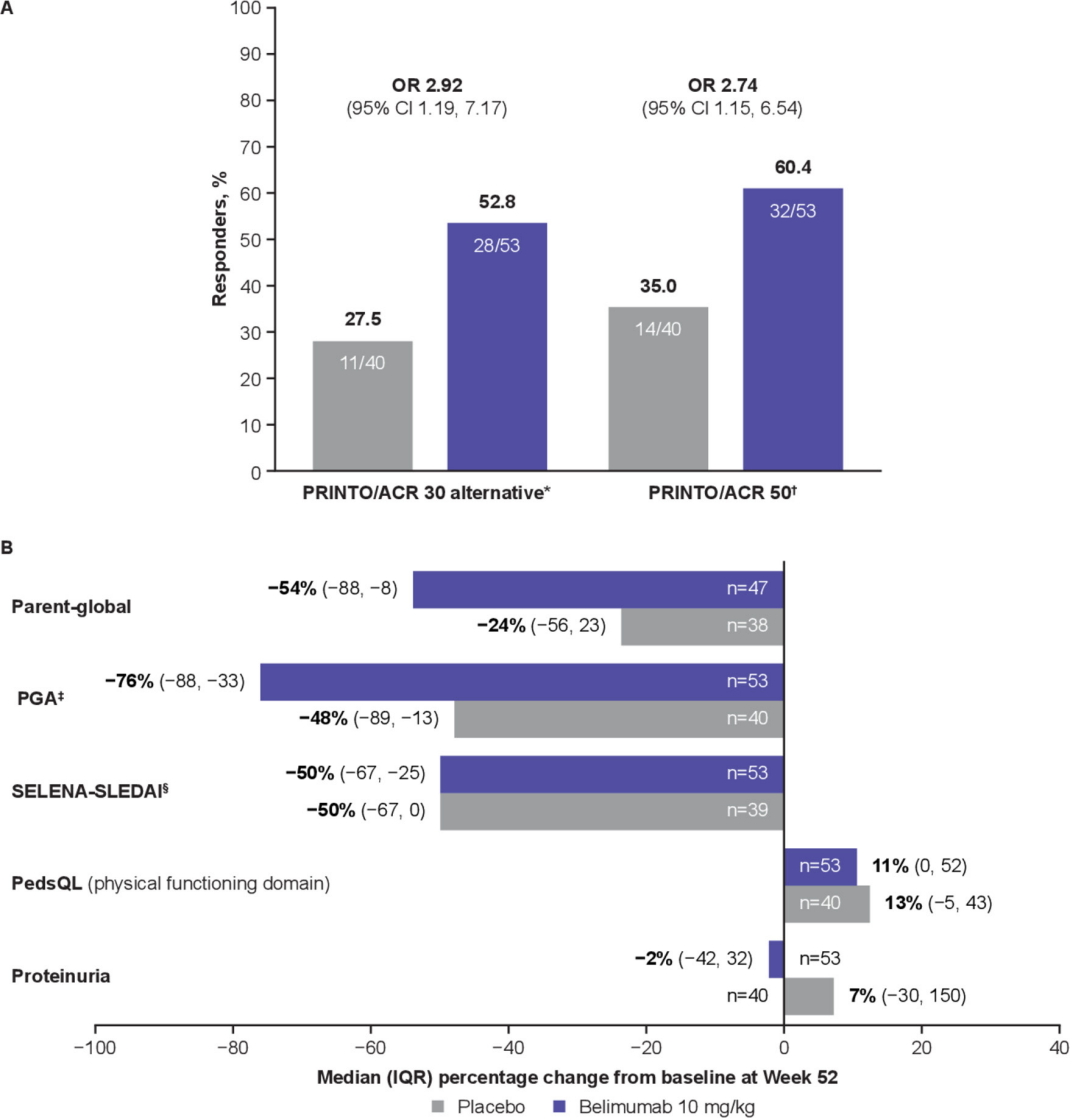
PRINTO/ACR at Week 52. (A) PRINTO/ACR responders at Week 52 by two definitions. (B) Percentage change from baseline in PRINTO/ACR cSLE core components at Week 52. *Defined as the proportion of patients with at least 30% improvement in three of five cSLE core components and no more than one of the remaining worsening more than 30%; ^†^defined as proportion of patients with at least 50% improvement in any two of five cSLE core components and no more than one of the remaining worsening by more than 30%; ^‡^mean (SD) change from baseline in PGA was –48.8 (42.04) for placebo and –56.5 (43.79) for belimumab; ^§^mean (SD) change from baseline in SELENA-SLEDAI was –38.0 (39.50) for placebo and –43.3 (43.73) for belimumab. cSLE, childhood-onset systemic lupus erythematosus; Parent-global, Parent Global Assessment of patient overall well-being; PedsQL, Paediatric Quality of Life inventory generic core scale; PGA, Physician’s Global Assessment of cSLE activity; PRINTO/ACR, Paediatric Rheumatology International Trials Organisation/American College of Rheumatology; SELENA-SLEDAI, Safety of Estrogens in Lupus Erythematosus National Assessment-SLE Disease Activity Index.

Both PRINTO/ACR 30 alternative definition and PRINTO/ACR 50 responses favoured belimumab over placebo (figure 3A). There was a numerically higher percentage improvement at Week 52 from baseline for Parent-global, PGA and proteinuria in the belimumab group versus placebo (figure 3B). Sustained (Weeks 44 to 52) improvement of Parent-global was present in 26/44 (59.1%) patients receiving belimumab and 12/36 (33.3%) placebo patients (OR 3.49 (95% CI 1.23 to 9.91)). SRI4 response was sustained in 23/53 (43.4%) belimumab patients and 16/39 (41.0%) placebo patients during Weeks 44 to 52 (OR 1.08 (95% CI 0.46 to 2.52)).

### Other endpoints

Consistent with the primary analyses, all SRI4 components showed a numerically higher response rate for belimumab versus placebo (figure 2B). At Week 52, 21/51 (41.2%) belimumab and 13/38 (34.2%) placebo patients were SRI6 responders (OR 1.35 (95% CI 0.56 to 3.22)). Over 52 weeks, 9/53 (17.0%) belimumab patients and 14/40 (35.0%) placebo patients experienced severe flare; thus, there was a 64% lower risk of severe flare with belimumab versus placebo (HR 0.36 (95% CI 0.15 to 0.86)) (figure 2C). Median (IQR) time to first severe flare was 150.0 (45.0 to 338.0) days with belimumab and 113.0 (66.0 to 246.0) days with placebo. SRI4 response by baseline age was consistent with the primary analysis (online supplementary figure S3).

At baseline, 50/53 (94.3%) belimumab patients and 38/40 (95.0%) placebo patients were receiving corticosteroids (table 1). At Week 52, median (IQR) change in corticosteroid dose from baseline for belimumab (n=53) and placebo (n=40) groups was 0.0 (–5.00 to 0.00) mg/day and 0.0 (–1.50 to 0.00) mg/day, respectively. Between Weeks 44 and 52, 10/50 (20.0%) belimumab and 8/38 (21.1%) placebo patients had ≥25% reduction in average corticosteroid dose from baseline (OR 0.92 (95% CI 0.29 to 2.88)).

Bayesian analysis of the SRI4 endpoint suggested superiority of belimumab over placebo in children with ≥97.5% probability (per reasonable assumption of ≥55.0% probability a priori that child efficacy is similar to adults; online supplementary Bayesian analysis report).^24^

10.1136/annrheumdis-2020-217101.supp2 Supplementary data

At baseline, >50% of patients reported BILAG grade A/B musculoskeletal (belimumab, 33/53 (62.3%); placebo, 31/40 (77.5%)) and mucocutaneous (belimumab, 43/53 (81.1%); placebo, 27/40 (67.5%)) organ domain involvement (online supplementary table S5). By Week 52, 24/33 (72.7%) belimumab-treated patients versus 18/31 (58.1%) placebo-treated patients had musculoskeletal BILAG organ domain improvements. No observable difference was found in mucocutaneous BILAG organ domain improvements at Week 52 (belimumab, 22/43 (51.2%); placebo, 13/27 (48.1%)).

### Renal endpoints

At baseline, 10/53 (18.9%) belimumab patients had renal SELENA-SLEDAI organ system involvement, of whom 4 (40.0%) reported an improvement at Week 52, compared with 8/40 (20.0%) placebo patients, of whom 1 (12.5%) reported an improvement at Week 52 (treatment difference vs placebo: 27.50%). In patients with no SELENA-SLEDAI renal involvement at baseline (belimumab, 43/53 (81.1%); placebo, 32/40 (80.0%)), 4/43 (9.3%) belimumab patients and 4/32 (12.5%) placebo patients developed new renal involvement by Week 52. In patients who had no BILAG grade A renal involvement at baseline (belimumab, 52/53 (98.1%); placebo, 39/40 (97.5%)), 7/52 (13.5%) belimumab patients and 6/39 (15.4%) placebo patients developed new BILAG grade A renal involvement by Week 52.

The numbers of patients with higher levels of proteinuria (>0.5 mg/mg; maximum reported value: 1.43 mg/mg for belimumab and 6.13 mg/mg for placebo patients) at baseline were 4/53 (7.5%; median (IQR) 0.8 (0.78 to 1.12) mg/mg) belimumab and 9/40 (22.5%; median (IQR) 1.5 (0.88 to 2.35) mg/mg) placebo patients. No patients with higher proteinuria levels at baseline from either treatment group shifted to normal levels (≤0.5 mg/mg) during the course of the study. Over 52 weeks, no belimumab patients experienced a post-baseline renal flare, compared with four placebo patients, all of whom had higher baseline proteinuria.

### Safety

AE incidence was similar between treatment groups with 42/53 (79.2%) belimumab and 33/40 (82.5%) placebo patients reporting ≥1 AE. There were 9/53 (17.0%) belimumab and 14/40 (35.0%) placebo patients who experienced ≥1 SAE. Table 2 summarises SAEs by preferred term occurring in >1 patient in either group. They were LN (belimumab, 2/53; placebo, 2/40) and headache (belimumab, 0/53; placebo, 2/40). Post-infusion systemic reactions were similarly frequent in both treatment groups. Suicidal ideation or behaviour AESIs occurred in 3/40 placebo patients but in 0/53 belimumab patients. No completed suicides occurred. Three out of 53 (5.7%) belimumab patients had AEs leading to discontinuation (one each of LN, hypertransaminasemia and postherpetic neuralgia); there were 5/40 (12.5%) placebo patients with AEs leading to discontinuation (two LN cases and one each of hepatitis A, acute pancreatitis and retinal vasculitis). One death (acute pancreatitis) (1/40 (2.5%)) occurred in the placebo group (table 2). None of the patients developed anti-belimumab antibodies.

**Table 2:** Summary of AEs reported during the study

N (%)*	Placebo (n=40)	Belimumab 10 mg/kg intravenous (n=53)
AEs by system organ class, any†	33 (82.5)	42 (79.2)
Infections and infestations	28 (70.0)	30 (56.6)
Gastrointestinal disorders	16 (40.0)	18 (34.0)
Musculoskeletal and connective tissue disorders	13 (32.5)	11 (20.8)
Nervous system disorders	11 (27.5)	12 (22.6)
Skin and subcutaneous tissue disorders	9 (22.5)	10 (18.9)
General disorders and administration site conditions	9 (22.5)	9 (17.0)
SAEs by system organ class and preferred term, any‡	14 (35.0)	9 (17.0)
Infections and infestations	5 (12.5)	4 (7.5)
Herpes zoster	1 (2.5)	1 (1.9)
Abscess limb	0	1 (1.9)
Epiglottitis	1 (2.5)	0
Gastroenteritis	0	1 (1.9)
Hepatitis A	1 (2.5)	0
Influenza	1 (2.5)	0
Pneumonia	1 (2.5)	0
Vulvar abscess	0	1 (1.9)
Renal and urinary disorders	3 (7.5)	2 (3.8)
Lupus nephritis	2 (5.0)	2 (3.8)
Glomerulonephritis	1 (2.5)	0
Psychiatric disorders§	3 (7.5)	0
Major depression	1 (2.5)	0
Suicidal ideation	1 (2.5)	0
Suicide attempt	1 (2.5)	0
Deaths¶	1 (2.5)	0
AEs of special interest		
All malignancies	0	0
All post-infusion systemic reactions	3 (7.5)	4 (7.5)
Serious post-infusion systemic reactions	0	0
Systemic reactions hypersensitivity	0	0
All infections of special interest**	3 (7.5)	7 (13.2)
Serious infections of special interest	1 (2.5)	1 (1.9)
Depression/suicide††/self-injury	4 (10)	1 (1.9)
Depression	2 (5)	1 (1.9)
Serious depression	1 (2.5)	0
Suicide††/self-injury	3 (7.5)	0
Serious suicide/self-injury	2 (5)	0

†*Number of patients experiencing an event (a patient can experience ≥1 event); ^†^AEs by system organ class that occurred in >20% of patients in either treatment group are listed;

‡SAEs by system organ class that occurred in >5% of patients in either treatment group are listed;

§Psychiatric disorders included: anger, anxiety, initial insomnia, insomnia, major depression, panic reaction, suicidal ideation, suicide attempt and trichotillomania;

¶Acute pancreatitis; considered unrelated to the study agent;

**Infections of special interest included: candida infection, herpes zoster and pulmonary tuberculosis;

††Includes suicide ideation and behaviour.

AE, adverse event; SAE, serious AE.

### Pharmacokinetics

All 53 belimumab patients contributed samples for PK evaluation. Belimumab concentrations reached steady-state levels early in the trial. Levels were maintained throughout Part A in all three cohorts (online supplementary figure S4). Post-infusion (maximum plasma concentration (C_max_)) geometric mean (95% CI) belimumab serum concentration at Week 24 was 325 (288 to 367) µg/mL for overall PK population, 289 (234 to 356) µg/mL for patients ages 5 to 11 years and 334 (290 to 386) µg/mL for patients ages 12 to 17 years. At Week 52, pre-infusion (minimum plasma concentration (C_min_)) belimumab concentration was 56.2 (45.2 to 69.8) µg/mL for overall PK population, 45.0 (27.5 to 73.4) µg/mL for patients ages 5 to 11 years and 59.7 (46.4 to 76.8) µg/mL for patients ages 12 to 17 years.

### Pharmacodynamics and SLE biomarkers

At Week 52, total B cells, naïve B cells, IgG and anti-dsDNA antibodies decreased and complement C3 and C4 increased in belimumab patients versus placebo (online supplementary table S7). Circulating memory B cells approximately doubled by Week 4 and decreased by Week 52 toward their baseline value in belimumab patients (data not shown).

## Discussion

Belimumab is approved for the treatment of adults with active SLE and was recently approved in children over 5 years of age with cSLE. This is the first double-blind, placebo-controlled trial in children with active cSLE to evaluate efficacy, safety and PK of belimumab 10 mg/kg intravenous. At Week 52, compared with placebo, numerically higher proportions of patients receiving belimumab met the primary efficacy endpoint of SRI4, a tool developed for adults with SLE and validated to assess improvement in cSLE.^15^ Response to belimumab, compared with placebo, was consistent with the results of the Phase 3 programme of belimumab in adults with SLE,^22 23 25 26^ as was the lower risk of severe flares. Analyses also considered baseline disease activity status and sought to provide a comparison to belimumab responses in studies of adults.^27^ Belimumab was well tolerated; the safety profile was consistent with observations in adults with SLE, with no new safety concerns identified. Favourable results of PLUTO contributed to belimumab’s approval in several countries as an add-on therapy in children with cSLE.^7–9^

Given the severity and relatively low cSLE prevalence, a double-blinded, placebo-controlled study with a large sample size powered for statistical significance testing was deemed unfeasible. Hence, this study was designed to descriptively evaluate efficacy and safety of belimumab in cSLE. Design and endpoints of this paediatric study were similar to previous adult Phase 3 belimumab intravenous studies, as was the 10 mg/kg dose, which was informed by the results of these studies.^22 23 25^

In this study, patients ages 5 to 11 years had slightly lower belimumab exposures compared with patients ages ≥12 years, likely due to lower average body mass index in younger patients, consistent with body size dependencies observed in adult SLE studies.^28^ Nonetheless, overall PK population post-infusion and pre-infusion belimumab plasma concentrations (C_max_, 325 µg/mL; C_min_, 56.2 µg/mL) were consistent with adult studies (C_max_, 313 µg/mL; C_min_, 55.6 µg/mL). Belimumab exposures in cSLE across age strata were similar to those of adults with SLE, supporting weight-proportional dosing at 10 mg/kg is appropriate for patients with cSLE ages 5 to 17 years from a PK perspective.

Similar to biologic-treatment trials in juvenile idiopathic arthritis,^29–31^ this study’s strength is using multidimensional response criteria specifically developed for paediatric populations.^17–19^

In order to facilitate the comparison between this paediatric study and previous adult belimumab trials in SLE, the SRI tool was selected as the primary outcome measure. However, differences exist in the variation in frequency and severity of disease activity and associated damage observed between child, adolescent and adult patients with SLE.^17–19^ For this reason, the PRINTO/ACR criteria previously validated for use with cSLE^15^ was also used to provide further analyses on patient responses to treatment. Components of the PRINTO/ACR criteria consider relative changes in disease activity through a multidimensional perspective including a physician’s evaluation, disease activity level, 24 hours proteinuria, and parent-reported and patient-reported outcomes measuring quality of life and a child’s overall well-being. Two definitions were analysed as major secondary efficacy endpoints, and both showed more improvement in belimumab versus placebo patients at Week 52 compared with baseline.

Median corticosteroid doses did not decrease by Week 52 in either of the treatment groups. However, corticosteroid dosing was regulated by the study protocol; hence, any changes should be interpreted with caution. Furthermore, the study was neither designed nor powered to interpret corticosteroid-sparing effect, and a forced steroid tapering schedule was not mandated by the protocol.

Due to ongoing studies examining efficacy and safety of belimumab in adult patients with lupus nephritis (NCT01639339), effects on renal outcomes were also investigated; however, due to small group sizes results should be interpreted with caution.

The safety profile of belimumab 10 mg/kg intravenous in cSLE was similar to placebo and consistent with the known safety profile of belimumab intravenous in adults.^7^ There were no imbalances in post-infusion systemic reactions between belimumab and placebo during this study. Notably, suicidal ideation/suicidal behaviour were not reported by patients with cSLE receiving belimumab, nor were there completed suicides. A longer-term assessment of belimumab safety in children with cSLE will be carried out throughout Parts B/C of PLUTO. The PD of belimumab in cSLE is also in line with what has been observed in adult SLE, with changes in major B cell subsets, IgG, anti-dsDNA and complement consistent with belimumab’s mechanism of action and results in adults with SLE.^32^ Overall, safety and efficacy results from this study in paediatric and adolescent patients with cSLE expand the understanding of how belimumab’s clinical effects translate from adult SLE to cSLE. Moreover, PK and PD responses confirmed a similar belimumab pharmacology and mechanism of action in paediatric patients compared with adults.

A limitation of this study is the lack of a sufficient sample size to detect a statistically significant difference in the primary outcome, SRI4, which was based on measures developed for adults with SLE.

In conclusion, within the limits of data analysed to date (1 year), the benefit–risk profile of belimumab 10 mg/kg intravenous in children with cSLE appears favourable and consistent with adults, confirming 10 mg/kg intravenous is appropriate for paediatric populations.
