# Supplementary material for: Safety and efficacy of intravenous belimumab in children with systemic lupus erythematosus: results from a randomised, placebo-controlled trial
Source: Ann Rheum Dis. 2020 Jul 22;79(10):1340–8. doi: 10.1136/annrheumdis-2020-217101 (PMC7509523; doi:10.1136/annrheumdis-2020-217101)
Supplement: Supplementary data [file annrheumdis-2020-217101supp001.pdf]

Supplementary File

The safety and efficacy of intravenous belimumab in children with systemic lupus erythematosus: Results from a randomised, placebo-controlled trial

Hermine I Brunner, Carlos Abud-Mendoza, Diego O Viola, Inmaculada Calvo Penades, Deborah M Levy, Jordi Anton, Julia E Calderon, Vyacheslav G Chasnyk, Manuel A Ferrandiz, Vladimir A Keltsev, Maria E Paz Gastanaga, Michael Shishov, Alina Lucica Boteanu, Michael Henrickson, Damon Bass, Kenneth Clark, Anne Hammer, Beulah Ji, Antonio Nino, David A Roth, Herbert Struemper, Mei-Lun Wang, Alberto Martini, Daniel J Lovell, Nicolino Ruperto in collaboration with the Paediatric Rheumatology International Trials Organisation (PRINTO) and the Pediatric Rheumatology Collaborative Study Group (PRCSG)

Brunner HI, *et al. Ann Rheum Dis* 2020; 79:1340–1348. doi: 10.1136/annrheumdis-2020-217101

Supplementary File

Table S1

Number of patients randomised by country and research site

**Country**	**Placebo (N=40)**<br>n (%)	**Belimumab 10 mg/kg IV (N=53)**<br>n (%)
**Argentina**		
All sites	5 (12.5)	7 (13.2)
Site 1	1 (2.5)	0
Site 2	4 (10.0)	7 (13.2)
**Canada**		
All sites	2 (5.0)	3 (5.7)
Site 1	2 (5.0)	2 (3.8)
Site 2	0	1 (1.9)*
**Japan**		
All sites	4 (10.0)	2 (3.8)
Site 1	1 (2.5)	1 (1.9)
Site 2	0	1 (1.9)
Site 3	2 (5.0)	0
Site 4	1 (2.5)	0
**Mexico**		
All sites	5 (12.5)	7 (13.2)
Site 1	5 (12.5)	7 (13.2)
**Peru**		
All sites	6 (15.0)	8 (15.0)
Site 1	2 (5.0)	2 (3.8)
Site 2	1 (2.5)	4 (7.5)
Site 3	3 (7.5)	2 (3.8)
**Poland**		
All sites	2 (5.0)	0
Site 1	1 (2.5)	0
Site 2	1 (2.5)	0
**Russian Federation**		
All sites	5 (12.5)	6 (11.3)
Site 1	0	1 (1.9)
Site 2	2 (5.0)	3 (5.7)
Site 3	3 (7.5)	2 (3.8)
**Spain**		
All sites	5 (12.5)	9 (17.0)
Site 1	2 (5.0)	4 (7.5)
Site 2	1 (2.5)	2 (3.8)
Site 3	2 (5.0)	3 (5.7)
**United Kingdom**		
All sites	2 (5.0)	3 (5.7)
Site 1	1 (2.5)	0
Site 2	0	1 (1.9)
Site 3	0	1 (1.9)
Site 4	1 (2.5)	1 (1.9)

Brunner HI, *et al. Ann Rheum Dis* 2020; 79:1340–1348. doi: 10.1136/annrheumdis-2020-217101

Supplementary File

**United States**		
All sites	4 (10.0)	8 (15.1)
Site 1	1 (2.5)	3 (5.7)
Site 2	0	1 (1.9)
Site 3	0	1 (1.9)
Site 4	1 (2.5)	2 (3.8)
Site 5	1 (2.5)	0
Site 6	1 (2.5)	1 (1.9)

*Patient transferred to site 1 post-randomisation

Brunner HI, *et al. Ann Rheum Dis* 2020; 79:1340–1348. doi: 10.1136/annrheumdis-2020-217101

Supplementary File

Table S2

Efficacy endpoints

**Primary endpoint**
SRI4 response rate at Week 52,[15,16] defined as:<br>• ≥4-point reduction from baseline in SELENA-SLEDAI score, *and*<br>• no worsening in PGA, i.e. PGA increase <0.30 points from baseline, *and*<br>• no new BILAG A organ domain score; and no two new BILAG B organ domain scores compared with baseline
**Major secondary endpoints**
Proportion of patients responding to therapy defined by PRINTO/ACR 30* or 50† cSLE criteria at Week 52,[17-19] which consider percentage changes from baseline of the five multi-dimensional core components:<br>• PGA (scale 0–3)<br>• Parent-global (scale 0–10)<br>• SELENA-SLEDAI<br>• PedsQL (physical-functioning domain, scale 0–100)<br>• Proteinuria
Proportion of patients with sustained response in SRI4 at Weeks 44, 48 and 52
Proportion of patients with sustained response in Parent-global (improvement of >0.7 [minimally clinically important difference]) at Weeks 44, 48 and 52
**Other efficacy endpoints**
Components of SRI at Week 52
SRI6 response rate (identical to SRI4, except for higher threshold of improvement for SELENA-SLEDAI ≥6) at Week 52
Time to first severe flare measured using the SLE Flare Index, modified to exclude the single criterion of increased SELENA–SLEDAI score to >12 [13,20]
Mean change from baseline in average daily corticosteroid dose and the proportion of patients with average corticosteroid dose reduction ≥25% from baseline to Weeks 44–52
Subgroup analysis of SRI4 response at Week 52 by baseline age
Percentage of patients with organ improvement by BILAG at Week 52 among patients with grade A or B domain score at baseline
Percentage of patients with organ worsening by BILAG at Week 52 among patients without grade A domain score at baseline
Percentage of patients with organ improvement by SELENA SLEDAI at Week 52 among patients with organ system involvement at baseline
Percentage of patients with organ worsening by SELENA SLEDAI at Week 52 among patients without organ system involvement at baseline
**Renal endpoints**
Proportion of patients with renal flare over 52 weeks among those with high proteinuria (>0.5 mg/mg) at baseline
Proteinuria shifts from high (>0.5 mg/mg) to normal (≤0.5 mg/mg) over 52 weeks

*PRINTO/ACR 30 defined as the proportion of patients with ≥30% improvement in three of five cSLE core response criteria and ≤1 of the remaining worsening by >30% [19]; †PRINTO/ACR 50 defined as the proportion of patients with ≥50% improvement in any two of five cSLE core response criteria and ≤1 of the remaining worsening by >30% [19].

Supplementary File

ACR, American College of Rheumatology; BILAG, British Isles Lupus Assessment Group; cSLE, childhood-onset systemic lupus erythematosus; Parent-global, Parent Global Assessment of patient overall well-being; PedsQL, Pediatric Quality of Life Inventory generic core scale; PGA, Physician's Global Assessment of cSLE activity; PRINTO, Paediatric Rheumatology International Trials Organisation; SELENA-SLEDAI, Safety of Estrogens in Lupus Erythematosus National Assessment-SLE Disease Activity Index; SRI, SLE Responder Index.

Brunner HI, *et al. Ann Rheum Dis* 2020; 79:1340–1348. doi: 10.1136/annrheumdis-2020-217101

Supplementary File

Table S3

Efficacy analyses – additional information on model covariates

**Endpoint**	**Model**	**Covariate**
SRI4 and SRI6 (Week 52)	Logistic regression	• Treatment group (belimumab vs placebo)<br>• Baseline age group (5–11 vs 12–17 years)<br>• Baseline SELENA-SLEDAI score (6–12 vs ≥13)
Components of SRI response		
SELENA-SLEDAI component	Logistic regression	• Treatment group (belimumab vs placebo)<br>• Baseline age group (5–11 vs 12–17 years)<br>• Baseline SELENA-SLEDAI score (6–12 vs ≥13)
PGA component	Logistic regression	• Treatment group (belimumab vs placebo)<br>• Baseline PGA score<br>• Baseline age group (5–11 vs 12–17 years)<br>• Baseline SELENA-SLEDAI score (6–12 vs ≥13)
BILAG component	Logistic regression	• Treatment group (belimumab vs placebo)<br>• Baseline BILAG organ domain involvement (at least 1A/2B vs at most 1B)<br>• Baseline age group (5–11 vs 12–17 years)<br>• Baseline SELENA-SLEDAI score (6–12 vs ≥13)
PRINTO/ACR 30 or 50 cSLE criteria (Week 52)	Logistic regression	• Treatment group (belimumab vs placebo)<br>• Baseline age group (5–11 vs 12–17 years)<br>• Baseline SELENA-SLEDAI score (6–12 vs ≥13)
Percentage change from baseline in Parent-global (Week 52)	ANCOVA	• Treatment group (belimumab vs placebo)<br>• Baseline Parent-global score<br>• Baseline age group (5–11 vs 12–17 years)<br>• Baseline SELENA-SLEDAI score (6–12 vs ≥13)
Percentage change from baseline in PGA (Week 52)	ANCOVA	• Treatment group (belimumab vs placebo)<br>• Baseline PGA score<br>• Baseline age group (5–11 vs 12–17 years)<br>• Baseline SELENA-SLEDAI score (6–12 vs ≥13)
Percentage change from baseline in SELENA-SLEDAI (Week 52)	ANCOVA	• Treatment group (belimumab vs placebo)<br>• Baseline age group (5–11 vs 12–17 years)<br>• Baseline SELENA-SLEDAI score (6–12 vs ≥13)
Percentage change from baseline in proteinuria (Week 52)	ANCOVA	• Treatment group (belimumab vs placebo)
Percentage change from baseline in PedsQL (physical functioning domain) (Week 52)	ANCOVA	• Treatment group (belimumab vs placebo)<br>• Baseline PedsQL score<br>• Baseline age group (5–11 vs 12–17 years)<br>• Baseline SELENA-SLEDAI score (6–12 vs ≥13)
Sustained SRI response (Weeks 44–52)	Logistic regression	• Treatment group (belimumab vs placebo)<br>• Baseline age group (5–11 vs 12–17 years)<br>• Baseline SELENA-SLEDAI score (6–12 vs ≥13)
Sustained Parent-global response (Weeks 44–52)	Logistic regression	• Treatment group (belimumab vs placebo)<br>• Baseline Parent-global score<br>• Baseline age group (5–11 vs 12–17 years)

Brunner HI, *et al. Ann Rheum Dis* 2020; 79:1340–1348. doi: 10.1136/annrheumdis-2020-217101

Supplementary File

		• Baseline SELENA-SLEDAI score (6–12 vs ≥13)
Time to first severe SFI flare	Cox proportional hazards	• Baseline age group (5–11 vs 12–17 years)<br>• Baseline SELENA-SLEDAI score (6–12 vs ≥13)
Average corticosteroid dose reduction ≥25% from baseline to Weeks 44–52	Logistic regression	• Treatment group (belimumab vs placebo)<br>• Baseline corticosteroid dose<br>• Baseline age group (5–11 vs 12–17 years)<br>• Baseline SELENA-SLEDAI score (6–12 vs ≥13)

ACR, American College of Rheumatology; ANCOVA, analysis of covariance; BILAG, British Isles Lupus Assessment Group; cSLE, childhood-onset systemic lupus erythematosus; Parent-global, Parent Global Assessment of patient overall well-being; PedsQL, Pediatric Quality of Life inventory generic core scale; PGA, Physician's Global Assessment of cSLE activity; PRINTO, Paediatric Rheumatology International Trials Organisation; SELENA-SLEDAI, Safety of Estrogens in Lupus Erythematosus National Assessment-SLE Disease Activity Index; SRI, SLE Responder Index.

Brunner HI, *et al. Ann Rheum Dis* 2020; 79:1340–1348. doi: 10.1136/annrheumdis-2020-217101

Supplementary File

Table S4

SELENA-SLEDAI organ involvement at baseline

**Organ Item**	**Placebo (N=40)**, n (%)	**Belimumab 10 mg/kg IV (N=53)**, n (%)
Mucocutaneous	35 (87.5)	50 (94.3)
Immunologic	28 (70.0)	41 (77.4)
Musculoskeletal	33 (82.5)	35 (66.0)
Renal	8 (20.0)	10 (18.9)
Cardiovascular and Respiratory	2 (5.0)	4 (7.5)
Haematologic	2 (5.0)	3 (5.7)
CNS	1 (2.5)	2 (3.8)
Vascular	1 (2.5)	2 (3.8)

CNS, central nervous system; IV, intravenous; SELENA-SLEDAI, Safety of Estrogens in Lupus Erythematosus National Assessment-Systemic Lupus Erythematosus Disease Activity Index.

Brunner HI, *et al. Ann Rheum Dis* 2020; 79:1340–1348. doi: 10.1136/annrheumdis-2020-217101

Supplementary File

Table S5

BILAG grade A and B by organ domain at baseline

Organ domain/grade	Placebo (N=40), n (%)	Belimumab 10 mg/kg IV (N=53), n (%)
General		
A	0	0
B	3 (7.5)	3 (5.7)
Mucocutaneous		
A	3 (7.5)	3 (5.7)
B	24 (60.0)	40 (75.5)
Neurological		
A	0	0
B	0	0
Musculoskeletal		
A	2 (5.0)	0
B	29 (72.5)	33 (62.3)
Cardiovascular & Respiratory		
A	0	0
B	0	1 (1.9)
Vasculitis		
A	0	0
B	2 (5.0)	8 (15.1)
Renal		
A	1 (2.5)	1 (1.9)
B	5 (12.5)	7 (13.2)
Haematology		
A	0	0
B	9 (22.5)	2 (3.8)

Organ domain grades: A=requires disease modifying treatment, B=mild reversible problems requiring only symptomatic therapy.

BILAG, British Isles Lupus Assessment Group; IV, intravenous.

Brunner HI, *et al. Ann Rheum Dis* 2020; 79:1340–1348. doi: 10.1136/annrheumdis-2020-217101

Supplementary File

Table S6

ACR classification criteria reported for at least 50% of patients at baseline

**ACR classification criteria**	**Placebo (N=40)**, n (%)	**Belimumab 10 mg/kg IV (N=53)**, n (%)
Antinuclear antibody positivity	39 (97.5)	53 (100.0)
Arthritis	36 (90.0)	43 (81.1)
Immunologic disorder	32 (80.0)	45 (84.9)
Malar "butterfly" rash	31 (77.5)	45 (84.9)
Anti-DNA antibody positivity	26 (65.0)	39 (73.6)
Photosensitivity	20 (50.0)	39 (73.6)
Haematologic disorder	27 (67.5)	26 (49.1)
Mucosal ulcers	19 (47.5)	30 (56.6)

ACR, American College of Rheumatology; DNA, deoxyribonucleic acid; IV, intravenous.

Brunner HI, *et al. Ann Rheum Dis* 2020; 79:1340–1348. doi: 10.1136/annrheumdis-2020-217101

Supplementary File

Table S7

Percentage change from baseline in biomarkers at Week 52

Median (IQR) percentage change	Placebo (N=40)	Belimumab 10 mg/kg IV (N=53)
IgG	2.5 (–9.03, 22.42)	–17.7 (–25.74, –7.78)
Anti-dsDNA antibody*	2.2 (–34.89, 54.76)	–44.9 (–67.31, 33.78)
Complement C3[†]	6.0 (–17.50, 15.00)	17.3 (–9.72, 43.42)
Complement C4[†]	18.1 (0.00, 47.22)	50.0 (28.57, 100.00)
CD19+	–18.9 (–46.15, 25.36)	–63.7 (–77.32, –50.70)
CD20+	–18.6 (–45.36, 30.50)	–65.8 (–77.23, –53.62)
Naïve (CD19+/CD20+/CD27-)	–26.7 (–50.61, 25.46)	–77.1 (–84.48, –63.27)
Memory (CD19+/CD20+/CD27+)	0.0 (–29.17, 61.89)	11.7 (–24.44, 42.86)

*For patients anti-dsDNA positive (≥30 IU/mL) at baseline; [†]for patients with low complement at baseline.
C, component; dsDNA, double-stranded DNA; IgG, immunoglobulin G; IQR, interquartile range; IV, intravenous.

Brunner HI, *et al. Ann Rheum Dis* 2020; 79:1340–1348. doi: 10.1136/annrheumdis-2020-217101

Supplementary File

Figure S1

Study design

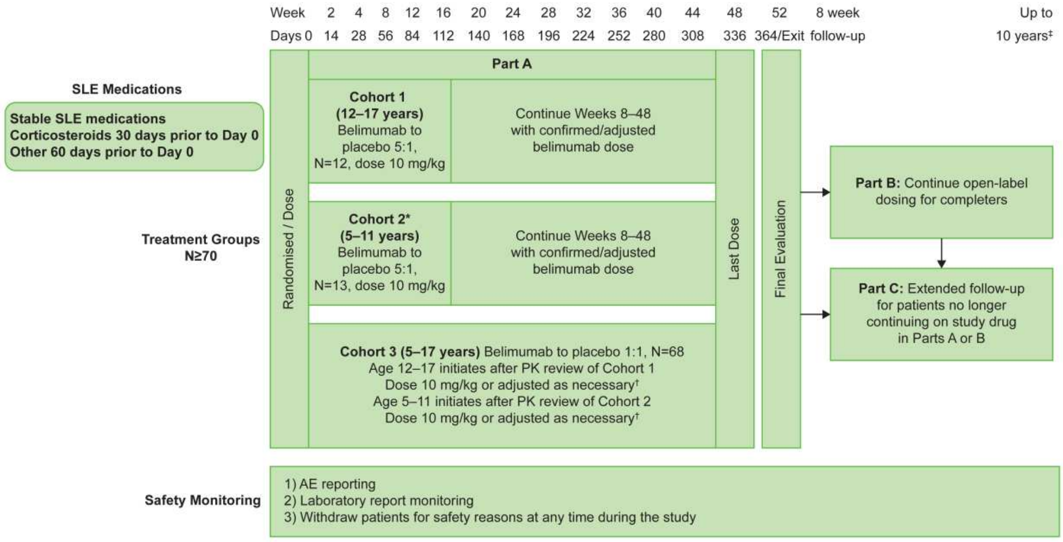

*Initiate Cohort 2 after confirmed/adjusted dose from Cohort 1 PK review; †PK group will remain blinded, regardless of outcome; ‡may conclude earlier if the last patient has completed ≥5 years of treatment with belimumab and number of patients continuing with belimumab is <15.

AE, adverse event; PK, pharmacokinetics; SLE, systemic lupus erythematosus.

Brunner HI, *et al. Ann Rheum Dis* 2020; 79:1340–1348. doi: 10.1136/annrheumdis-2020-217101

Supplementary File

Figure S2

Permitted medications during the study

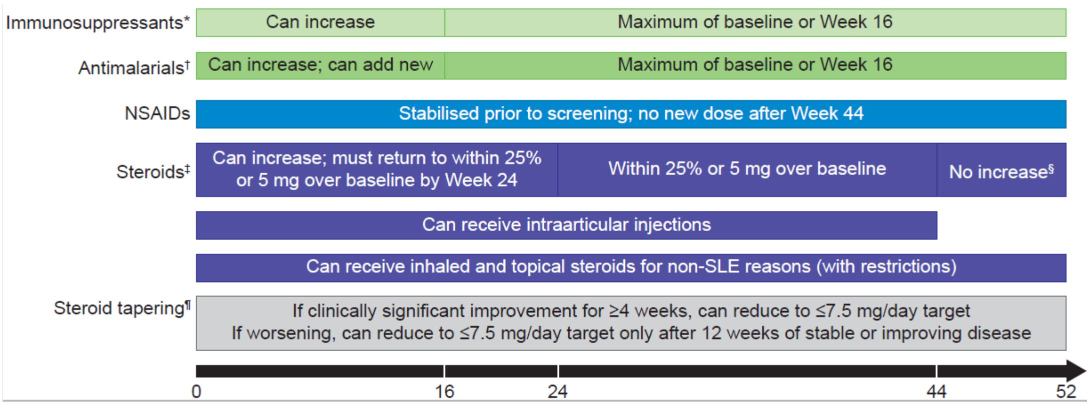

*Allowable doses: azathioprine ≤300 mg/day, 6-mercaptopurine ≤300 mg/day, mycophenolate mofetil/mycophenolate mofetil hydrochloride ≤4 g/day, mycophenolate sodium ≤2.88 g/day, methotrexate ≤25 mg/week, oral cyclophosphamide ≤2.5 mg/kg/day, cyclosporine ≤4 mg/kg/day, tacrolimus ≤0.2 mg/kg/day, sirolimus ≤2 mg/day, thalidomide ≤200 mg/day, leflunomide ≤40 mg/day, mizoribine ≤150 mg/day; †allowable doses: hydroxychloroquine ≤400 mg/day, chloroquine ≤500 mg/day, quinacrine ≤100 mg/day, compounded antimalarials: no individual component may exceed the maximum dose; ‡includes intravenous, intramuscular, subcutaneous, intradermal, and oral; systemic dose is defined as the average daily dose of all routes of administration; §over baseline or Week 44 dose; ¶at the investigator's discretion.
NSAID, non-steroidal anti-inflammatory drug; SLE, systemic lupus erythematosus.

Brunner HI, *et al. Ann Rheum Dis* 2020; 79:1340–1348. doi: 10.1136/annrheumdis-2020-217101

Supplementary File

Figure S3

SRI4 response at Week 52 by baseline age group

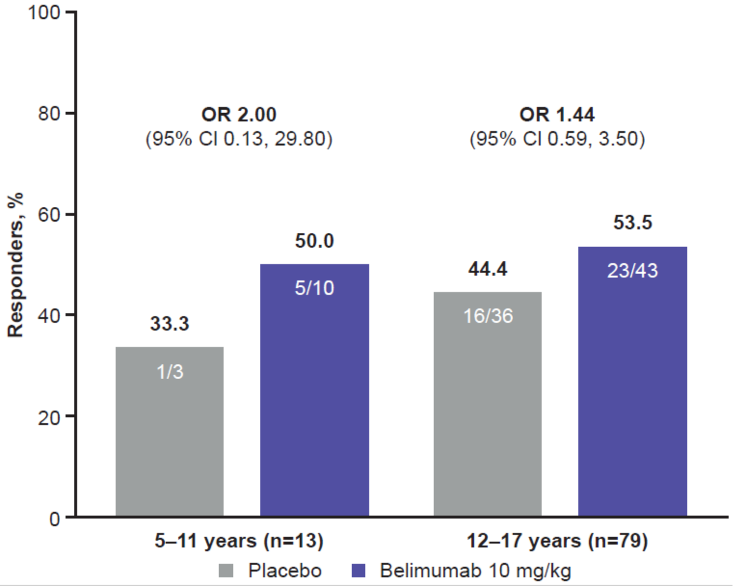

CI, confidence interval; OR, odds ratio; SLE, systemic lupus erythematosus; SRI4, SLE Responder Index 4.

Brunner HI, *et al. Ann Rheum Dis* 2020; 79:1340–1348. doi: 10.1136/annrheumdis-2020-217101

Supplementary File

Figure S4

Median belimumab concentrations (µg/mL; all cohorts, observed, Part A)

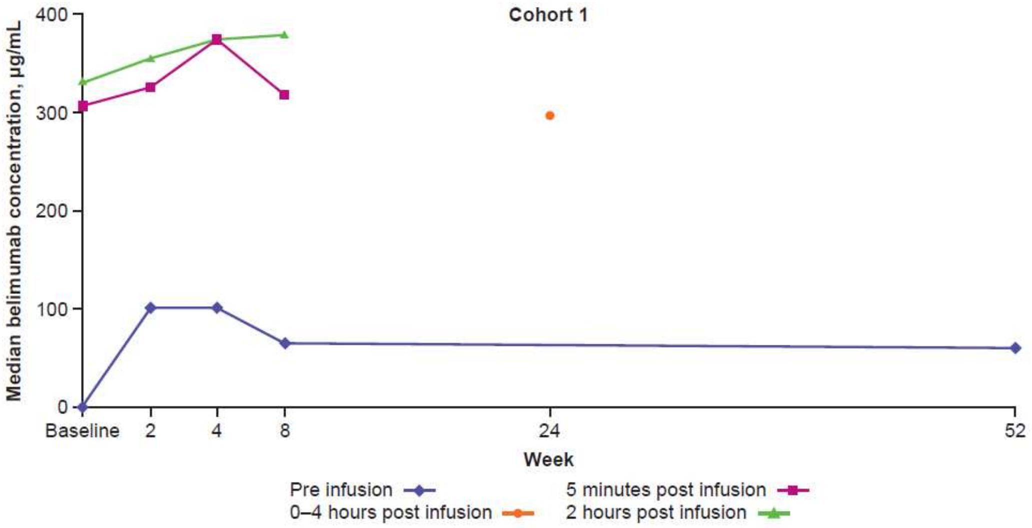

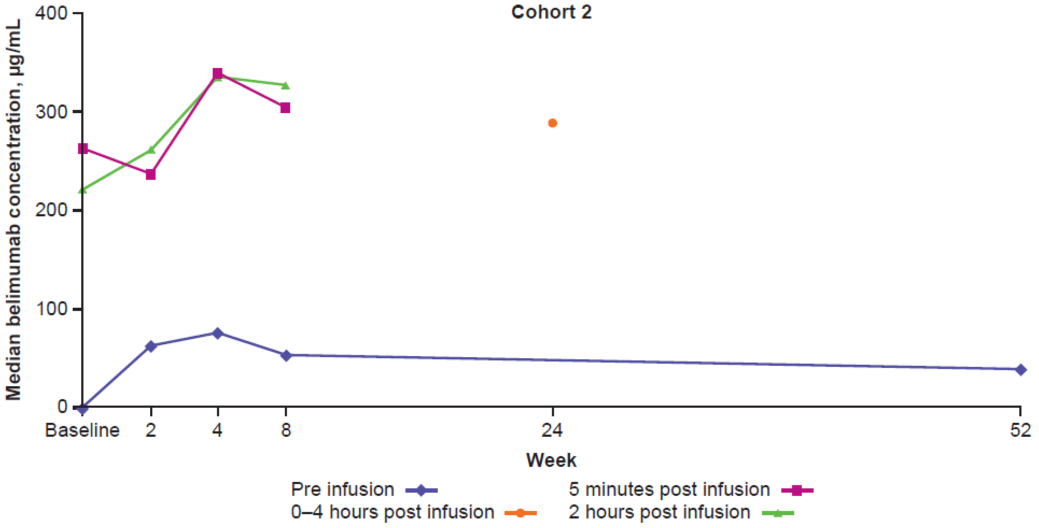

Brunner HI, *et al. Ann Rheum Dis* 2020; 79:1340–1348. doi: 10.1136/annrheumdis-2020-217101

Supplementary File

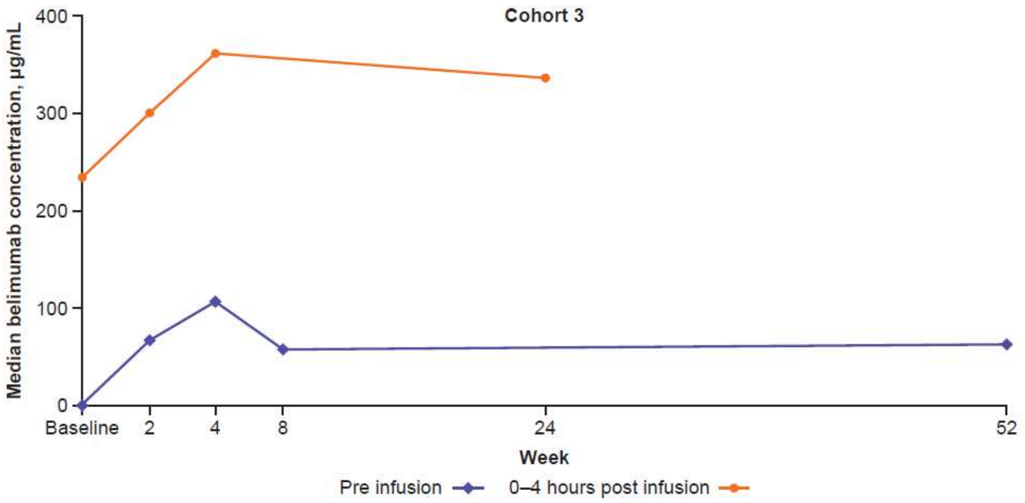

Belimumab 10 mg/kg IV was administered on Days 0, 14 and 28 (loading doses) and then every 28 days until Week 48. For all 3 cohorts, pre infusion (C_{min}) median serum belimumab concentrations reached steady-state levels early in the study (by Week 8) and were maintained throughout the 52-week treatment period. Post infusion (C_{max}) median serum belimumab concentrations, measured at 5 minutes or 2 hours after the end of the infusion (Cohorts 1 and 2), or within the interval of 0–4 hours post infusion (Cohort 3), also rapidly increased to steady-state levels over the first 8 weeks of treatment. The results are consistent with adult data[27].
C_{max}, maximum plasma concentration; C_{min}, minimum plasma concentration; IV, intravenous.

Brunner HI, *et al. Ann Rheum Dis* 2020; 79:1340–1348. doi: 10.1136/annrheumdis-2020-217101
