# Supplementary material for: Safety and efficacy of intravenous belimumab in children with systemic lupus erythematosus: results from a randomised, placebo-controlled trial
Source: Ann Rheum Dis. 2020 Jul 22;79(10):1340–8. doi: 10.1136/annrheumdis-2020-217101 (PMC7509523; doi:10.1136/annrheumdis-2020-217101)
Supplement: Supplementary data [file annrheumdis-2020-217101supp002.pdf]

Protocol and Bayesian analysis report

The safety and efficacy of intravenous belimumab in children with systemic lupus erythematosus: Results from a randomized, placebo-controlled trial

Hermine I Brunner, Carlos Abud-Mendoza, Diego O Viola, Inmaculada Calvo Penades, Deborah M Levy, Jordi Anton, Julia E Calderon, Vyacheslav G Chasnyk, Manuel A Ferrandiz, Vladimir A Keltsev, Maria E Paz Gastanaga, Michael Shishov, Alina Lucica Boteanu, Michael Henrickson, Damon Bass, Kenneth Clark, Anne Hammer, Beulah Ji, Antonio Nino, David A Roth, Herbert Struemper, Mei-Lun Wang, Alberto Martini, Daniel J Lovell, Nicolino Ruperto in collaboration with the Paediatric Rheumatology International Trials Organisation (PRINTO) and the Pediatric Rheumatology Collaborative Study Group (PRCSG)

This supplement contains the following items: Page

Study protocol …………………………..….…………………………………………..…………2
Bayesian extrapolation analysis report……………………………………………………………...135

TITLE PAGE

Division: Worldwide Development
Information Type: Protocol Amendment

**Title:**	A Multi-center, Randomized Parallel Group, Placebo-Controlled Double-Blind Trial to Evaluate the Safety, Efficacy, and Pharmacokinetics of Belimumab, a Human Monoclonal Anti-BLyS Antibody, Plus Standard Therapy in Pediatric Patients with Systemic Lupus Erythematosus (SLE)

Compound Number: GSK1550188

Development Phase: II

Effective Date: 12-DEC-2016

Protocol Amendment Number: 06

Author (s):

PPD (Immuno-Inflammation & Neurosciences, Projects Clinical Platforms and Sciences; PPD (Immuno-Inflammation Clinical Development); PPD (Clinical Statistics, Immuno-Inflammation); PPD (Immuno-Inflammation & Neurosciences, Projects Clinical Platforms and Sciences); PPD (Safety Evaluation & Risk Management, Global Clinical Safety and Pharmacovigilance); PPD (Safety Evaluation & Risk Management Global Clinical Safety and Pharmacovigilance); PPD (Quantitative Clinical Developent , PAREXEL on behalf of Clinical Pharmacology Modelling & Simulation); PPD (Value Evidence and Outcomes); PPD (Global Supplies Chain Management); PPD (Immuno-Inflammation, Clinical Development), PPD (Immuno-Inflammation, Clinical Development), PPD (Immuno-Inflammation, Clinical Development)

Copyright 2016 the GlaxoSmithKline group of companies. All rights reserved. Unauthorised copying or use of this information is prohibited.

Revision Chronology

GlaxoSmithKline Document Number	Date	Version
2010N108742_01	2011-AUG-24	Original
2010N108742_02	2012-FEB-21	Amendment No. 1
Update Author information<br>Update Medical Monitor information<br>Additional pharmacokinetic samples for children not in Cohort 1 or 2<br>Addition of three hours observation post infusion<br>Update to Time and Events Table including immunogenicity, vaccine titer procedure and physical assessments<br>Update Other Secondary Endpoints Addition of Study Conclusion criteria<br>Minor typographical errors corrected		
2010N108742_03	2013-JAN-31	Amendment No. 2
Country Specific Amendment for Russian Federation: only subjects ≥12 years of age may participate.<br>Amendment 02 pertaining to all sites includes the following changes:<br>Exclusion Criterion #1 excludes if B cell targeted therapy is within 1 year of Day 0 and Exclusion #23 omitting HCV confirmation RIBA assay.<br>Allow extra visits for collection of large blood sample collection<br>Addition of 3 hours observation post infusion for first 3 infusions in Part B.<br>The investigational product may be delivered in either 100 mL or 250mL of saline.<br>Minor typographical errors corrected.		
2010N108742_04	2014-FEB-18	Amendment No. 3
Amendment No: 03 pertaining to all sites:<br><br>Update to safety information on PML and Delayed Hypersensitivity Reaction.<br>Changed the exclusion for high dose steroid use from 90 days to 60 days prior to baseline.<br>Clarification of treatment failure and study withdrawal criteria<br>Clarification of procedure for destruction of investigational product and used vials.<br>Added a home pregnancy test and follow up phone call at 16 Week post last dose.<br>Extended visit window in Part B and Part C<br>Minor typographical and formatting errors corrected.		
2010N108742_05	2014-NOV-03	Amendment No. 4
Amendment No: 04<br><br>Changed the Screening inclusion criterion SELENA SLEDAI to ≥ 6.		

Changed randomization strata for Cohort 3 to stratification by age and SELENA SLEDAI 6-12 and ≥13.<br>Added requirement that 50% of subjects will be recruited with SS score of ≥ 8.<br>Changed exclusion #4 intravenous (IV) cyclophosphamide within 60 days of Day 0.<br>Added allowance for stable Grade 3 lymphopenia with exclusion #24.<br>Updated GSK liver chemistry stopping criteria and added of study drug restart criteria<br>Clarification of laboratory tests needed to complete SS and BILAG assessments. Added immunogenicity testing in Part C.<br>Clarification of NSAIDs use in Part A<br>Minor typographical errors corrected		
2010N108742_06	2015-APR-22	Amendment No. 5
Country Specific Amendment for Russian Federation: Restart of study treatment after liver event is not applicable to Investigator sites in Russia.<br>Updated phone number of back-up Medical Monitor.		
2010N108742_07	2016-DEC-12	Amendment No. 6
Updated Author information<br>Updated Medical Monitor information<br>Updated site address<br>Total study target amended to ‘at least 70’ from 100 subjects.<br>Estimates for sample size re-estimation add to the protocol.<br>Target for subjects less than 13 years old amended to at least 14 from 20.<br>Target for Cohort 2 amended from 12 to “at least 10”.<br>PedsQL amended in time and events table such that it is instructed to be taken at Day 0.<br>Anti-dsDNA; C3/C4 added to time and events table for 6 month visits in Part B.<br>Deleted immunogenicity sample collection once a subject has left the study.<br>Collect additional PK samples in the subjects in Japan at the first 12 week and 6 month visit in Part B<br>Minor typographical errors corrected<br>Clarification of reporting of AEs, SAEs, and AESI<br>Change in Part C from “non-belimumab phase” to “safety follow up phase”		

SPONSOR SIGNATORY

PPD

12 Dec 2016

Beulah Ji, MD   Date

Physician Project Leader
Director, Clinical Development
GlaxoSmithKline

PPD

SPONSOR INFORMATION PAGE

Clinical Study Identifier: BEL114055

GlaxoSmithKline
Iron Bridge Road
Stockley Park West, Uxbridge, Middlesex, UB11 1BU, UK
Telephone: PPD

GlaxoSmithKline
Research and Development
1250 South Collegville Road
Collegeville, PA 19146-0989, USA
Telephone: PPD

GlaxoSmithKline
Five Moore Drive
P.O. 13398
Research Triangle Park, NC 27709-3398, USA
Telephone: PPD

In some countries, the clinical trial sponsor may be the local GlaxoSmithKline Affiliate Company (or designee). Where applicable, the details of the Sponsor and contact person will be provided to the relevant regulatory authority as part of the clinical trial submission.

Sponsor Medical Monitor Contact Information:	Back-up Sponsor Medical Monitor Contact Information
PPD MD<br>GlaxoSmithKline<br><br>Research & Development<br>1250 South Collegeville Road<br>UP 1230<br>Collegeville, PA 19426<br>USA<br><br>Mobile: PPD<br>Email: PPD	PPD MD<br>GlaxoSmithKline<br>Stockley Park West,<br>1-3 Ironbridge Road, Uxbridge,<br>Middlesex, UB11 1BT,<br>United Kingdom<br>Telephone: PPD<br>Fax: PPD<br>Mobile: PPD<br>Email: PPD

Sponsor Serious Adverse Events (SAE) Contact Information:
Case Management Group, Global Clinical Safety and Pharmacovigilance (GCSP)
Email: PPD
Fax: PPD

United States Food and Drug Administration Identifying Number(s): IND 009970 (Human Genome Sciences, Inc)

Eudract number: 2011-000368-88

Brunner HI, *et al. Ann Rheum Dis* 2020; 79:1340–1348. doi: 10.1136/annrheumdis-2020-217101

INVESTIGATOR PROTOCOL AGREEMENT PAGE

For protocol amendment 6

I confirm agreement to conduct the study in compliance with the protocol, as amended by this protocol amendment.

I acknowledge that I am responsible for overall study conduct. I agree to personally conduct or supervise the described study.

I agree to ensure that all associates, colleagues and employees assisting in the conduct of the study are informed about their obligations. Mechanisms are in place to ensure that site staff receives the appropriate information throughout the study.

Investigator Name:	
Investigator Address:	
Investigator Phone Number:	
Investigator Signature	Date

TABLE OF CONTENTS

PAGE

LIST OF ABBREVIATIONS 11

PROTOCOL SUMMARY 14

1. INTRODUCTION 19
   1.1. Disease Background 19
   1.2. Rationale 23
      1.2.1. Dose Rationale 24

2. OBJECTIVES 25

3. INVESTIGATIONAL PLAN 26
   3.1. Study Design 26

4. SUBJECT SELECTION AND WITHDRAWAL CRITERIA 30
   4.1. Number of Subjects 30
   4.2. Inclusion Criteria 30
   4.3. Exclusion Criteria 33
   4.4. Withdrawal Criteria 36

5. STUDY TREATMENTS 37
   5.1. Study Agent and Other Study Treatment 37
   5.2. Treatment Assignment 39
   5.3. Blinding 40
   5.4. Unblinding 40
   5.5. Product Accountability 41
   5.6. Treatment Compliance 41
      5.6.1. Permitted Medications and Non-Drug Therapies 41
         5.6.1.1. Anti-malarials 41
         5.6.1.2. Steroids 42
            5.6.1.2.1. Systemic Steroids for SLE-related Disease Activity 42
            5.6.1.2.2. Intra-articular Injections 42
            5.6.1.2.3. Steroids for Reasons Other Than SLE Disease Activity 43
            5.6.1.2.4. Tapering or Restoration/Restart of Steroids 43
            5.6.1.2.5. Treatment of SLE Flares with Steroids 44
         5.6.1.3. Other Immunosuppressive/Immunomodulatory Agents 44
         5.6.1.4. Nonsteroidal Anti-inflammatory Drugs (NSAIDs) 45
      5.6.2. Prohibited Medications and Non-Drug Therapies 46
   5.7. Treatment after the End of the Study 46
      5.7.1. Study Conclusion 47
   5.8. Treatment of Study Treatment Overdose 47

6. STUDY ASSESSMENTS AND PROCEDURES 47
6.1. Critical Screening and Baseline Assessments 58
6.2. Efficacy 59
6.2.1. SELENA SLEDAI 59
6.2.1.1. SELENA SLEDAI Score 59
6.2.1.2. Laboratory Tests for SELENA SLEDAI and BILAG 60
6.2.1.3. Guidelines for Scoring Proteinuria for SELENA SLEDAI 60
6.2.1.3.1. Scoring for Proteinuria for Eligibility and at Screening 61
6.2.1.3.2. Scoring for Proteinuria at Day 0 and Subsequent Study Visits 61
6.2.1.4. SELENA SLEDAI Flare Index 62
6.2.1.5. Physician’s Global Assessment (PGA) 62
6.2.1.6. Parent ’s Global Assessment (ParentGA) 62
6.2.2. Pediatric Rheumatology International Trials Organization/ACR (PRINTO/ACR Juvenile SLE Response Evaluation) 63
6.2.3. BILAG 63
6.2.4. Pediatric SLICC/ACR Damage Index 64
6.3. Safety 64
6.3.1. Liver Chemistry Stopping and Monitoring Criteria 65
6.3.1.1. Required Actions and Follow up Assessments following ANY Liver Stopping Event 66
6.3.2. Increased Monitoring Criteria with Continued Therapy 67
6.3.2.1. Required Actions and Follow Up Assessments for Increased Monitoring with Continued Therapy 68
6.3.3. Study Treatment Restart 68
6.3.4. IgG Stopping Criteria 69
6.3.5. Independent Data Monitoring Committee 69
6.3.6. Adverse Events 70
6.3.6.1. Definition of an AE 70
6.3.6.2. Definition of a SAE 71
6.3.7. Laboratory and Other Safety Assessment Abnormalities Reported as AEs and SAEs 72
6.3.8. Disease-Related Events and/or Disease-Related Outcomes Not Qualifying as SAEs 72
6.3.9. Suicidality Assessment 73
6.3.10. Possible Suicidality Related Questionnaire (PSRQ) 74
6.3.11. Investigator Evaluation of Adverse Events 74
6.3.12. Pregnancy 74
6.3.13. Time Period and Frequency of Detecting AEs and SAEs 75
6.3.14. Prompt Reporting of Serious Adverse Events and Other Events to GSK 75
6.3.14.1. Regulatory reporting requirements for SAEs 76
6.3.15. Laboratory Evaluations 77
6.3.16. Infusion-related Reactions and Hypersensitivity Reactions 77
6.3.17. Immunosuppression, Infections, and Malignancies 78
6.3.18. Vital Signs, Height and Weight 79
6.3.19. Physical Examination 79

6.3.20. Other Safety Outcomes 80
6.3.20.1. Immunogenicity 80
6.4. Health Outcomes 80
6.4.1. Pediatric Quality of Life Inventory - Generic Core (PedsQL) 80
6.4.2. Pediatric Quality of Life Multidimensional Fatigue Scale 81
6.5. Pharmacokinetics 81
6.6. Pharmacodynamics 83
6.6.1. Biomarker(s) 83
6.6.2. Test for Functional Antibodies 84
6.6.3. Vaccine Response 84
6.7. Pharmacogenetics 84

7. DATA MANAGEMENT 85

8. DATA ANALYSIS AND STATISTICAL CONSIDERATIONS 85
8.1. Hypotheses 85
8.2. Study Design Considerations 85
8.2.1. Sample Size Considerations 85
8.2.2. Sample Size Sensitivity 85
8.2.3. Sample Size Re-estimation 86
8.3. Data Analysis Considerations 86
8.3.1. Analysis Populations 86
8.3.2. Analysis Data Sets 87
8.3.3. Analysis strategy and timing 87
8.3.4. Interim Analysis 87
8.3.5. Key Elements of Analysis Plan 88
8.3.5.1. Laboratory Data for Disease Activity Scales 89
8.3.5.2. Efficacy Analyses 89
8.3.5.2.1. Primary Efficacy Endpoint 90
8.3.5.2.2. Major Secondary Efficacy Endpoints 91
8.3.5.3. Safety Analyses 92
8.3.5.3.1. Extent of Exposure 92
8.3.5.3.2. Adverse Events and Disease Related Events 93
8.3.5.3.3. Laboratory Parameters 93
8.3.5.3.4. Vital Signs 93
8.3.5.3.5. Immunogenicity 94
8.3.5.4. Pharmacokinetic Analyses 94
8.3.5.5. Pharmacogenetic Analyses 94
8.3.5.6. Biological Markers 94

9. STUDY CONDUCT CONSIDERATIONS 95
9.1. Posting of Information on Clinicaltrials.gov 95
9.2. Regulatory and Ethical Considerations, Including the Informed Consent Process 95
9.3. Quality Control (Study Monitoring) 95
9.4. Quality Assurance 96
9.5. Study and Site Closure 96
9.6. Records Retention 96

9.7. Provision of Study Results to Investigators, Posting to the Clinical Trials Register and Publication 97
9.8. Independent Data Monitoring Committee (IDMC) 97

10. REFERENCES 98

11. APPENDICES 106
11.1. Appendix 1: Pharmacogenetic Research 106
11.2. Appendix 2: Country Specific Requirements 110
11.3. Appendix 3: American College of Rheumatology (ACR) Criteria for SLE 111
11.4. Appendix 4: Liver Safety Criteria 112
11.4.1. Liver Stopping Event Algorithm 112
11.4.2. Liver Monitoring Event Algorithm with Continued Therapy for ALT≥3xULN but <8xULN 113
11.5. Appendix 5: Clinical Laboratory Tests 114
11.6. Appendix 6: SELENA SLEDAI Disease Assessment Scales 123
11.7. Appendix 7: SLE Flare Index 124
11.8. Appendix 8: Physician's Global Disease Assessment 125
11.9. Appendix 9: Parent's Global Disease Assessment 126
11.10. Appendix 10: BILAG Index Assessment 127
11.11. Appendix 11: Pediatric SLICC/ACR Damage Index 129
11.12. Appendix 12: Columbia Suicide Severity Rating Scale C-SSRS 133
11.13. Appendix 13: Possible Suicidality-Related Questionnaire 161
11.14. Appendix 14: Pediatric Quality of Life Inventory - Generic Core Scale (PedsQL) 163
11.15. Appendix 15: Pediatric Quality of Life Multidimensional Fatigue Scale (PedsQL Fatigue) 164
11.16. Appendix 16: Protocol Amendment 01 Changes 168
11.17. Appendix 17: Country Specific Protocol Amendment 02 Changes 187
11.18. Appendix 18: Protocol Amendment 02 Changes 188
11.19. Appendix 19: Protocol Amendment 03 - Summary of Changes 195
11.20. Appendix 20: Protocol Amendment 04 - Summary of Changes 203
11.21. Appendix 21: Country Specific Protocol Amendment 05 Changes 211
11.22. Appendix 22: Protocol Amendment 06 Changes 212

LIST OF ABBREVIATIONS

aCL	Anticardiolipin
ACR	American College of Rheumatology
AE	Adverse Event
ALT	alanine amininotransferase
ANA	anti-nuclear antibody
ANCOVA	Analysis of Covariance
AS	All subjects
AST	aspartate aminotransferase
BILAG	British Isles Lupus Assessment Group
BLyS	B lymphocyte Stimulator
CBC	Complete blood count
C-SSRS	Columbia Suicide Severity Rating Scale
CNS	Central Nervous System
CPK	Creatinine phosphokinase
CrCl	Creatinine clearance
CRF	Case report form
CVA	Cerebrovascular accident
dL	Deciliter
DRE	Disease Related Event
dsDNA	double stranded deoxyribonucleic acid
eCRF	electronic case report form
ECG	electrocardiogram
ELISA	enzyme linked immunosorbent assay
GCP	Good Clinical Practice
GCSP	Global Clinical Safety and Pharmacovigilance
GFR	Glomerular filtration rate
GSK	GlaxoSmithKline
HB	Hepatitis B
HBsAg	Hepatitis B surface antigen
HBc	Hepatitis B core
HEp-2	Human Epithelial Cell Line 2
HGS	Human Genome Sciences, Inc
HIV	Human immunodeficiency virus
HMG CoA	3-Hydroxy-3-Methyl-Glutaryl-Coenzyme A
hpf	High power field
IA	Intraarticular
IB	Investigator's Brochure
IDMC	Independent Data Monitoring Committee
IEC	Independent ethics committee
IgA	Immunoglobulin A
IgG	Immunoglobulin G
IgM	Immunoglobulin M
IL-6	Interleukin-6
IM	Intramuscular

INR	International Normalized Ratio
IRB	Institutional Review Board
ITT	Intention to Treat
IUD	intrauterine device
IV	Intravenous
IVIG	Intravenous immunoglobulin
IVRS	Interactive voice response system
kg	Kilogram
LDH	lactate dehydrogenase
LOCF	Last observation carried forward
MedDRA	Medical Dictionary for Drug Regulatory Activities
mg	Milligram
mL	Milliliter
MMF	Mycophenolate mofetil
MRI	Magnetic Resonance Imagery
MSDS	Materials Safety Data Sheet
NR	Non-responder
NSAIDs	non-steroidal anti-inflammatory drugs
ParentGA	Parent's Global Assessment
PedsQL	Pediatric Quality of Life Inventory – Generic Core Scale
PedsQL-Fatigue	Pediatric Quality of Life Multidimensional Fatigue Scale
PCR	polymerase chain reaction
PGA	Physician's Global Assessment
PGx	Pharmacogenetics
PO	By mouth (per os)
PRINTO	Pediatric Rheumatology International Trials Organization
PSRQ	Possible Suicidality-Related Questionnaire
PT	prothrombin time
PTT	partial thromboplastin time
RA	Rheumatoid Arthritis
RAP	Reporting and Analysis Plan
RBC	Red blood cell
RF	Rheumatoid factor
RNA	Ribonucleic acid
SAE	Serious Adverse Event
SELENA	Safety of Estrogen in Lupus National Assessment
SFI	SLE Flare Index
SLE	Systemic Lupus Erythematosus
SLEDAI	Systemic Lupus Erythematosus Disease Activity Index
SLICC	Systemic Lupus International Collaborating Clinics
SOC	System Organ Classes
SPM	Study Procedures Manual
SRI	SLE Responder Index
SRT	Safety Review Team
SWFI	Sterile water for injection

Brunner HI, *et al*. *Ann Rheum Dis* 2020; 79:1340–1348. doi: 10.1136/annrheumdis-2020-217101

TACI Fc	transmembrane activator attached to the Fc portion of an immunoglobulin
TF	Treatment Failure
TNF	Tumour Necrosis Factor
ULN	Upper limit of normal
VAS	Visual analogue scale

Trademark Information

Trademarks of the GlaxoSmithKline group of companies
BENLYSTA (belimumab)
LYMPHOSTAT-B

Trademarks not owned by the GlaxoSmithKline group of companies
PASS 2005

PROTOCOL SUMMARY

Rationale

The purpose of this study is to evaluate the safety, efficacy, pharmacokinetics, and the effects on quality of life of 10 mg/kg (or pediatric exposure adjusted dose) of belimumab administered intravenously (IV) compared to placebo in pediatric subjects 5 to 17 years of age with systemic lupus erythematosus (SLE) on a background of standard of care therapy. This study has a 52-week, randomized, double-blind, placebo-controlled phase followed by an open label continuing phase for up to 10 years for subjects that qualify. Additionally, subjects who withdraw from treatment at any time during the study will be assessed in a long term follow-up period where safety evaluations and limited disease activity assessments will be performed for up to 10 years.

Objectives

- Evaluate the safety and tolerability of belimumab in the pediatric SLE population
- Evaluate the pharmacokinetics of belimumab in the pediatric SLE population.
- Evaluate the efficacy of belimumab in the pediatric SLE population
- Evaluate the effects of belimumab on the quality of life in the pediatric SLE population.

Study Design

This is a multi-center study to evaluate the safety, efficacy and pharmacokinetics of belimumab plus background standard therapy in at least 70 pediatric subjects 5 years to 17 years of age with active systemic lupus erythematosus (SLE). The study will consist of three separate phases:

- Randomized, placebo-controlled, double-blind 52-week treatment phase (Part A)
- Long term belimumab open label safety follow up for any subject who completes Part A (Part B)
- Long term safety follow up phase for subjects who withdraw from Part A or Part B at any time (Part C)

Part A is a randomized, placebo-controlled, double-blind study to evaluate the efficacy, safety, and pharmacokinetics of 10 mg/kg belimumab intravenous (IV) in pediatric subjects with active SLE (SELENA SLEDAI score ≥6). In this study, at least 70 subjects will be randomized in three cohorts with Cohort 3 stratified by age (5-11 years vs. 12-17 years) and screening SELENA SLEDAI scores (6-12 vs. ≥13). Cohorts 1 and 2 will be randomized in a 5:1 ratio, and Cohort 3 subjects will be randomized in a 1:1 ratio to receive belimumab or placebo for 48 weeks on a background of standard of care. Cohort 1 will consist of the first 12 subjects, age 12 to 17 years. No further enrolment of the study will proceed until after the PK of this cohort is evaluated. Once the PK and safety profile of Cohort 1 is evaluated and any potential dose adjustments are determined as a

result of this analysis, additional subjects 12-17 years of age will then be enrolled in Cohort 3. In addition, at this point, at least 10 subjects 5-11 years of age at the dose determined from the PK analysis of Cohort 1. Once the PK and safety profile of Cohort 2 is evaluated and any potential dose adjustments are determined as a result of this analysis, additional subjects 5-11 years of age will then be enrolled in Cohort 3.

Belimumab will be infused over a minimum of 1 hour on Days 0, 14, 28, and then every 28 days through the Week 48 (Day 336) visit. All subjects will continue to receive their standard SLE therapy with progressive restrictions on the changes that are permitted throughout the 52-week randomized period. Enrolment will be staggered to allow PK analysis of the first 2 age cohorts (Cohorts 1 and 2).

Safety monitoring and PK analysis from the initial 12 subjects will be used to confirm or adjust the belimumab dose for the remaining subjects enrolling in Cohort 2 and 3.

- **Cohort 1:** The first 12 subjects, age 12 to 17 years of age will be randomized in a 5:1 ratio to belimumab 10 mg/kg (n=10) or placebo (n=2) on a background of standard of care for 48 weeks. After all 12 subjects in Cohort 1 have received at least 8 weeks of treatment, safety and PK analysis will be conducted. Cohort 1 subjects will continue in the treatment period while the Study Team progresses the PK analysis, but no other subjects will enrol until the PK analysis is completed. If the belimumab dose is adjusted because of the PK analysis, the Cohort 1 subjects will continue the study with the adjusted dose. Enrolment will be initiated for Cohort 2 (at least 10 of the first subjects age 5 to 11 years) and Cohort 3 (subjects age 12-17 years) after the Cohort 1 PK analysis is completed.
- **Cohort 2**: At least 10 of the first subjects aged 5 to 11 years will be randomised in a 5:1 ratio to belimumab (10 mg/kg confirmed or adjusted dose) or placebo for 48 weeks on a background of standard of care. After all subjects in Cohort 2 have received at least 8 weeks of treatment, safety and PK analysis will be conducted. Administration of study agent in Cohort 2 will continue while safety monitoring and PK analysis progresses, but no additional subjects ages 5-11 years will enrol in the study until the safety and PK analysis is completed. If the belimumab dose is adjusted, the Cohort 2 subjects age 5 to 11 years will continue with the dose-adjusted blinded treatment.
- **Cohort 3**: This cohort will consist of at least 48 subjects aged 5 to 17 years old. These subjects will be randomised in a 1:1 ratio to belimumab (10 mg/kg confirmed or adjusted dose) or placebo on a background of standard of care for 52 weeks. Randomization will be stratified by age group and screening SELENA SLEDAI scores (6-12 vs. $\geq$ 13). Subjects aged 12 to 17 years will begin enrolment after Cohort 1 enrolment PK analysis is completed. Subjects aged 5 years to 11 years will begin enrolment after Cohort 2 safety and PK analysis is completed.

Younger children (5-11 years old) will not be randomized to treatment in Cohort 3 until reviews of Cohort 1 and 2 have taken place, but they may be identified for potential inclusion at any time.

Two separate dose assessment meetings will be held in which members of the GSK Safety Review Team (SRT) will review the safety, tolerability and preliminary PK data obtained from Cohorts 1 and Cohorts 2, respectively. The objective of these meetings will be to either confirm the initial dose or to adjust the dose if a substantial difference in exposure is observed compared to adult Phase 3 PK data based on 10 mg/kg dosing. An assessment of any safety signal may also factor into the decision to confirm or adjust the initial dose. The SRT will review the available blinded safety, key biomarker and PK data and a recommendation by consensus will be made regarding dose confirmation or adjustment and the initiation of the subsequent cohorts. The PK analysis and recommendation-regarding dose confirmation or adjustment will be reviewed by the IDMC. Final decisions will be made by the sponsor considering the recommendation from the IDMC and independent pharmacokineticist. The Study Team will remain blinded to treatment during these dose assessments. Decisions regarding dose confirmation or adjustment will be summarized and distributed to study team members, investigators, the IDMC, and IRBs, and/or regulatory authorities according to local regulations.

A target enrolment of at least 70 subjects will be randomized. In Cohorts 1 and 2, subjects will be randomized in a 5:1 ratio (belimumab:placebo), and the remaining subjects (at least 48) in Cohort 3 will be randomized in a 1:1 allocation ratio. Therefore, at least 42 subjects will be randomized to belimumab and 28 to placebo. The study will also enrol at least 14 subjects who are younger than 13 years of age. Enrollment will ensure that at least 50% of the randomised subjects will have presented with SELENA SLEDAI ≥ 8 at screening.

Subjects may withdraw or discontinue from the study at any time for any reason. All subjects withdrawing from Part A of the study will return for an exit visit 4 weeks after their last dose of study agent, and then continue in the safety follow-up period (Part C) where safety evaluations and limited disease activity assessments will be performed for up to 10 years from the first administration of study agent or open label belimumab. Subjects choosing not to continue in Part A or participate in Part B and do not wish to be monitored in the safety follow-up period (Part C), will return for a safety follow-up visit approximately 8 weeks following their last dose of study agent. Additional withdrawal criteria are presented in Section 4.4.

Subjects completing the randomized, double-blind, placebo-controlled phase of the study may continue to Part B of the study, the open label safety follow up and receive monthly belimumab treatment.

The study sponsor will remain blinded to subjects' treatment until all data from the Part A of the study are locked and the data are unblinded. Clinical sites will remain blinded until after the results of Part A are publicly disclosed.

Part B: Open Label Belimumab Continuation

Subjects who complete 48 weeks of belimumab or placebo on a background of standard of care and the Week 52 assessments, regardless of treatment assignment, may progress to Part B of the study, the open-label belimumab continuation phase. In this phase, all subjects will receive belimumab (10 mg/kg or adjusted dose) at monthly infusion visits.

The Week 52 administration will be considered the 1st administration of the open-label continuation phase. Safety will be assessed at each monthly visit, and disease activity assessments will be performed every 6 months. Subjects will continue in Part B of the study for up to 10 years from the first administration of belimumab. However, the study will conclude earlier if all subjects continuing belimumab treatment have received at least 5 years of treatment with belimumab (Part B or a combination of Part A and Part B) and if there are 15 or fewer subjects continuing to receive belimumab in the study (See Section 5.7.1 Study Conclusion). Any subject who withdraws during the continuation phase will return for an exit visit at 4 weeks after their last dose and proceed to Part C**,** safety follow-up phase. Subjects choosing to discontinue from the study and not be monitored in the safety follow-up period (Part C), will return for a follow-up visit approximately 8 weeks following their last dose of open label belimumab.

Part C: Safety Follow-up Phase

Part C will include any subject who discontinues study agent from Part A or open label belimumab from Part B. These subjects will return for safety follow up visits in Part C monthly for 3 months and then annually after their last dose of study agent or open label belimumab. In Part C safety evaluations and limited disease activity assessments will be performed for up to 10 years from the first administration of study agent or open label belimumab. However, the study will conclude earlier if all subjects continuing belimumab treatment have received at least 5 years of treatment with belimumab (Part B or a combination of Part A and Part B) and if there are 15 or fewer subjects continuing to receive belimumab in the study (See Section 5.7.1 Study Conclusion). For subjects that withdraw from the study 8 weeks or less from the last administration of IV belimumab, an 8 Week Follow-up Visit (and a 16 Week Follow-up Visit post administration for female subjects of child-bearing potential) must be performed and recorded in the IVR system and the eCRF. An Exit Visit may be conducted by phone for any subject who withdraws completely from the study while in Part C.

Independent Data Monitoring Committee (IDMC)

An IDMC comprised of experts in paediatric rheumatology, paediatric pharmacology, paediatric ethics and a statistician, none of whom are affiliated with the sponsor, will be organized to monitor the study as it progresses. A charter will be developed to govern the activities of this committee. The IDMC will conduct its first safety and PK data review after the first cohort has been treated through the Week 8 (Day 56) visit, or within 4 months of the treatment of the first subject enrolled in Cohort 1, whichever is earlier. If the earlier time point of within 4 months of the first subject enrolled is met, the IDMC will review the available safety data only. The IDMC will continue to meet, at a minimum, approximately every 6 months from the first meeting until the last subject concludes Part A, the randomized treatment period of the study. After each meeting, the IDMC will generate a report of data reviewed and if applicable, recommendations made. Investigators will be notified of the outcome of each IDMC meeting. Notification of local EC/IRB of the IDMC review outcomes will be at the discretion and requirements of local regulations.

Study Endpoints/Assessments for Part A

The primary efficacy endpoint is SLE Responder Index (SRI) **response rate at Week 52 which is defined as:**

- ≥4 point reduction from baseline in SELENA SLEDAI score,

AND

- No worsening (increase of < 0.30 points from baseline) in Physician's Global Assessment (PGA),

AND

- No new BILAG A organ domain score or 2 new BILAG B organ domain scores compared with baseline.

Major Secondary endpoints:

1. Proportion of subjects meeting PRINTO/ACR Juvenile SLE Response Evaluation criteria for improvement in juvenile SLE using two different PRINTO/ACR Juvenile SLE Response Evaluation definitions of improvement
   a. At least 50% improvement in any 2 of 5 endpoints below and no more than 1 of the remaining worsening by more than 30%
   b. At least 30% improvement in 3 of 5 endpoints below and no more than 1 of the remaining worsening more than 30%.
      I. Percent change in Parent's Global Assessment (ParentGA) at Week 52.
      II. Percent change in Physician's Global Assessment (PGA) at Week 52.
      III. Percent change in SELENA SLEDAI score at Week 52.
      IV. Change in PedsQL physical functioning domain at Week 52
      V. Percent change in 24 hour proteinuria at Week 52 (g/24hour equivalent by spot urine protein to creatinine ratio).
2. Proportion of subjects with a sustained SRI response
3. Proportion of subjects with a sustained ParentGA response
4. Safety of belimumab
5. PK comparison with Adult PK
6. PedsQL Multidimensional Fatigue Scale

For Parts B and C, endpoints will include assessment of safety and disease activity as detailed in the Time and Events Table (Table 2).

1. INTRODUCTION

1.1. Disease Background

Systemic lupus erythematosus (SLE) is a chronic autoimmune disorder characterized by autoantibody production and abnormal B lymphocyte function [Pisetsky, 2001]. Due to the heterogeneity of SLE, its diagnosis may be difficult to make. Regardless of age of onset or the time of diagnosis, SLE subjects share many immunogenetic and serologic similarities [Barron, 1993]. There are, however, several important laboratory and clinical features associated with childhood-onset SLE that are different from adult-onset SLE [Brunner, 2008; Costallat, 1994; Tucker, 1995].

Like other rheumatic diseases of childhood, SLE is not identical to that of adults [Lehman, 2007]. Compared with adult SLE, children with SLE have more active disease both at the time of diagnosis and over time. SLE in children is associated with more rapid accrual of damage, and has a higher degree of morbidity compared with SLE in adult populations [Brunner, 2008; Tucker, 2008]. Several studies suggest that glomerulonephritis is more prevalent in childhood-onset than adult-onset SLE [King, 1977; Barron, 1993; Fish, 1977]. In addition, adolescent-onset SLE subjects have a significantly higher occurrence of neurological involvement at the time of diagnosis [Tucker, 2008].

Age of Onset and Incidence

In most adult SLE subjects, symptoms appear on average between 29 and 32 years of age [Jimenez, 2003, Cevera, 1993]. For childhood SLE there is no strict definition with regard to onset. The onset of SLE is almost never seen before the age of 5 [Petty, 2005], with only very rare and anecdotal cases. A recent case series from France in children which looked at the clinical and laboratory manifestations of the disease at onset, showed the mean subject age at onset of childhood SLE to be 11.5 years. [Bader Meunier, 2005].

In a recent review [Pluchinotta, 2007], 13 subjects were identified with signs and symptoms of infantile lupus' (≤2 years old). This infantile lupus was likely caused by transfer of maternal antibodies from mothers with SLE. A recent study based on a chart review of SLE subjects at a Rheumatology Unit of the Department of Pediatrics at an Italian hospital [Pluchinotta, 2007], identified 2 subjects diagnosed under the age of 2 years, 11 subjects aged 2 to 9 years and 29 subjects aged 10 to 15 years at diagnosis. A cross-sectional survey of a sample of 90 pediatric clinics in France for SLE in children up to the age of 16 found a total of 155 cases, with a median age at onset of 12 years, the youngest subject diagnosed at 18 months and 2 further children diagnosed under the age of 5 years [Bader Meunier, 2005]. A chart review at the Pediatric Rheumatology Division of a New York Hospital identified 3 SLE subjects diagnosed under the age of 5 years, 20 subjects aged 5 to 9 years and 82 subjects aged 10 to 19 years [Lehman, 1989]. A study in Israel [Uziel, 2007] ascertained all SLE cases with disease onset prior to the age of 18 years in the country. A total of 102 children were identified with 81% females. The mean age at diagnosis was 13 years, with 16 children (12 females) being younger than age 11 at diagnosis; the youngest being 7. A study in a children's hospital in Toronto [Brunner, 2002] ascertained all childhood-onset SLE over an 8-year period and identified

a total of 66 subjects, with an average age at onset of 13 years, the youngest being 4 years old. A further study investigated childhood SLE cases in three tertiary care centers in Italy and Brazil [Bandeira, 2006] and identified 57 subjects with disease onset prior to the age of 18 years. The mean age at diagnosis was 11 with the youngest age at 6 years.

In the PRINTO series containing 557 from 40 different countries the mean age at onset was 12±2.9 years with 268 with age at onset < 12 years and 55 < 8 years. [Ruperto, 2005, Ruperto, 2006]

The true incidence and prevalence of childhood SLE remain unknown [Singsen, 1998]. The reported estimates in the literature are based on only a very small number of cases and hence are subject to considerable variability and uncertainty. As a very rough estimate, the incidence for the age groups that approximate the group 12-18 years appears to be in the range of 1-4/100,000/year for both sexes combined, with an even considerably lower incidence in subjects < 12 years. The SLE prevalence could be estimated to be in the range of about 2-6/100,000 for children ≥ 12 years, again with a drastically lower prevalence for children below that age.

A study in the population of Iceland [Gudmundsson, 1990] over a 10-year period found no cases in children aged ≤9 years and 3 cases (incidence 3/100,000) in females and 1 case (1/100,000) in males aged 10-14 years. A study in the arctic region of Norway [Nossent, 2001] found 4 cases aged ≤ 15 years (incidence 0.5/100,000) over an 8-year period. A Spanish study [Lopez, 2003] identified 1 female case (prevalence 1.9/100,000) in the age group 0-14 years. A study using a GP records database in the UK [Nightingale, 2007] identified 1 male case (prevalence 1.3/100,000) in the age group 0-9 years and 11 female cases (10.6/100,000) and 2 male cases (1.8/100,000) in the 10-19 age group. A second study using the same database [Somers, 2007] estimated an incidence of < 0.5/100,000 for the ages 0-9 and 0.5-1/100,000 for the ages 10-14 years. Other studies reported 6 cases under the age of 18 in Northern Ireland, UK [Gourley, 1997] and 2 cases under 19 years of age in Vilnius, Lithuania [Dadoniene, 2006]. These cases were likely to have been older than 11 years at diagnosis. A study in Nottingham, UK [Hopkinson, 1993] found no cases for the ages 0-9 years and 1 female case (incidence 2.5/100,000) in the age group 10-19 over a 2-year period. Two US studies [Somers, 2007; Naleway, 2005] investigated SLE in those younger than 20 years old with results indicating that SLE is unlikely to occur under the age of 12 years.

The Iceland study [Gudmundsson, 1990] estimated an incidence of 4/100,000/year for the age group 15-19, while the UK GPRD study [Somers, 2007] shows an incidence of 2/100,000/year for the same age group. The UK Nottingham study estimates an incidence of 2.5/100,000/year for females aged 10-19 years. The prevalence was reported as 12.9/100,000 for females for the ages 0-19 in the Lithuania study [Dadoniene, 2006], 10.6 and 1.8/100,000 for females and males aged 10-19 respectively in the UK GPRD study [Nightingale, 2007] and 20.4 and 0/100,000 in the US Wisconsin study [Naleway, 2005] for the same age-gender groups.

Taken together all of this information, it appears that the onset of SLE in children generally appears at an age of 10 to 11 years, with only rare cases in children younger than that, but virtually almost never below an age of 5.

Disease Diagnosis

The diagnosis of SLE in the pediatric population is hampered by the same issues as the diagnosis of SLE in adults, i.e. there is still considerable uncertainty and ongoing discussion to define general and globally accepted diagnostic criteria for SLE in both adults and children. For adult clinical trials, the ACR Criteria for the Classification of Systemic Lupus Erythematosus are often used, which suggest that for the purpose of identifying subjects in clinical studies, a person shall be said to have SLE if any 4 or more of the 11 criteria are present, serially or simultaneously, during any interval of observation [Tan, 1982; Hochberg, 1997].

Due to the heterogeneity of SLE, its diagnosis may be difficult to make in a general pediatric or community setting. However, regardless of age of onset or of the time of diagnosis, SLE subjects share many immunogenetic and serologic similarities [Barron, 1993]. There are, however, several important laboratory and clinical features associated with childhood-onset SLE (i.e., before age 16) that are different from adult-onset SLE [Brunner, 2008; Costallat, 1994; Tucker, 1995].

Serologically, anti-dsDNA, anti-Sm and anti-RNP antibodies and a low C3 are more prevalent in juvenile-onset than in adult-onset SLE [Tucker, 1995; Press, 1996; Reichlin, 1999]. However, complement, ANA, anti-dsDNA, aCL, and lupus anti-coagulant antibodies are not significantly different in children of any age group.

Treatment

The clinical evidence of effective treatment of childhood rheumatic diseases including SLE is generally based on anecdotal reports [Lehman, 1997]. Generally, children are treated with the same agents used in the adult population. Standard therapies for SLE include corticosteroids (the mainstay of therapy), anti-malarial agents (e.g., hydroxychloroquine), non-steroidal anti-inflammatory drugs (NSAIDs), cytotoxic agents like cyclophosphamide, and immunosuppressive/immunomodulatory agents (e.g., azathioprine, cyclosporine, mycophenolate mofetil, methotrexate, leflunomide, thalidomide, 6-mercaptopurine) [Reveille, 2001; Petri, 2001; Ruiz-Irastorza, 2001; Chatham, 2001; Wallace, 2002; Brocard, 2005; Houssiau, 2004]. In the US, corticosteroids, hydroxychloroquine, and aspirin have been approved for SLE; no drug has been approved for the treatment of lupus in over 50 years. Likewise, no drug has yet been approved across the entirety of the EU for the treatment of SLE. However, prednisolone, azathioprine and hydroxychloroquine have been authorised to treat SLE in various EU member states.

All these therapies, whether approved or used off-label, can be associated with significant toxicity: Long-term use of high-dose corticosteroids can cause significant morbidity including osteoporosis, osteonecrosis, metabolic disorders (including exacerbation of diabetes), increased infection risk, edema, weight gain and hyperlipidemia [Chatham, 2001]. Cytotoxic agents like cyclophosphamide or azathioprine are immunosuppressive, resulting in increased risk of serious infections and certain cancers. Headache, dizziness, loss of appetite, upset stomach, diarrhea, stomach pain, vomiting and skin rash which are the side effects of hydroxychloroquine are not life-threatening, nevertheless they are responsible for the decreased quality of life of SLE subjects which

is of importance as all SLE treatments are chronic, since there is currently no cure for SLE.

Supporting information on treatment of the pediatric population from clinical trials is also rather limited as most clinical trials for SLE are conducted in adults. Small case series involving B-cell depletion therapy such as rituximab in children with SLE have been published [Podolskaya, 2008; Marks, 2007; El-Hallak, 2007]. To date, the use of rituximab in SLE both in adult and pediatrics populations remains off-label. Clinical trials in juvenile SLE are being undertaken currently; yet performing clinical trials in juvenile SLE remains a challenge because of the small number of eligible subjects and the heterogeneity of the disease [Ruperto, 2004].

Study Drug Background

Belimumab (also known as LymphoStat-B™; BENLYSTA™) is a B-lymphocyte stimulator (BLyS)-specific inhibitor that blocks the binding of soluble BLyS, a B-cell survival factor, to its receptors on B cells. Belimumab does not bind B cells directly, but by binding BLyS, belimumab inhibits the survival of B cells, including autoreactive B cells, and reduces the differentiation of B cells into immunoglobulin-producing plasma cells. Belimumab reduces, but does not deplete, B-cells. The lack of severe depletion of B-cells across all subsets supports the hypothesis that belimumab modulates B-cells and has the potential to preserve cells that respond to foreign or abnormal self antigens.

Belimumab administered IV at a dose of 1 or 10 mg/kg compared with placebo was studied in two Phase 3 studies in adult subjects (N= 1,684) with active (SELENA SLEDAI ≥6), autoantibody-positive (ANA or anti-dsDNA) SLE who were receiving standard therapy (including steroids, antimalarials, and immunosuppressants). In both studies, belimumab 10 mg/kg demonstrated superior efficacy as measured by response rate at Week 52 using the SLE Responder Index (SRI), the primary efficacy endpoint in each trial. Evidence of benefit in other clinical measures such as reductions in disease activity as measured by SELENA SLEDAI, severe flare, and reduced steroid use was also observed. Treatment with belimumab in adult subjects with SLE is also associated with reduction in autoantibodies and increases (normalization) of complement levels.

Treatment with belimumab plus standard of care was generally well tolerated, with rates of adverse events (AEs), severe AEs, serious AEs, AEs leading to discontinuation, and serious/severe infections generally comparable to the rates observed in the placebo plus standard of care group. Mortality rates in the controlled clinical trials were low, although numerically higher in the belimumab groups: 0.4% and 0.8% in the placebo and belimumab groups, respectively. Causes of death were as expected in an SLE population with active disease receiving a wide range of standard therapies, such as steroids and immunosuppressants, and included infection, cardiovascular disease. Serious infections were observed in 5.2% and 6.0% of subjects receiving placebo and belimumab, respectively. The rate of malignancy (excluding non-melanoma skin cancer) was the same between the placebo and belimumab groups at 0.4%; however as with other immunomodulating agents, the mechanism of action of belimumab could increase the risk for the development of malignancies. Hypersensitivity and infusion reactions were observed. Anaphylaxis was also observed, though rare (<1%). Depression-related events,

common in subjects with SLE, were observed more frequently with belimumab than with placebo; it is unknown if belimumab treatment is associated with an increased risk for these events. The most commonly-reported adverse reactions, occurring in ≥ 5% of subjects in clinical trials were nausea, diarrhea, pyrexia, nasopharyngitis, bronchitis, insomnia, pain in extremity, depression, migraine, and pharyngitis.

Experience from open-label, long-term continuation trials of belimumab in SLE subjects suggests that prolonged treatment with belimumab remains generally well tolerated, with no apparent increase in the incidence rate of AEs or serious adverse events (SAEs) over time, including important events such as infections and malignancies. The prevalence rate of AEs and SAEs has also remained relatively stable over time. Long term belimumab treatment through 6 years appears to provide sustained improvement in SLE disease activity.

In conjunction with functional differentiation of immune cells and the maturation of lymph node architecture from infancy through early childhood and adolescence, there is a progressive increase in general cytokine mRNA and protein expression with age [Härtel, 2005]. The bone marrow in children has more hemopoetic cellular content and less fat than adults. Within the bone marrow, transitional and naïve B-cells undergo proliferation and differentiation to become immunoglobulin secreting B-cells and long-lived plasma cells. Bone marrow multipotent mesenchymal stromal cells enhance naïve B-cell proliferation and differentiation in a similar manner in both normal healthy donors and pediatric SLE subjects [Timonen, 2003; Traggiai, 2008]. The effect of belimumab upon B-cell survival would be expected to be similar between adults and children, although the sensitivity and extent of apoptosis is unknown. The implications for immune system function and potential long-term effects are unknown for the pediatric population. In general, the action of belimumab is expected to be the same in the adult and in the pediatric SLE population.

Further information on the safety and efficacy of belimumab is provided in the Investigator Brochure (IB).

1.2. Rationale

Recent and past studies consistently show that adult and pediatric SLE subjects have increased serum BLyS levels [Hong, 2009; Cheema, 2001; Petri, 2008; Wang, 2008]. In SLE, the elevation of BLyS may contribute to the persistence of B-cell subsets that produce pathogenic autoantibodies or promote inflammation that would otherwise be subject to down regulation. Thus a therapeutic strategy that involves an antagonist to BLyS may have therapeutic benefit in SLE. In addition, this therapeutic strategy of utilising a BLyS specific inhibitor, belimumab, proved successful with the positive results from two pivotal adult lupus studies. In general, pediatric SLE subjects have more severe disease and thus higher disease activity index on average than adults. Adult SLE subjects who presented with more active disease performed better with belimumab. It is theorized based on this, that belimumab will have a beneficial effect in the pediatric SLE population.

Brunner HI, *et al. Ann Rheum Dis* 2020; 79:1340–1348. doi: 10.1136/annrheumdis-2020-217101

The Phase III clinical studies of belimumab demonstrated positive benefit/risk in the adult population. Marketing authorisation of belimumab for adults was granted in the EU on 13 July 2011 and belimumab received marketing approval from the FDA on 09 March 2011. Belimumab is currently approved in the United States, all European Union Member States, as well as in over 20 countries. As a result of demonstration of positive benefit risk in the adult SLE population it is reasonable to expect a similar safety and efficacy profile in juvenile SLE. Additionally, no authorised pediatric medicinal products for SLE exist on the market. Additionally, this trial fulfils the post-approval regulatory requirements to conduct a pediatric study.

The purpose of this study is to evaluate the safety, efficacy, pharmacokinetics, and quality of life of belimumab administered intravenously (IV) compared to placebo in pediatric subjects with systemic lupus erythematosus (SLE) receiving background standard therapy.

1.2.1. Dose Rationale

The belimumab 10 mg/kg dose was selected based on the safety, efficacy and pharamacokinetic data from the two pivotal Phase 3 studies in adults with SLE. This dose will be confirmed or adjusted based on PK and safety assessments for each age group as described in Section 3.1.

In the pivotal Phase 3 studies of belimumab in adult subjects with SLE, two doses were studied, 1 mg/kg and 10 mg/kg. Although both belimumab 1mg/kg and 10mg/kg doses were active, the 10mg/kg dose was proposed for the pediatric trial because only the 10mg/kg dose achieved the primary efficacy endpoint in both Phase 3 adult studies. Moreover, in the Phase 3 adult studies several clinical measures of improvement including the primary efficacy endpoint at Week 52 (including all sensitivity analyses), SELENA SLEDAI reductions, severe flare risk reduction, and complement normalization generally favored the 10 mg/kg dose. While steroid reductions tended to favor 1 mg/kg belimumab, fewer subjects receiving 10 mg/kg belimumab required increases in steroids over time. Additionally, there appeared to be a greater dose response in subjects with high disease and serological activity than in subjects with lower activity. Since pediatric subjects typically present with higher disease activity, the additional benefit of 10 mg/kg versus 1 mg/kg dosing is expected to be larger in children compared to the general adult population. There was no apparent dose-response in the safety profile of belimumab in adult subjects, with both doses being generally well-tolerated, further supporting the selection of 10 mg/kg belimumab as the recommended dose in combination with standard therapies. The 10 mg/kg dose is approved in the United States, all European Union Member States, as well as in over 20 countries for the treatment of adult subjects with active, autoantibody-positive SLE.

BLyS-mediated clearance of belimumab did not appear to play a substantial role in the pharmacokinetics for 1 to 20 mg/kg dosing in adult SLE subjects, likely due to the large molar excess of serum belimumab relative to circulating BLyS levels even at trough concentrations for the lowest dose. Since circulating BLyS level were found to be similar in pediatric compared to adult subjects, there is no reason to change the adult dose in children based on differential target expression.

Adult Phase 3 PK data based on weight normalized dosing for subjects with similar BMI values show little dependency of exposure on weight compared to the variability originating from other factors. This lends further support to use the adult weight normalized dose of 10 mg/kg in the initial juvenile treatment group

Based on this assessment subjects 12 years and older are expected to have similar belimumab exposure than adults with weight normalized dosing. For younger subjects somewhat lower exposures are expected with 10 mg/kg dosing, due to the body size dependencies observed for adult PK data, the expected lower average BMI in the pediatric group and possibly other developmental differences between adults and juveniles affecting therapeutic antibody clearance.

Previous clinical experience for other IgG monoclonal antibodies has shown that weight normalized dosing resulted in similar exposures in juveniles compared to adults and depending on disease and tested weight range similar or lower exposures in younger children [Stiehm, 2000, Kovarik, 2002; Baldassano, 2003; Pescovitz, 2008].

As described in Section 3.1, PK data in the initial subjects of both staggered age groups will be monitored to separately assess any substantial dependencies of exposure on body size or age and whether these warrant any dose changes to achieve a similar exposure as for adults with 10 mg/kg dosing in any of the two age groups.

2. OBJECTIVES

- Evaluate the safety and tolerability of belimumab in the pediatric SLE population
- Evaluate the pharmacokinetics of belimumab in the pediatric SLE population.
- Evaluate the efficacy of belimumab in the pediatric SLE population
- Evaluate the effects of belimumab on the quality of life in the pediatric SLE population.

3. INVESTIGATIONAL PLAN

3.1. Study Design

This is a multi-center study to evaluate the safety, efficacy and pharmacokinetics of belimumab plus background standard therapy in at least 70 pediatric subjects 5 years to 17 years of age with active systemic lupus erythematosus (SLE). The study will consist of three separate phases:

- Randomized, placebo-controlled, double-blind 52-week treatment phase Part A
- Long term belimumab open label safety follow up for any subject who completes Part A (Part B)
- Long term safety follow up phase for subjects who withdraw from Part A or Part B at any time (Part C)

Part A is a randomized, placebo-controlled, double-blind study to evaluate the efficacy, safety, and pharmacokinetics of 10 mg/kg belimumab intravenous (IV) in pediatric subjects with active SLE (SELENA SLEDAI score ≥6). In this study, at least 70 subjects will be randomized in three cohorts with Cohort 3 stratified by age (5-11 years vs. 12-17 years) and screening SELENA SLEDAI scores (6-12 vs. ≥ 13). Cohorts 1 and 2 will be randomized in a 5:1 ratio, and Cohort 3 subjects will be randomized in a 1:1 ratio to receive belimumab or placebo for 48 weeks on a background of standard of care. Cohort 1 will consist of the first 12 subjects, age 12 to 17 years. No further enrolment of the study will proceed until after the PK of this cohort is evaluated. Once the PK and safety profile of Cohort 1 is evaluated and any potential dose adjustments are determined as a result of this analysis, additional subjects 12-17 years of age will then be enrolled in Cohort 3. In addition, at this point, Cohort 2 will enrol at least 10 subjects 5-11 years of age at the dose determined from the PK analysis of Cohort 1. Once the PK and safety profile of Cohort 2 is evaluated and any potential dose adjustments are determined as a result of this analysis, additional subjects 5-11 years of age will then be enrolled in Cohort 3.

Belimumab will be infused over a minimum of 1 hour on Days 0, 14, 28, and then every 28 days through the Week 48 (Day 336) visit. All subjects will continue to receive their standard SLE therapy with progressive restrictions on the changes that are permitted throughout the 52-week randomized period. Enrolment will be staggered to allow PK analysis of the first 2 age cohorts (Cohorts 1 and 2).

Safety monitoring and PK analysis from the initial 12 subjects from Cohort 1 will be used to confirm or adjust the belimumab dose for the remaining subjects enrolling in Cohort 2 and 3.

- **Cohort 1:** The first 12 subjects aged 12 to 17 years of age will be randomized in a 5:1 ratio to belimumab 10 mg/kg (n=10) or placebo (n=2) on a background of standard of care for 48 weeks. After all 12 subjects in Cohort 1 have received at least 8 weeks of treatment, safety and PK analysis will be conducted. Cohort 1 subjects will continue in the treatment period while the Study Team progresses the PK analysis, but no other subjects will enrol until the PK analysis is

completed. If the belimumab dose is adjusted because of the PK analysis, the Cohort 1 subjects will continue the study with the adjusted dose. Enrolment will be initiated for Cohort 2 (at least the first 10 subjects age 5 to 11 years) and Cohort 3 (subjects age 12-17 years) after the Cohort 1 PK analysis is completed.

- **Cohort 2**: At least the first 10 subjects aged 5 to 11 years will be randomized in a 5: 1 ratio to belimumab (10 mg/kg confirmed or adjusted dose) or placebo for 48 weeks on a background of standard of care. After all subjects in Cohort 2 have received at least 8 weeks of treatment, safety and PK analysis will be conducted. Administration of study agent in Cohort 2 will continue while safety monitoring and PK analysis progresses but no additional subjects ages 5-11 will be enrolled into the study until after the safety and PK analysis have been completed. If the belimumab dose is adjusted, the Cohort 2 subjects aged 5 to 11 years will continue with the dose-adjusted blinded treatment.
- **Cohort 3**: This cohort will consist of at least 48 subjects aged 5 to 17 years old. These subjects will be randomized in a 1:1 ratio to belimumab (10 mg/kg confirmed or adjusted dose) or placebo on a background of standard of care for 52 weeks. Randomization will be stratified by age group and screening SELENA SLEDAI scores (6-12 vs. ≥ 13). Subjects aged 12 to 17 years will begin enrolment after Cohort 1 enrolment PK analysis is completed. Subjects aged 5 years to 11 years will begin enrolment after Cohort 2 safety and PK analysis is completed.

Younger children (5-11 years old) will not be randomized to treatment until review of Cohorts 1 and 2 have taken place, but they may be identified for potential inclusion at any time.

Two separate dose assessment meetings will be held in which members of the GSK Safety Review Team (SRT) will review the safety, tolerability and preliminary PK data obtained from Cohorts 1 and Cohorts 2, respectively. The objective of these meetings will be to either confirm the initial dose or to adjust the dose if a substantial difference in exposure is observed compared to adult Phase 3 PK data based on 10 mg/kg dosing. An assessment of any safety signal may also factor into the decision to confirm or adjust the initial dose. The SRT will review the available blinded safety, key biomarker and PK data and a recommendation by consensus will be made regarding dose confirmation or adjustment and the initiation of the subsequent cohorts. The PK analysis and recommendation regarding dose confirmation or adjustment will be reviewed by the IDMC. Final decisions will be made by the sponsor considering the recommendation from the IDMC and independent pharmacokineticist. The Study Team will remain blinded to treatment during these dose assessments. Decisions regarding dose confirmation or adjustment will be summarized and distributed to study team members, investigators, the IDMC and IRBs and/or regulatory authorities according to local regulations.

A target enrolment of at least 70 subjects will be randomized. In Cohorts 1 and 2, subjects will be randomized in a 5:1 ratio (belimumab:placebo), and the remaining

subjects (at least 48) in Cohort 3 will be randomized in a 1:1 allocation ratio. Therefore, at least 42 subjects will be randomized to belimumab and 28 to placebo. The study will also enrol at least 14 subjects who are younger than 13 years of age. Enrollment will ensure that at least 50% of the randomised subjects will have presented with SELENA SLEDAI ≥ 8 at screening.

Subjects may withdraw or discontinue from the study at any time for any reason. All subjects withdrawing from Part A of the study will return for an exit visit 4 weeks after their last dose of study agent, and then continue in the safety follow-up period (Part C) where safety evaluations and limited disease activity assessments will be performed for up to 10 years from the first administration of study agent or open label belimumab. Subjects choosing not to continue in Part A or participate in Part B and do not wish to be monitored in the safety follow-up period (Part C), will return for a safety follow-up visit approximately 8 weeks following their last dose of study agent. Additional withdrawal criteria are presented in Section 4.4.

Subjects completing the randomized, double-blind, placebo-controlled phase of the study may continue to Part B of the study, the open label safety follow up and receive monthly belimumab treatment.

The study sponsor will remain blinded to subjects' treatment until all data from the Part A of the study are locked and the data are unblinded. Clinical sites will remain blinded until after the results of Part A are publicly disclosed.

Part B: Open Label Belimumab Continuation

Subjects who complete 48 weeks of belimumab or placebo on a background of standard of care and the Week 52 assessments, regardless of treatment assignment, may progress to Part B of the study, the open-label belimumab continuation phase. In this phase, all subjects will receive belimumab (10 mg/kg or adjusted dose) at monthly infusion visits. The Week 52 administration will be considered the 1st administration of the open-label continuation phase. Safety will be assessed at each monthly visit, and disease activity assessments will be performed every 6 months. Subjects will continue in Part B of the study for up to 10 years from the first administration of belimumab. However, the study may conclude earlier if all subjects continuing belimumab treatment have received at least 5 years of treatment with belimumab (Part B or a combination of Part A and Part B) and if there are 15 or fewer subjects continuing to receive belimumab in the study (See Section 5.7.1 Study Conclusion). Any subject who withdraws during the continuation phase will return for an exit visit at 4 weeks after their last dose and proceed to Part C, safety follow-up phase. Subjects choosing to discontinue from the study and not be monitored in the safety follow-up period (Part C) will return for a follow-up visit approximately 8 weeks following their last dose of open label belimumab.

Part C Safety Follow-up Phase

Part C will include any subject who discontinues study agent from Part A or open label belimumab from Part B. These subjects will return for safety follow up visits in Part C monthly for 3 months and then annually after their last dose of study agent or open label belimumab. In Part C safety evaluations and limited disease activity assessments will be

performed for up to 10 years from the first administration of study agent or open label belimumab. However, the study may conclude earlier if all subjects continuing belimumab treatment have received at least 5 years of treatment with belimumab (Part B or a combination of Part A and Part B) and if there are 15 or fewer subjects continuing to receive belimumab in the study (See Section 5.7.1 Study Conclusion). For subjects that withdraw from the study 8 weeks or less from the last administration of IV belimumab, an 8 Week Follow-up Visit (and a 16 Week Follow-up Visit post administration for female subjects of child-bearing potential) must be performed and recorded in the IVR system and the eCRF. An Exit Visit may be conducted by phone for any subject who withdraws completely from the study while in Part C.

Independent Data Monitoring Committee (IDMC)

An IDMC comprised of experts in paediatric rheumatology, paediatric pharmacology, paediatric ethics and a statistician, none of whom are affiliated with the sponsor, will be organized to monitor the study as it progresses. A charter will be developed to govern the activities of this committee. The IDMC will conduct its first safety and PK data review after the first cohort has been treated through the Week 8 (Day 56) visit, or within 4 months of the treatment of the first subject enrolled in Cohort 1, whichever is earlier. If the earlier time point of within 4 months of the first subject enrolled is met, the IDMC will review the available safety data only. The IDMC will continue to meet, at a minimum, approximately every 6 months from the first meeting until the last subject concludes Part A, the randomized treatment period of the study. After each semiannual review meeting, the IDMC will generate a report of data reviewed and if applicable, recommendations made. Investigators will be notified of the outcome of each IDMC meeting. Notification of local EC/IRB of the IDMC review outcomes will be at the discretion and requirements of local regulations.

Decisions regarding dose confirmation or adjustment as a result of the planned Cohort 1 and Cohort 2 PK and safety analyses will be summarized and distributed to study team members, investigators, the IDMC, and IRBs and/or regulatory authorities according to local regulations.

Figure 1 BEL114055 Study Schematic

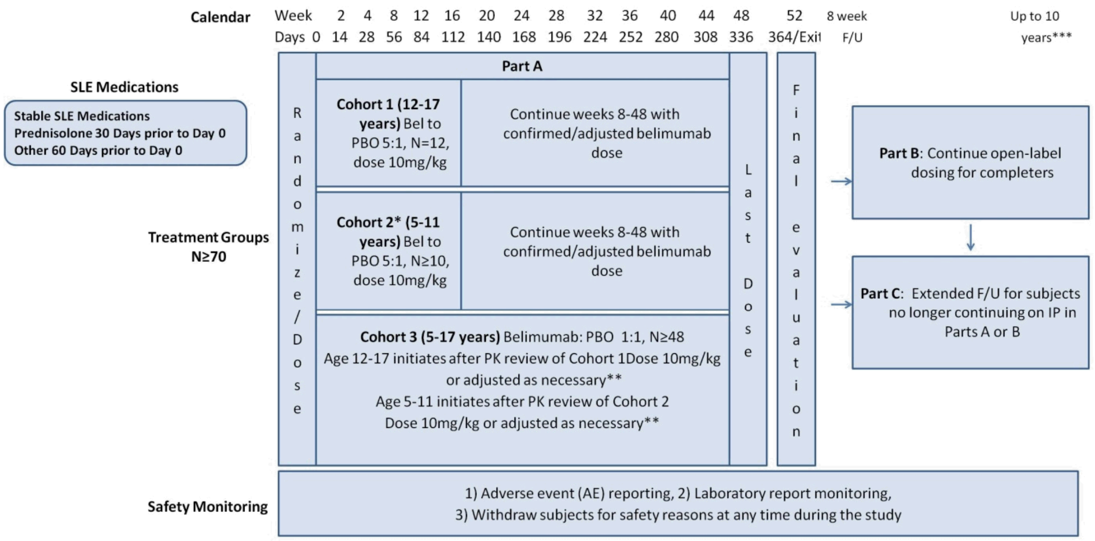

* Initiate Cohort 2 after confirmed/adjusted dose from Cohort 1 PK review
** PK group will remain blinded regardless of outcome
***May conclude earlier if the last subject has completed at least 5 years of treatment w/belimumab and the number of subjects continuing with belimumab is <15.

4. SUBJECT SELECTION AND WITHDRAWAL CRITERIA

4.1. Number of Subjects

A target enrolment of at least 70 subjects to descriptively evaluate the efficacy and safety of belimumab is planned. In the first two cohorts, subjects will be randomized in a 5:1 ratio (belimumab:placebo), and the remaining subjects will be randomized in a 1:1 allocation ratio.

4.2. Inclusion Criteria

Specific information regarding warnings, precautions, contraindications, adverse events, and other pertinent information on the study agent or other study treatment that may impact subject eligibility is provided in the Investigator Brochure (IB).

Deviations from inclusion criteria are not allowed because they can potentially jeopardize the scientific integrity of the study, regulatory acceptability or subject safety. Therefore, adherence to the criteria as specified in the protocol is essential.

Because of the uncertainty of vaccine response in any immunosuppressed population, it is recommended that all vaccinations be up to date as per country specific guidelines prior to entry into this study. Live vaccinations are not to be administered less than 30 days before first dose or while subjects are receiving study agent.

In addition, consideration should be given to administration of certain vaccines, in particular pneumococcus and meningococcus, prior to entry if the country specific vaccine schedule would require them soon after study start.

Brunner HI, *et al. Ann Rheum Dis* 2020; 79:1340–1348. doi: 10.1136/annrheumdis-2020-217101

Subjects eligible for enrolment in the study must meet all of the following criteria:

1. Are 5 to 17 years of age.

2. Have or have had in series, 4 or more of the American College of Rheumatology (ACR) 11 criteria for the classification of SLE

3. Have active SLE disease defined as a SELENA SLEDAI score ≥ 6 at screening.

4. Have unequivocally positive autoantibody test results defined as an ANA titre ≥ 1:80 and/or a positive anti-dsDNA (≥ 30 IU/mL) serum antibody test from 2 independent time points as follows:

   •Positive test results from 2 independent time points within the study screening period. Screening results must be based on the study's central laboratory results.

   OR

   •One positive historical test result and 1 positive test result during the screening period.

   - Historical documentation of a positive test of ANA (e.g., ANA by HEp-2 titre) or anti-dsDNA (e.g., anti-dsDNA by Farr assay) must include the date and type of the test, the name of the testing laboratory, numerical reference range, and a key that explains values provided as positive vs. negative OR negative, equivocal/borderline positive). Only unequivocally positive values as defined in the laboratory's reference range are acceptable; borderline values will not be accepted.

5. Are on a stable SLE treatment regimen.

"Stable treatment at baseline" consists of any of the following medications (alone or in combination) at a fixed dose for a period of at least 30 days prior to Day 0:

- Corticosteroids (prednisone or prednisone equivalent up to 0.5 mg/kg/day):
  - For subjects on SLE combination therapy, their stable steroid dose must be fixed within the range of 0 to 0.5mg/kg/day (prednisone or prednisone equivalent)
  - For subjects whose only SLE treatment is steroids, their stable steroid dose must be fixed within the range of 0.1-0.5mg/kg/day.
  - For those subjects on alternating day doses of steroids, use the average of 2 daily doses to calculate the average daily steroid dose.
- Other immunosuppressive or immunomodulatory agents including methotrexate, azathioprine, leflunomide, mycophenolate (including mycophenolate mofetil, mycophenolate mofetil hydrochloride, and mycophenolate sodium), calcineurin inhibitors (e.g. tacrolimus, cyclosporine), sirolimus, oral cyclophosphamide, 6-mercaptopurine or thalidomide.

- Anti-malarials (e.g. hydroxychloroquine, chloroquine, quinacrine)

- Non-steroidal anti-inflammatory drugs (NSAIDs)

Note:

- Pre-existing SLE medications must be stable for at least 30 days prior to Day 0.
- Corticosteroids may be added as new medications or their doses adjusted only up to 30 days prior to Day 0.
- New SLE therapy other than corticosteroids must not be added within 60 days of Day 0.

6. A sexually active female subject is eligible to enter the study if she is:

- Not pregnant or nursing;
- Of non-childbearing potential. Non-childbearing potential is defined as a pre-menarcheal female who has not yet entered puberty as evidenced by lack of breast development (palpable glandular breast tissue); or a female who has documentation (medical report verification) of a hysterectomy, has both ovaries surgically removed or tubal ligation; or
- Of childbearing potential (i.e., with functional ovaries and no documented impairment of oviductal or uterine function that would cause sterility). This category includes those with oligomenorrhoea [even severe]. These must have a negative serum pregnancy test at screening, and agree to 1 of the following:

Complete abstinence from intercourse from 2 weeks prior to administration of the 1st dose of study agent until 16 weeks after the last dose of study agent; or

Consistent and correct use of 1 of the following acceptable methods of birth control for 1 month prior to the start of the study agent and 16 weeks after the last dose of study agent:

- Implants of levonorgestrel or etonogestrel;
- Ethinyl estradiol/Etonogestrel vaginal ring;
- Injectable progesterone;
- Any intrauterine device (IUD) with a documented failure rate of less than 1% per year;
- Oral contraceptives (either combined or progesterone only);
- Double barrier method: condom and occlusive cap (diaphragm or cervical/vault caps) with spermicidal foam/gel/film/cream/suppository;
- Transdermal contraceptive patch;
- Male partner who is sterile prior to the female subject's entry into the study and is the sole sexual partner for the female subject.

Note: Mycophenolate mofetil (MMF) and other forms of mycophenolate affect the metabolism of oral contraceptives and may reduce their effectiveness. As such, women

receiving mycophenolate who are using oral contraceptives for birth control should employ an additional method (eg, barrier method).

7. Subject signs and dates a written age appropriate assent form (in accordance with applicable regulations) and the parent or legal guardian (or emancipated minor) that has the ability to understand the requirements of the study, provides written informed consent (including consent for the use and disclosure of research-related health information) that the subject will comply with the study protocol procedures (including required study visits).

4.3. Exclusion Criteria

Deviations from exclusion criteria are not allowed because they can potentially jeopardize the scientific integrity of the study, regulatory acceptability or subject safety. Therefore, adherence to the criteria as specified in the protocol is essential.

Subjects meeting any of the following criteria must not be enrolled in the study:

1. Have received treatment with belimumab (BENLYSTA) at any time
2. Have received any of the following within 364 days of Day 0:
   - Treatment with any B-cell targeted therapy (e.g., rituximab, other anti-CD20 agents, anti-CD22 [epratuzumab], anti-CD52 [alemtuzumab], BLyS-receptor fusion protein [BR3], TACI-Fc)
   - Abatacept.
   - A biologic investigational agent
3. Have required 3 or more courses of systemic corticosteroids for concomitant conditions (e.g., asthma, atopic dermatitis) within 364 days of Day 0 (Topical or inhaled steroids are permitted).
4. Have received intravenous (IV) cyclophosphamide within 60 days of Day 0.
5. Have received any of the following within 90 days of Day 0:
   - Anti-TNF therapy (e.g., adalimumab, etanercept, infliximab).
   - Interleukin-1 receptor antagonist (anakinra).
   - Intravenous immunoglobulin (IVIG).
   - Plasmapheresis.
6. Have received any of the following within 60 days of Day 0:
   - A non-biologic investigational agent.
   - Any new immunosuppressive/immunomodulatory agent, anti-malarial, NSAID, (See Inclusion Criteria #5)

Note: New inhaled steroids and new topical immunosuppressive agents (e.g., eye drops, topical creams) are allowed. Any NSAID use for < 1 week is allowed.

- High dose prednisone or equivalent (> 1.5 mg/kg/day) or any steroid injection (intramuscular, intraarticular or intravenous).

7. Have received any of the following within 30 days of Day 0:
   - A live vaccine.
   - A change in dose of a corticosteroid, other immunosuppressive/ immunomodulatory agent, anti-malarial, NSAID, (See Inclusion Criteria #5)
8. Have active central nervous system (CNS) lupus (including seizures, psychosis, organic brain syndrome, cerebrovascular accident [CVA], cerebritis or CNS vasculitis) requiring therapeutic intervention within 60 days of Day 0.
9. Have required renal replacement therapy (e.g. hemodialysis, peritoneal dialysis) within 90 days of Day 0 or be currently on renal replacement therapy
10. Have an estimated glomerular filtration rate (eGFR) as calculated by Schwartz Formula of less than 30 ml/min.
11. Have acute severe nephritis defined as a significant worsening of renal disease (e.g., the presence of urinary sediments and other lab abnormalities) that, in the opinion of the study investigator, may lead to the subject requiring induction therapy with IV cyclophosphamide, MMF or high dose corticosteroids during the first 6 months of the trial.

Note: clinically stable lupus nephritis which can be managed with medications allowed in the study will not exclude subjects from participating in the trial (nor will any maximum level of proteinuria exclude subjects). Clinical assessment and medical management of nephritis will be at the discretion of the study investigator.

12. Have a history of a major organ transplant (e.g., heart, lung, kidney, liver) or hematopoetic stem cell/marrow transplant.

13. Have clinical evidence of significant, unstable or uncontrolled, acute or chronic diseases not due to SLE (i.e., cardiovascular, pulmonary, hematologic, gastrointestinal, hepatic, renal, neurological, malignancy or infectious diseases) which, in the opinion of the principal investigator, could confound the results of the study or put the subject at undue risk.

14. Have a planned surgical procedure or a history of any other medical disease (e.g., cardiopulmonary), laboratory abnormality, or condition (e.g., poor venous access) that, in the opinion of the principal investigator, makes the subject unsuitable for the study.

15. Have a history of malignant neoplasm within the last 5 years.

16. Subjects ≥ 12 years of age who have evidence of serious suicide risk including any history of suicidal behavior in the last 6 months and/or any suicidal ideation of type 4

or 5 on the Columbia Suicide Severity Rating Scale (Refer to Appendix 12) in the last 2 months or who in the investigator's judgment, pose a significant suicide risk.

17. Have a history of a primary immunodeficiency
18. Have an IgA deficiency (IgA level < 10 mg/dL).
19. Have acute or chronic infections requiring management, as follows:
    - Currently on any suppressive therapy for a chronic infection (such as tuberculosis, pneumocystis, cytomegalovirus, herpes simplex virus, herpes zoster and atypical mycobacteria).
    - Hospitalization for treatment of infection within 60 days of Day 0.
    - Use of parenteral (IV or IM) antibiotics (antibacterials, antivirals, anti-fungals, or anti-parasitic agents) for infection within 60 days of Day 0.
20. Have current drug or alcohol abuse or dependence, or a history of drug or alcohol abuse or dependence within 364 days prior to Day 0.
21. Have a historically positive HIV test or test positive at screening for HIV.
22. Hepatitis B: Serologic evidence of Hepatitis B (HB) infection based on the results of testing for HBsAg, anti-HBc and anti-HBs as follows:
    - Subjects positive for HBsAg are excluded
    - Subjects negative for HBsAg and anti-HBc antibody but positive for anti-HBs antibody are eligible to participate
    - Subjects negative for HBsAg and anti-HBs antibody but positive for anti-HBc antibody will require clarification of their status by testing for HB DNA which if positive will exclude the subject from participation.

    Subjects with documented vaccination against Hepatitis B (primary and secondary immunization and booster) will be considered negative.
23. Hepatitis C: Positive test for Hepatitis C antibody confirmed on an additional blood sample by RNA PCR assay. Subjects who are positive for Hepatitis C antibody and negative when the Hepatitis C RNA-PCR assay is performed on an additional sample will be eligible to participate. Subjects who are positive for Hepatitis C antibody and have a positive result for the HCV when the Hepatitis C RNA PCR assay is performed on the additional sample will not be eligible to participate. (Institution or country specific guidelines for blood sample volume limits must be followed in collection of the additional blood sample.)
24. Have a Grade 3 or greater laboratory abnormality based on the protocol toxicity scale <u>except</u> for the following that are allowed:
    - Stable Grade 3 prothrombin time (PT) secondary to warfarin treatment.

- Stable Grade 3 partial thromboplastin time (PTT) due to lupus anticoagulant and not related to liver disease or anti-coagulant therapy.
- Stable Grade 3 hypoalbuminemia due to lupus nephritis, and not related to liver disease or malnutrition.
- Any grade proteinuria
- Stable Grade 3 gamma glutamyl transferase (GGT) elevation due to lupus hepatitis, and not related to alcoholic liver disease, uncontrolled diabetes or viral hepatitis. If present, any abnormalities in the ALT and or AST must be ≤ Grade 2.
- Stable Grade 3 neutropenia; or stable Grade 3 lymphopenia; or stable Grade 3 leukopenia, due to SLE

25. Have a history of an anaphylactic reaction to parenteral administration of contrast agents, human or murine proteins or monoclonal antibodies.

26. Children in Care: A Child in Care (CiC) is a child who has been placed under the control or protection of an agency, organisation, institution or entity by the courts, the government or a government body, acting in accordance with powers conferred on them by law or regulation. The definition of a CiC can include a child cared for by foster parents or living in a care home or institution, provided that the arrangement falls within the definition above. The determination of whether a child meets the definition of CiC should be made with the study centre staff in consultation with the responsible IRB/Ethics Committee.

4.4. Withdrawal Criteria

Subjects may be **withdrawn** from **study agent or from the study** if at any time:

- It is the wish of the subject (or their legally acceptable representative) for any reason.
- The investigator judges it necessary due to medical reasons.

Every effort will be made, in the event that a subject does not choose to continue study agent or to continue to be seen in the clinic according the study schedule, for subjects to be followed, at a minimum, on an annual basis in the long term safety follow-up period.

Furthermore, subjects will be **withdrawn from study agent** and transitioned to Part C, the long term safety follow-up, if at any time:

- Subject becomes pregnant.
- In Part A or Part B, receives prohibited therapy (See Section 5.6.2)
- In Part A are deemed a treatment failure (see Section 5.6.1 and Section 5.6.2)
- Experiences unacceptable toxicity
- Misses 3 or more consecutive study agent infusions

- Participates in another interventional trial.

5. STUDY TREATMENTS

5.1. Study Agent and Other Study Treatment

The trade name of the investigational product is BENLYSTA (belimumab). The generic (USAN/INN) name is belimumab.

Belimumab is a recombinant, human, IgG1λ monoclonal antibody derived by affinity maturation of a parental antibody which itself was derived from screening a phage display library for high affinity binding to BLyS. Belimumab is expressed in the NS0 mouse myeloma cell line. The secreted belimumab is recovered from the growth medium and purified using a series of chromatography and filtration steps.

A summary of the investigational products in this study is presented in Table 1.

Table 1 Summary of Investigational Products in BEL114055

Property	Belimumab
Formulation	Belimumab 400 mg per vial plus excipients (citric acid/sodium citrate/sucrose/polysorbate)
Dosage Form	Reconstituted solution
Unit dose strength	400mg per vial (to contain 80mg/mL when reconstituted with 4.8mL sterile water for injection [SWFI])
Physical description	White uniform lyophilised cake in a 20mL vial
Manufacturer	Human Genome Sciences, Inc.
Route of administration	Intravenous

The contents of the label will be in accordance with all applicable regulatory requirements.

Under normal conditions of handling and administration, study agent is not expected to pose significant safety risks to site staff. Take adequate precautions to avoid direct eye or skin contact and the generation of aerosols or mists. Notify the monitor of any unintentional occupational exposure. A Material Safety Data Sheet (MSDS) describing the occupational hazards and recommended handling precautions will be provided to site staff if required by local laws or will otherwise be available from GSK upon request. Belimumab should be diluted in normal saline after reconstitution, using a typical approved plastic intravenous administration set for the infusion.

Study agent must be stored in a secure area under the appropriate physical conditions for the product, which includes storage in a refrigerator at a temperature of 2-8°C.

Maintenance of a temperature log (manual or automated) is required. Access to and administration of the study agent will be limited to the investigator and authorised site staff. Study agent must be dispensed or administered only to subjects enrolled in the study and in accordance with the protocol.

All used product vials should be destroyed locally according to local site regulations for the destruction of study agents, after the monitor has conducted a check of the product accountability log during the study. At the end of the study, unused product vials will be returned to GSK or destroyed on site according to local regulation at the study closeout visit, after the monitor has conducted final product accountability and given the site approval to destroy or return all remaining supplies.

The 400 mg single use vial of active study agent will be reconstituted with 4.8 mL SWFI, to yield a final concentration of 80 mg/mL of belimumab. After reconstitution and dilution in normal saline, the material is stable for up to 8 hours at 2-8°C, or at room temperature.

The unblinded site pharmacist or unblinded designee will be responsible for receiving, preparing and dispensing study agent, but will be independent of all other study activities.

During the double-blind period all other study site personnel, the subject and the sponsor will remain blinded to the study agent received and to certain biomarkers and pharmacodynamic laboratory results. Separate monitors will be responsible for the clinical (blinded monitor) and study agent (unblinded monitor) aspects of the study

Subjects receiving belimumab will receive the study agent at a concentration of 10 mg/kg or at a modified dose depending on evaluation of data from Cohorts 1 and 2 as described above.

The calculated dose of study agent to be administered to the subject is determined in milligrams (mg) by the assigned treatment group and the subject's body weight in kilograms (kg) at each visit. The study agent should be delivered to all subjects in 250 mL of saline solution. Alternatively, infusion bags with 100 mL saline solution may be considered providing that the resulting belimumab concentration in the infusion bag does not exceed 4 mg/mL. When the study agent is administered at a dose of 10 mg/kg, to maintain a belimumab concentration of 4mg/mL or less, use of the 100 mL bag may only be considered for the subjects whose body weight is <=40 kg. This weight threshold may change as a result of any changes in the dose level of the study agent determined by the evaluation of PK data.

The reconstituted belimumab will be diluted in 250 mL or 100 mL normal saline for IV infusion. An amount of normal saline, equal to the calculated amount of product to be added, should be removed from the 250 mL or 100 mL infusion bag prior to adding the product. After adding the reconstituted product, gently invert the bag to mix the solution. The prepared study agent should be infused over 1 hour.

Placebo will be 250 mL or 100 mL normal saline for IV infusion. To prevent unblinding, the injection membrane on the infusion bag should be punctured with an injection needle.

The dose of study agent administered may not be altered. The rate of infusion may be slowed or interrupted if the subject appears to develop signs of adverse reaction or infusion-associated symptoms. Rate of infusion should not be increased above recommended rate.

The treatment groups consist of an active treatment arm (belimumab) and a placebo arm, with all treatment groups receiving background standard therapy. Once randomized, subjects will be dosed with study agent on Days 0, 14, 28 and then every 28 days through Day 336 (Week 48), resulting in a total of 14 doses. Dosing from Part B of the study will be monthly for up to a total of 120 doses on belimumab.

Subjects will be monitored during all infusions. Subjects will remain under clinical supervision for 3 hours after completion of the first 3 infusions in Part A and for 3 hours after completion of the first 3 infusions in Part B the open label portion according to study sites' guidelines or standard operating procedure for IV infusions. This may include, but is not limited to, monitoring vital signs and observing for any untoward reactions. If symptoms of acute hypersensitivity occur, an extended period of monitoring may be appropriate, based on clinical judgment. Trained personnel and rescue medications/equipment should be available during infusion visits.

Sites are encouraged to follow their standard practices to manage any untoward infusion reactions noted during the infusion period. Subjects and parents/legal guardian should be made aware of the potential risk, the signs and symptoms of such reactions, and the importance of immediately seeking medical attention.

Refer to the Pharmacy Manual for detailed instructions on the preparation, administration and storage of study agent.

If a subject experiences a clinically significant AE that, in the clinical judgment of the investigator, is possibly, probably or definitely related to study agent, and this AE continues at the next scheduled dose, or could potentially be exacerbated by the next dose, the investigator may delay the dose by up to 2 weeks or withhold 1 dose. If a similar concern is present at the time of the next scheduled dose, the investigator should contact the Medical Monitor to determine whether treatment with study agent should be discontinued.

If a subject experiences a clinically significant, potentially life-threatening (Grade 4) AE that in the clinical judgment of the investigator is possibly, probably or definitely related to study agent then treatment with study agent will be discontinued. The subject should be withdrawn from the study, and followed at regularly scheduled monthly study visits as required until resolution of the AE(s), and must also return for follow-up visit 8 weeks after the last dose of study agent.

5.2. Treatment Assignment

Subjects will be assigned to study treatment in accordance with the randomization schedule. Once a randomization number has been assigned to a subject, it cannot be assigned to any other subject in the study.

Subjects will be randomized centrally using the IVRS. This is a telephone based system which will be used by the Investigator or designee to register the subject, randomize the subject and receive medication assignment information. Subjects in Cohort 1 and Cohort 2 will be randomized in a 5:1 ratio and Cohort 3 will be randomized in a 1:1 ratio to 1 of 2 treatment groups: Belimumab (10 mg/kg or adjusted dose) or placebo. Details of how to use the IVRS to register and randomize subjects will be provided in the SPM.

The investigator or designee will obtain the subject's treatment container assignment for use by the unblinded pharmacist or pharmacy designee to prepare and dispense the treatments. Subjects will be identified by the unique subject number assigned at screening that will remain constant for the duration of the study. Each investigator will be supplied with sufficient supplies to conduct the trial. Additional treatments will be supplied as needed to the sites. Although study agent will be stored at the site, the Investigator (or designee) must phone into the IVRS to obtain study agent assignments.

5.3. Blinding

To maintain the integrity of the blind:

- The pharmacist or designee involved in the preparation of the intravenous injections of study agent will be unblinded to the treatment allocation of each subject at that site. The separate site pharmacist or designee will also be the person responsible for receiving and dispensing study agent, but independent of all other study activities.
- Once diluted, in a saline bag as described above belimumab will be identical in appearance to placebo and will be administered by a blinded member of the site staff. All other study site personnel, the subject and the sponsor will remain blinded to the study agent received and to certain biomarkers and pharmacodynamic laboratory results

5.4. Unblinding

The investigator or treating physician may unblind a subject’s treatment assignment **only in the case of an emergency**, when knowledge of the study treatment is essential for the appropriate clinical management or welfare of the subject. Whenever possible, the investigator must first discuss options with the GSK Medical Monitor or appropriate GSK study personnel **before** unblinding the subject’s treatment assignment. If this is impractical, the investigator must notify GSK as soon as possible, but without revealing the treatment assignment of the unblinded subject, unless that information is important for the safety of subjects currently in the study. The date and reason for the unblinding must be recorded in the appropriate data collection tool.

GSK’s Global Clinical Safety and Pharmacovigilance (GCSP) staff may unblind the treatment assignment for any subject with an SAE. If the SAE requires that an expedited regulatory report be sent to one or more regulatory agencies, a copy of the report, identifying the subject’s treatment assignment, may be sent to clinical investigators in accordance with local regulations and/or GSK policy.

5.5. Product Accountability

In accordance with local regulatory requirements, the investigator, designated site staff, or head of the medical institution (where applicable) must document the amount of study agent dispensed and/or administered to study subjects, the amount returned by study subjects, and the amount received from and returned to GSK, when applicable. Product accountability records must be maintained throughout the course of the study.

5.6. Treatment Compliance

Subject compliance with intravenous administration of study medication/placebo will be documented by the administering pharmacist and/or other qualified study personnel in the site records. A subject will be withdrawn if they have missed 3 or more consecutive study agent infusions.

5.6.1. Permitted Medications and Non-Drug Therapies

All concomitant medications taken during the study will be recorded in the eCRF. For all medication types explicitly described in this section, complete information must be recorded on the concomitant medications page.

Subjects must be on a stable SLE treatment regimen for at least 30 days prior to Day 0. New SLE medications other than corticosteroids must not be added within 60 days prior to Day 0. Corticosteroids may be added as new medications or their doses adjusted only up to 30 days prior to Day 0.

Once the subject is randomized and receives the first dose of study agent on Day 0, the investigator may adjust concurrent medications (add, eliminate, change dose level/frequency) as clinically required; however, in Part A changes in certain medications (as outlined below) may result in the subject being defined as a treatment failure and will require withdrawal from Part A of the study and transition to Part C.

5.6.1.1. Anti-malarials

- A new anti-malarial (eg, hydroxychloroquine, chloroquine, quinacrine) may be started between Day 0 and the Day 112 (Week 16) visit.
- The dose of an anti-malarial may be reduced during the course of the study. The dose of an anti-malarial may be increased as clinically required, up to the Day 112 (Week 16) visit.
- After the Day 112 (Week 16) visit, any increase in dose of an anti-malarial over the baseline (Day 0) or Day 112 (Week 16) visit dose, whichever is higher, will declare the subject a treatment failure.
- Starting any new anti-malarial treatment after the Day 112 (Week 16) visit will declare the subject a treatment failure.
- An antimalarial treatment will be considered new if the subject did not receive an antimalarial at any time during the Day 0 to Day 112 (Week 16) treatment interval.

- An anti-malarial may be replaced by another anti-malarial due to documented toxicity or lack of availability at any time during the study.
- The allowable doses of anti-malarial drugs* are:
    - Hydroxychloroquine –up to 400 mg/day.
    - Chloroquine – up to 500 mg/day.
    - Quinacrine – up to 100 mg/day.
    - Compounded anti-malarials – no individual component may exceed the maximum dose above.

*Clinical loading dose is permitted for initiation or replacement.

NOTE: The use of anti-malarials for either SLE management or malarial prophylaxis is permitted.

5.6.1.2. Steroids

In this section, total systemic steroid dose is defined as the average daily dose of all steroids taken IV, IM, SC, intradermally and orally for both SLE and non-SLE reasons. At baseline, the average daily dose of steroids is the sum of steroid dose over 7 consecutive days up to, but not including Day 0, divided by 7. While on treatment, the average daily dose of steroids is the sum of steroid dose over any 7 consecutive days divided by 7 in the respective time window.

5.6.1.2.1. Systemic Steroids for SLE-related Disease Activity

- The total dose of systemic steroids as defined above may be increased as clinically required during the first 6 months of the trial, (i.e., until the Day 168 [Week 24] visit), but must return to within 25% or 5 mg over the baseline (Day 0) dose, whichever is higher, by the Day 168 (Week 24) visit.
- A subject who fails to return to within 25% or 5 mg over the baseline (Day 0) dose, whichever is higher, by the Day 168 (Week 24) visit will be considered a treatment failure. The calculation of the 7-day average steroid dose to determine whether a subject is a treatment failure will begin on the day after the visit.
- After Day 168 (Week 24) visit, an increase > 25% or > 5 mg over the baseline (Day 0) dose, whichever is higher, for SLE activity will deem the subject a treatment failure.
- Within 8 weeks before the Day 364 (Week 52) visit, no new increase over the baseline (Day 0) or Day 308 (Week 44) visit dose, whichever is higher, is allowed.

5.6.1.2.2. Intra-articular Injections

- Subjects may receive intraarticular (IA) injections between baseline (Day 0) and the Day 308 (Week 44) visit.
- A subject who receives any intraarticular injection(s) within 8 weeks before the Day 364 (Week 52) visit will be defined as a treatment failure.

5.6.1.2.3. Steroids for Reasons Other Than SLE Disease Activity

Inhaled and topical steroids are allowed throughout the course of the study. The following time specific restrictions apply to steroid formulations which are not inhaled or topical.

From Day 0 to the Day 168 (Week 24) Visit:

Steroids may be given for reasons other than SLE disease activity (such as asthma, contact dermatitis) as clinically indicated until Day 168 (Week 24) visit.

From Day 168 to 308 (Week 24 to 44) Visits:

Steroids may be given for reasons other than SLE disease activity from the Day 168 (Week 24) visit until the Day 308 (Week 44) visit at any dose/duration that does not result in a total steroid dose (for SLE and non-SLE reasons) > 25% or > 5 mg, whichever is higher, over the baseline dose. In addition, steroids (prednisone or prednisone equivalent) for non-SLE reasons may be given at the investigator’s discretion short-term at higher doses but not to exceed the maximum doses described below.

Up to 750 mg (prednisone or prednisone equivalent) for 1day,

and/or

Up to 100 mg/day (prednisone or prednisone equivalent) for 2-3 days ,

and/or

Up to 40 mg/day (prednisone or prednisone equivalent) for 4-7 days.

The duration of high dose steroid use for reasons other than SLE must not exceed 7 days, after which time, tapering should begin. The total steroid dose must be tapered to within 25% or 5 mg over the baseline dose, whichever is higher, within 30 days of the 1st dose of a course of steroids. In addition, the steroid dose must be tapered to within 25% or 5 mg over the baseline dose, whichever is higher, by the Day 308 (Week 44) visit.

From the Day 308 to Day 364 (Week 44 to 52) Visits:

After the Day 308 (Week 44) visit, no new steroids are allowed for reasons other than SLE activity that result in a total daily steroid dose > 25% or > 5 mg, whichever is higher, over the baseline total steroid dose. A subject will be considered a treatment failure for any steroid use 8 weeks before the Day 364/Exit (Week 52) visit that does not meet this criterion.

5.6.1.2.4. Tapering or Restoration/Restart of Steroids

One of the objectives of this trial is to evaluate the ability of belimumab to reduce juvenile SLE disease activity. Premature or rapid reduction in steroids may confound interpretation of study results and could induce a flare. However, it is understood that an important goal of therapy is to reduce steroids with clinical improvement maintained,

given their associated morbidity. As such, subjects who have clinically significant improvement of SLE disease activity for at least 4 weeks may, at the investigator's discretion, reduce the steroid dose targeting a reduction to 7.5 mg/day or lower. If the subject continues to have stable or improving disease activity after 4 weeks on a reduced dose, then the investigator may consider reducing the dose again. During attempts to wean subjects from steroid use altogether, alternating dosages are permitted to help guard against Addisonian crises. Caution should be exercised when discontinuing steroids before the Day 364/Exit (Week 52) given the potential to induce a flare and thereby confound the study results.

If a subject has had worsening SLE disease activity, then a 12-week period of stable or improving disease activity should be observed before considering a clinically significant steroid dose reduction. (Note: The investigator may always apply their clinical judgment as to whether a dose reduction is appropriate.)

Subjects who have their prednisone or prednisone equivalent tapered may have their dose increased up to the maximum allowed as defined in Section 5.6.1.2.1 and Section 5.6.1.2.3.

5.6.1.2.5. Treatment of SLE Flares with Steroids

If a subject has an SLE flare requiring an increase in steroid dose before the Day 168 (Week 24) visit, the investigator should consider the guidelines prepared for the ACR, for steroid dose/duration of induction therapy [Ad, 2004]. The subject must return to within 25% or 5 mg over the baseline (Day 0) steroid dose, whichever is higher, on or before the Day 168 (Week 24) visit or they will be considered a treatment failure. If a subject's dose is increased > 25% or > 5 mg over the baseline dose, whichever is higher, any time after the Day 168 (Week 24) visit, they are also considered a treatment failure.

5.6.1.3. Other Immunosuppressive/Immunomodulatory Agents

One of the primary objectives of this trial is to evaluate the ability of belimumab to reduce SLE disease activity. Premature or rapid reduction in immunosuppressant/immunomodulatory agents may confound interpretation of study results and could induce a flare.

- Starting any new immunosuppressive/immunomodulatory agent after Day 0 will cause the subject to be declared a treatment failure.
- The dose of existing immunosuppressive/immunomodulatory agents may be increased, as clinically required, up to the Day 112 (Week 16) visit.
- After the Day 112 (Week 16) visit, any increase in dose over the baseline (Day 0) or Day 112 (Week 16) visit dose, whichever is higher, will cause the subject to be declared a treatment failure.

The allowable doses for immunosuppressives at baseline (Day 0) and during the study:

- Azathioprine – up to 300 mg/day.
- 6-mercaptopurine – up to 300 mg/day.

- Mycophenolate mofetil (PO)/ mycophenolate mofetil hydrochloride (IV) – up to 4 g/day.
- Mycophenolate sodium (PO) – up to 2.88 g/day.
- Methotrexate –up to 25 mg/week.
- Oral cyclophosphamide – up to 2.5 mg/kg/day.
- Cyclosporine – up to 4 mg/kg/day*.
- Tacrolimus – up to 0.2 mg/kg/day*.
- Sirolimus – up to 2 mg/day*.
- Thalidomide – up to 200 mg/day.
- Leflunomide – up to 40 mg/day*.
- Mizoribine – up to 150 mg/day.

An immunosuppressive/immunomodulatory agent may be replaced with 1 of the agents above due to documented toxicity or lack of availability. For other immunosuppressive agents not listed above, the investigator must contact the Medical Monitor for approval.

*Clinical loading dose is permitted when replacing immunosuppressive/immunomodulatory agents. Monitor blood levels as clinically indicated.

New topical immunosuppressive agents [e.g., eye drops, topical creams] are allowed after Day 0.

5.6.1.4. Nonsteroidal Anti-inflammatory Drugs (NSAIDs)

Since NSAIDs may affect efficacy parameters (e.g., arthritis) and may affect renal function (NSAID nephropathy), the use of these agents should be initiated and stabilised prior to the screening visit.

- NSAIDs may be given as clinically indicated until the Day 308 (Week 44) visit.
- For subjects who have received an NSAID between Day 0 and Day 308, the existing NSAID can continue at a stable dose after Day 308.
- For subjects who never received an NSAID between the Day 0 and Day 308 (Week 44) visit, starting a new NSAID after the Day 308 (Week 44) visit will declare the subject a treatment failure unless the NSAID is given for < 1 week.
- An NSAID may be replaced with another NSAID due to documented toxicity or lack of availability.
- Anti-thrombotic doses of aspirin are permitted at any time during the study

Paracetamol (acetaminophen) is primarily an analgesic and lacks the anti-inflammatory properties of other NSAIDs. The use of paracetamol is recommended when possible to treat non-SLE related conditions, in the absence of a pre-existing hepatic function deficiency.

5.6.2. Prohibited Medications and Non-Drug Therapies

Subjects who start prohibited medications or therapies at any time during Part A, the double blind placebo controlled portion of the study will be considered treatment failures and nonresponders for primary efficacy analysis. These subjects must have study agent discontinued and will be placed into Part C, the safety follow up phase.

In Part B the belimumab open label safety follow-up, subjects who start prohibited medications or therapies will be considered a protocol violation and will have study treatment (belimumab) discontinued. These subjects will be placed into the Part C safety follow-up period.

The following medications and therapies are prohibited at any time during Part A of the study:

- Other investigational agents (biologic or non-biologic). Investigational applies to any drug not approved for sale in the country in which it is being used.
- Participation in a study using an investigational agent or non-drug therapy that may interfere with the conduct of this protocol.
- Anti-TNF or anti-IL-6 therapy (e.g., adalimumab, etanercept, infliximab, certilizumab, tocilizumab, golimumab).
- Other biologics (e.g., rituximab, abatacept, interleukin-1 receptor antagonist).
- Intravenous immunoglobulin (IVIG).
- IV cyclophosphamide (oral cyclophosphamide is permitted).
- Plasmapheresis, leukapheresis.

For Part B, the belimumab open label safety follow up portion of the study, the above rules also apply with the exception of IV cyclophosphamide, which is permitted in the open label safety follow up. It should be noted that the potential risks of combining belimumab with cyclophosophamide should be factored into the decision to utilize IV cyclophosphamide.

Finally, for Part C, the safety follow-up phase, there are no prohibited medications.

Live Vaccines: Live vaccines are not permitted in the study. Subjects who require a live vaccine during the study should have study agent discontinued prior to receiving the live vaccine.

5.7. Treatment after the End of the Study

Subjects who complete 48 weeks of treatment and the final 52-week evaluation (Part A) will be eligible to participate in Part B, the open label extension period of the study, and receive belimumab.

During Part B, the investigator may adjust concurrent medications (add, eliminate, change dose level/frequency) as clinically appropriate in response to improving or

worsening disease activity. However, subjects who violate the prohibited medication rules (Section 5.6.2) will have belimumab discontinued and subjects placed in Part C.

Investigators are encouraged to consider the guidance provided in Section 5.6.1.2.3 regarding corticosteroid reduction during periods of stable SLE disease. The American College of Rheumatology (ACR) draft guidance regarding steroid dosing and tapering in response to SLE flare is provided in Appendix 7 SLE Flare Index. The reason for altering any immunosuppressant therapy (e.g., toxicity associated with the medication, fluctuations in disease activity, etc) must be recorded on the appropriate eCRF.

The investigator is responsible for ensuring that consideration has been given to care of the subject's medical condition in Part C and post-study.

5.7.1. Study Conclusion

The long term follow up portion of the study (Part B and Part C) is planned for a total of up to 10 years. However, the study will conclude earlier if all subjects continuing belimumab treatment have received at least 5 years of treatment with belimumab and if there are 15 or fewer subjects continuing to receive belimumab in the study.

5.8. Treatment of Study Treatment Overdose

The dose of belimumab considered an overdose has not been defined. There are no known antidotes and GSK does not recommend a specific treatment in the event of a suspected overdose. The investigators will use clinical judgment in treating the symptoms of a suspected overdose.

6. STUDY ASSESSMENTS AND PROCEDURES

Study assessments and procedures will include the following and are detailed in the Time and Events Table 2 and Study Procedures Manual:

- Demographic information including gender, ethnic origin, race, date of birth.
- Medical history.
- Complete physical examination, including height and weight
- Vital signs including temperature, sitting blood pressure, and heart rate
- Confirm classification of SLE disease (based on ACR criteria) by reviewing previously documented clinical records.
- Blood samples for:
  - Haematology.
  - Modified Chem 20 (non-fasting). (CPK MUST be done for subjects with myositis in order to score SELENA SLEDAI/BILAG)
  - Serum pregnancy test - 1 – defined as a pre-menarcheal female who has not yet entered puberty as evidenced by lack of breast development (palpable glandular

breast tissue); or a female who has documentation (medical report verification) of a hysterectomy, has both ovaries surgically removed or tubal ligation.

- HIV antibody, Hepatitis B surface antigen, anti-HBc, anti-HBs and Hepatitis C antibody testing.
- Prothrombin time (PT), partial thromboplastin time (PTT)
- Complement C3, C4
- Serum Immunoglobulin isotypes (IgG, IgM, IgA)
- ANA and anti-dsDNA autoantibodies
- aCL autoantibodies
- PK sampling
- Immunogenicity
- Functional antibodies
- BLyS protein
- B-cell subsets (CD20+, CD20+/27+ memory, CD20+/27– naïve, CD20+/69+ activated, CD20+/138+ plasmacytoid, CD19+/27BRIGHT/38BRIGHT SLE subset and CD20-/138+ plasma cells).

- Urine sample for:
  - Routine urinalysis
  - Spot urine for macroscopic/microscopic/proteinuria assessments
- Disease activity scales:
  - SELENA SLEDAI (See ‘Guidelines for Scoring Proteinuria’)
  - At Screening, confirm SLE disease is active, defined as SELENA SLEDAI score ≥ 6
  - Physician’s Global Assessment (PGA)
  - Parent Global Assessment (ParentGA)
  - SLE Flare Index
  - BILAG
  - Pediatric SLICC/ACR Damage Index
  - PRINTO/ACR Juvenile SLE Response Evaluation
  - Pediatric Quality of Life Inventory (PedsQL)
  - PedsQL-Multidimensional Fatigue Scale

2010N108742_07 **CONFIDENTIAL**

BEL114055

Table 2 Time and Events Table

Procedures	Part A Double-Blind Treatment Period Days 0-364 (Weeks 0-52)															Post Treatment Follow-up		Unscheduled Visit [3]
**Study Day**	**Screening Period -35 days**	Day 0	Day 14 ± 3 days	Day 28 ± 3 days	Day 56 ± 7 days	Day 84 ± 7 days	Day 112 ± 7 days	Day 140 ± 7 days	Day 168 ± 7 days	Day 196 ± 7 days	Day 224 ± 7 days	Day 252 ± 7 days	Day 280 ± 7 days	Day 308 ± 7 days	Day 336 ± 7 days	Day 364 or Exit (4 wks post dose)[1] ± 7 days	8-Week Follow-up ± 7 days [2]	16 Week Follow-Up ± 7 days[19]	
**Study Week**		Wk 0	Wk 2	Wk 4	Wk 8	Wk 12	Wk 16	Wk 20	Wk 24	Wk 28	Wk 32	Wk 36	Wk 40	Wk 44	Wk 48	Wk 52			
Written Informed Consent + Assent	x																		
Subject Demography	x																		
Medical History	x																		
Inclusion/Exclusion Criteria	x																		
**Efficacy Assessments**																			
Disease Activity Scales																			
SELENA SLEDAI[15]	x	x		x	x	x	x	x	x	x	x	x	x	x	x	x	x		
SLE Flare Index	x	x		x	x	x	x	x	x	x	x	x	x	x	x	x	x		
PRINTO/ACR	x	x		x	x	x	x	x	x	x	x	x	x	x	x	x	x		
BILAG[15]	x	x		x	x	x	x	x	x	x	x	x	x	x	x	x	x		
Physician GA	x	x		x	x	x	x	x	x	x	x	x	x	x	x	x	x		
Parent GA	x	x		x	x	x	x	x	x	x	x	x	x	x	x	x	x		

Procedures	Part A Double-Blind Treatment Period Days 0-364 (Weeks 0-52)															Post Treatment Follow-up		Unscheduled Visit [3]
**Study Day**	**Screening Period -35 days**	Day 0	Day 14 ± 3 days	Day 28 ± 3 days	Day 56 ± 7 days	Day 84 ± 7 days	Day 112 ± 7 days	Day 140 ± 7 days	Day 168 ± 7 days	Day 196 ± 7 days	Day 224 ± 7 days	Day 252 ± 7 days	Day 280 ± 7 days	Day 308 ± 7 days	Day 336 ± 7 days	Day 364 or Exit (4 wks post dose)[1]± 7 days	8-Week Follow-up ± 7 days [2]	16 Week Follow-Up ± 7 days[19]	
Pediatric SLICC/ ACR Damage Index		x														x	x		
Quality of Life[16]																			
PedsQL[16]	x	x		x	x	x	x	x	x	x	x	x	x	x	x	x	x		
PedsQL-Fatigue[16]	x	x		x	x	x		x	x			x				x	x		
**Safety Assessments**																			
Concomitant Medication	x	x	x	x	x	x	x	x	x	X	x	x	x	x	x	x	x		x
Symptom Driven Physical Exam	x	x		x	x	x	x	x	x	X	x	x	x	x	x	x	x		x
Vital Signs [4,5]	x	x	x	x	x	x	x	x	x	X	x	x	x	x	x	x	x		x
Weight +Height [5,6]	x	x	x	x	x	x	x	x	x	X	x	x	x	x	x	x	x		
12-Lead ECG	x																		
C-SSRS – (≥12 years of age) Baseline/Screeni	x																		

Procedures	Part A Double-Blind Treatment Period Days 0-364 (Weeks 0-52)															Post Treatment Follow-up		Unscheduled Visit [3]
**Study Day**	**Screening Period -35 days**	Day 0	Day 14 ± 3 days	Day 28 ± 3 days	Day 56 ± 7 days	Day 84 ± 7 days	Day 112 ± 7 days	Day 140 ± 7 days	Day 168 ± 7 days	Day 196 ± 7 days	Day 224 ± 7 days	Day 252 ± 7 days	Day 280 ± 7 days	Day 308 ± 7 days	Day 336 ± 7 days	Day 364 or Exit (4 wks post dose)[1]± 7 days	8-Week Follow-up ± 7 days [2]	16 Week Follow-Up ± 7 days[19]	
ng																			
C-SSRS – Since Last Visit		x	x	x	x	x	x	x	x	X	x	x	x	x	x	x	x		
Adverse Events		x	x	x	x	x	x	x	x	X	x	x	x	x	x	x	x		x
Serious Adverse Events	x	x	x	x	x	x	x	x	x	X	x	x	x	x	x	x	x		x
**Laboratory Assessments**																			
Hematology [7]	x	x		x	x	x	x	x	x	x	x	x	x	x	x	x	x		x
Chemistry (Modified 20 non-fasting) [7]	x	x		x	x	x	x	x	x	x	x	x	x	x	x	x	x		x
Urinalysis	x	x		x	x	x	x	x	x	x	x	x	x	x	x	x	x		x
Spot Urine(protein to creatinine ratio)[8]	x	x	x	x	x	x	x	x	x	x	x	x	x	x	x	x	x		x
PGx Sampling [12]		x																	

**Procedures**	**Part A Double-Blind Treatment Period Days 0-364 (Weeks 0-52)**																**Post Treatment Follow-up**		**Unscheduled Visit** [3]
**Study Day**	**Screening Period -35 days**	Day 0	Day 14 ± 3 days	Day 28 ± 3 days	Day 56 ± 7 days	Day 84 ± 7 days	Day 112 ± 7 days	Day 140 ± 7 days	Day 168 ± 7 days	Day 196 ± 7 days	Day 224 ± 7 days	Day 252 ± 7 days	Day 280 ± 7 days	Day 308 ± 7 days	Day 336 ± 7 days	Day 364 or Exit (4 wks post dose)[1] ± 7 days	8-Week Follow-up ± 7 days [2]	16 Week Follow-Up ± 7 days[19]	
Pregnancy Test [5,9,19]	x	x	x	x	x	x	x	x	x	x	x	x	x	x	x	x	x	x	x
PK Sampling [10,11]		x	x	x	x				x							x	x		x
Immunogenicity		x			x				x							x	x		x
BLys Protein		x																	
C3/C4; CRP	x	x		x	x	x	x	x	x	x	x	x	x	x	x	x	x		
B-Cells		x		x	x	x			x							x	x		
anti-dsDNA	x	x		x	x	x	x	x	x	x	x	x	x	x	x	x	x		
Auto-antibodies [13],	x	x																	
aCL[13]		x																	
IgA, IgG and IgM[14]	x	x	x	x	x	x	x	x	x	x	x	x	x	x	x	x	x		
Functional Antibodies [18]		x							x							x			

2010N108742_07 **CONFIDENTIAL**

BEL114055

**Procedures**	**Part A Double-Blind Treatment Period Days 0-364 (Weeks 0-52)**															**Post Treatment Follow-up**		**Unscheduled Visit [3]**
**Study Day**	**Screening Period -35 days**	Day 0	Day 14 ± 3 days	Day 28 ± 3 days	Day 56 ± 7 days	Day 84 ± 7 days	Day 112 ± 7 days	Day 140 ± 7 days	Day 168 ± 7 days	Day 196 ± 7 days	Day 224 ± 7 days	Day 252 ± 7 days	Day 280 ± 7 days	Day 308 ± 7 days	Day 336 ± 7 days	Day 364 or Exit (4 wks post dose)[1]± 7 days	8-Week Follow-up ± 7 days [2]	16 Week Follow-Up ± 7 days[19]	
Vaccine Antibody Titer [18]																			
PT/PTT	x																		
**Study Treatment**																			
Administer Study Treatment [17]		x	x	x	x	x	x	x	x	x	x	x	x	x	x	only if going into Part B			
Randomization		x																	
IVRS	x	x	x	x	x	x	x	x	x	x	x	x	x	x	x	x	x		x

Time and Events Table – Open-Label and Follow-up Periods

Procedures	Part B Open-Label Continuation				Part C 10 Year Post Infusion Safety Follow-up	
	Assessment Every 4 weeks ± 7days	Additional Assessments Every 6 Months/Yearly ±7 days	Exit Visit[2] and 8-Week Post Treatment Follow-up ±7 days [2]	16 Week Post Treatment Follow Up phone call ±7 days [19]	Assessment Every 4 weeks ± 7 days for the First 3 Months^	Assessments Every Year ± 28 days after last infusion[#]
**Efficacy Assessments**						
Disease Activity Scales: SELENA, SLEDAI,SLE Flare Index, BILAG, Physician GA, Parent GA[15]		x	x			
Pediatric SLICC/ACR Damage Index[16]		x	x			x
PedsQL [16]		x	x			x
PedsQL-Multidimensional Fatigue[16]		x	x			x
**Safety Assessments**						
Concomitant Medication	X		x		x	x
Symptom Driven Physical Examination	X		x		x	x
Vital Signs	X		x		x	x
Weight and Height[5,6]	X		x		x	x

Brunner HI, *et al. Ann Rheum Dis* 2020; 79:1340–1348. doi: 10.1136/annrheumdis-2020-217101

Procedures	Part B Open-Label Continuation				Part C 10 Year Post Infusion Safety Follow-up	
	Assessment Every 4 weeks ± 7days	Additional Assessments Every 6 Months/Yearly ±7 days	Exit Visit[2] and 8-Week Post Treatment Follow-up ±7 days [2]	16 Week Post Treatment Follow Up phone call ±7 days [19]	Assessment Every 4 weeks ± 7 days for the First 3 Months^	Assessments Every Year ± 28 days after last infusion[#]
Adverse Events (AEs)	X	X*	x		x	
Serious AEs (SAEs) and AEs of Special Interest (AESIs)	X	X*	x		x	x*
**Laboratory Assessments**						
Hematology	x		x		x	x
Chemistry (Modified 20 non-fasting)	x		x		x	x
B-cells		X	X			X
Immunogenicity		X	X		X**	
Anti-dsDNA; C3/C4		X				
IgA, IgG and IgM[14]	X	X	X		X	X
Spot Urine (protein to creatinine ratio)[8]		X	X			
Pregnancy Test [5,9,19]	X		X	X	X	

Procedures	Part B Open-Label Continuation				Part C 10 Year Post Infusion Safety Follow-up	
	Assessment Every 4 weeks ± 7days	Additional Assessments Every 6 Months/Yearly ±7 days	Exit Visit[2] and 8-Week Post Treatment Follow-up ±7 days [2]	16 Week Post Treatment Follow Up phone call ±7 days [19]	Assessment Every 4 weeks ± 7 days for the First 3 Months^	Assessments Every Year ± 28 days after last infusion#
PK Sampling [20]	X (Japan only, at only week12 / 3 month visit of first year)	X (Japan only, at only 6 month visit of first year)				
**Study Treatment**						
Administer Study Treatment [17]	X					
IVRS	X	X	X	X	X	X

* Adverse events of special interest (including serious and non-serious events) will be collected every 6 months in Part B and annually in Part C (See Section 8.3.5.3.2 for definition of Adverse Events of Special Interest)
^ For subjects that withdraw from the study 8 weeks or less from the last administration of IV belimumab, an 8 Week Follow-up Visit (and a 16 Week Follow-up Visit post administration for female subjects of child-bearing potential) must be performed and recorded in the IVR system and the eCRF.
# Exit visit for any subject who withdraws completely from Part C after the first 3 month period may be conducted as a phone contact to close out the study.
** Serum sample for anti-belimumab antibodies only required at first monthly visit in Part C (approximately 8 weeks after last dose)

Brunner HI, *et al. Ann Rheum Dis* 2020; 79:1340–1348. doi: 10.1136/annrheumdis-2020-217101

Time and Events Table – (Continued)

1. The Exit (Day 364) visit will occur approximately 4 weeks after the last dose of study agent. For subjects completing all 48 weeks of treatment and continuing into the safety follow-up with belimumab portion of the protocol (Part B) or safety follow up (Part C), this visit will also serve as their 1st (i.e., Day 0) visit of the open label safety follow up of the study. Only subjects who will continue in Part B will have study drug administered at the Day 364/Week 52 visit after the completion of all Day 364/Week 52 assessments.
2. Subjects stopping treatment in Part A or Part B will return in 4 weeks from last dose for an Exit visit. For subjects withdrawn from Part A or Part B of the study and who are not continuing into Part C, a follow-up visit will occur approximately 8 weeks after last dose of study agent (belimumab in the case of the open label period).
3. Other assessments may be performed as clinically indicated.
4. Vital signs include temperature, sitting blood pressure, and pulse.
5. Complete prior to dosing.
6. The subject's weight at the current visit should be used for calculating the dose to be administered.
7. Refer to Appendix 5 Clinical Laboratory Tests for a listing of laboratory assessments to be completed.
8. A 24-hour urine may be done if clinically indicated (e.g., renal flare).
9. Serum pregnancy test required at screening for all females of childbearing potential. Results of urine pregnancy test at subsequent visits, if required, must be available prior to dose. See Section 6.1 (Screening Procedures) for definition of those exempted from subsequent pregnancy testing.
10. Cohort 1 and Cohort 2: Days 0, 14/Wk2, 28/Wk4, 56/Wk8 - Before the start of infusion, 5 minutes and 2 hours after the end of infusion; Days 2, 7, 16, 21 - Anytime during visit; Day 168/wk24 - 0-4 hours after the end of infusion; Day 364/Wk52 and 8 week follow-up visit - Any time during visit before start of infusion (if applicable). Cohort 3 PK sample schedule: Day 0, 14, 28 – Before the start of infusion and 0-4 hrs after the end of infusion; Day 56 – Before the start of infusion; Day 168/wk24 – 0-4 hrs after the end of infusion; Day 364/wk52 and 8 week follow-up visit – any time during visit before start of infusion (if applicable). [see Table 3 and Table 4 in Section 6.5]
11. Cohort 1 (12-17yrs) subjects (N=12) and Cohort 2 (5-11 yrs) (at least 10 subjects) will be subject to more frequent sampling for PK and intense observation during the study are also detailed in Section 6.5**,** Table 3 of the protocol.
12. PGx informed consent must be obtained prior to any saliva being taken for PGx research. Refer to Section 9 and Appendix 1 Pharmacogenetic Research. Samples should be drawn prior to dosing but may be taken at anytime during the course of the study.
13. Autoantibodies include: ANA, aCL (IgM, IgG, IgA isotypes), anti-ribosomal P and anti-Sm. Autoantibodies will be measured in all subjects at Day 0 and samples will be collected at the time points specified; however, the assay will be run only on subjects with elevated titers of these autoantibodies at Day 0.
14. Serum immunoglobulin isotypes: IgG, IgM, IgA.
15. Refer to Section 6.2.1.3 for guidelines for scoring proteinuria for SELENA SLEDAI evaluation.
16. Subjects ≥ 8 years of age will complete the PedsQL and PedsQL Fatigue directly. For subjects aged 5-7 years, a parent/guardian will complete the Parent Report version of the PedsQL and PedsQL Fatigue on their child's behalf. Must be completed prior to any study-related discussion with the investigator or study coordinator.
17. Study agent is to be administered to all randomized subjects for 48 weeks in Part A. Subjects completing the double-blind period (Part A) will continue in the open-label, long term continuation portion of the protocol for up to 10 years. The Day 364/Week 52 visit of Part A will serve as the 1st visit of Part B. Only subjects who will continue in Part B will have study drug administered at the Day 364/Week 52 visit after the completion of all Day 364/Week 52 assessments.
18. Blood sample for functional antibodies on all subjects. Vaccine antibody titer test will be completed on subjects who receive a vaccine during the treatment period of this study. A blood sample to measure the pre-vaccination titer will be obtained during a study visit closest to the time prior to the planned vaccination In cases where blood may not be drawn immediately before vaccination, the baseline (Day 0) blood sample will be used as a reference for pre-vaccination titer. A post-vaccine titer will be drawn on the next study visit if it has been at least 21 to 60 days post-immunization.
19. A home pregnancy test will be provided to all female subjects of child bearing potential. These subjects will be contacted by phone approximately 16 weeks after the last dose of belimumab to obtain the results of the test. AEs/SAEs will not be actively solicited, but SAE(s) reported by the subject during this call will be reported to the Drug Safety designee on the SAE worksheet.
20. For subjects enrolled in Japan only, additional PK samples (before the start of infusion and 0-4 hours after the end of infusion) will be taken at the week 12 (3 month) and at the first 6 month visit only in Part B

6.1. Critical Screening and Baseline Assessments

All screening assessments must be performed within 35 days prior to the baseline visit. The ANA/anti-ds DNA autoantibody screening assessments may be repeated once during the 35-day window from screening to the baseline visit if a positive historical test is not being used to supplement the screening visit result to meet Inclusion criteria 4 (Section 4.2)

Screening assessments at Visit 1 (Week -5/Day -35) or Randomization assessments at Visit 2 (Week 0/Day 0) as detailed in Table 2 will comprise the following:

- Demographic information including gender, ethnic origin, race, date of birth.
- Medical history.
- Complete physical examination, including height, weight (further details are provided in Section 6.3.19 and Section 6.3.18).
- Vital signs including temperature, sitting blood pressure, and heart rate (further details are provided in Section 6.3.18).
- 12-lead ECG at screening
- Confirm classification of SLE disease (based on ACR criteria) by reviewing previously documented clinical records.
- Blood samples for:
  - Haematology
  - Modified Chem 20 (non-fasting). (CPK MUST be done for subjects with myositis in order to score SELENA SLEDAI/BILAG)
  - Serum pregnancy test - for all women with an intact uterus, unless exempted from pregnancy testing (ie, of non-childbearing potential – females who are pre-menarcheal, women who had a hysterectomy, are post-menopausal which is defined as 1 year without menses, have both ovaries surgically removed or have current documented tubal ligation).
  - HIV antibody, Hepatitis B surface antigen, anti-HBc, anti-HBs and Hepatitis C
  - Prothrombin time (PT), partial thromboplastin time (PTT)
  - Biological markers (Complement C3, C4)
  - Serum Immunoglobulin isotypes (IgG, IgM, IgA)
  - ANA and anti-dsDNA autoantibodies
  - aCL autoantibodies
  - PK sampling
  - Immunogenicity
  - Functional antibodies
  - Vaccine Antibody titer assessment

- BLyS protein
- B-cell subsets ($CD20^{+}$, $CD20^{+}/27^{+}$ memory, $CD20^{+}/27^{-}$ naïve, $CD20^{+}/69^{+}$ activated, $CD20^{+}/138^{+}$ plasmacytoid, $CD19^{+}/27^{BRIGHT}/38^{BRIGHT}$ SLE subset and $CD20^{-}/138^{+}$ plasma cells).

- Urine sample for:
  - Routine urinalysis.
  - Drug screen.
  - Spot urine for macroscopic/microscopic/proteinuria assessments.
- Disease activity scales:
  - SELENA SLEDAI (See 'Guidelines for Scoring Proteinuria' Section 6.2.1.3.1).
    - At screening, confirm SLE disease is active, defined as SELENA SLEDAI score ≥ 6.
  - Physician's Global Assessment (PGA).
  - SLE Flare Index.
  - BILAG.
  - Pediatric SLICC/ACR Damage Index.
  - PRINTO/ACR Juvenile SLE Response Evaluation
  - Pediatric Quality of Life Inventory (PedsQL)
  - PedsQL-Multidimensional Fatigue Scale

6.2. Efficacy

6.2.1. SELENA SLEDAI

All site staff scoring SELENA SLEDAI are required to pass proficiency tests before carrying out assessments to ensure consistency across centres.

6.2.1.1. SELENA SLEDAI Score

The SLEDAI (Systemic Lupus Erythematosus Disease Activity Index) is a validated index for assessing SLE disease activity [Bombardier, 1992]. It is a weighted index in which signs and symptoms, laboratory tests, and physician's assessment for each of 9 organ systems are given a weighted score and summed, if present at the time of the visit or in the preceding 10 days:

- Score of 8 each for CNS and vascular items.
- Score of 4 each for renal and musculoskeletal items.
- Score of 2 each for serosal, dermal, and immunologic items.
- Score of 1 each for constitutional and hematologic items.

The SELENA SLEDAI used in this study is a slightly modified version of the SLEDAI developed for a National Institutes of Health-sponsored multicentre study of estrogen/progesterone hormone use in women with SLE [Buyon, 2005; Petri, 2005]. The descriptions for some of the items are are slightly modified, but the organ systems and weighted scores are the same as the SLEDAI. The maximum theoretical score for the SELENA SLEDAI is 105 (all 24 descriptors present simultaneously) with 0 indicating inactive disease. Completion of the index requires collection of a 24-hour urine sample for assessment of proteinuria (although spot urine protein:creatinine ratio is commonly substituted in practice, see Section 6.2.1.2), measurement of anti-dsDNA, C3, C4, haematology, and urinalysis, and for subjects with myositis, CPK. Guidelines for scoring proteinuria are provided in Section 6.2.1.3.

SELENA SLEDAI will be assessed in Part A at screening and prior to dosing at Day 0, Weeks 4, 8, 12, 16, 20, 24, 28, 32, 36, 40, 44, 48, and 52/or Exit, 8 week follow-up (Part A) and every 6 months in Part B and 8 week follow-up (Part B).

A copy of the index is provided in Appendix 6 SELENA SLEDAI Disease Assessment Scales.

6.2.1.2. Laboratory Tests for SELENA SLEDAI and BILAG

It has been demonstrated that there is a strong correlation between the protein content of a 24-hour urine collection and the protein:creatinine ratio in a single urine sample [Ginsberg, 1983; Ruggenenti, 1998; KDOQI Clinical Practice Guidelines for Chronic Kidney Disease, 2002; Price, 2005]. Given this information, spot urine protein:creatinine ratio will be used for determining proteinuria in this study for both the SELENA SLEDAI and BILAG disease activity indices.

Measurement of creatinine clearance (CrCl)/glomerular filtration rate (GFR) using timed (for example, 24-hour) urine collections is time consuming and error prone and has consistently been shown to be no more, and often less, reliable than serum creatinine based equations for the estimation of GFR [KDOQI Clinical Practice Guidelines for Chronic Kidney Disease, 2002]. Therefore, GFR estimated by the Schwartz formula or Cockroft-Gault formula will be used in the BILAG disease activity index. The procedure for calculation of GFR using the Schwartz formula or Cockroft-Gault formula will be provided in the Study Procedure Manual. The age appropriate formula should be used.

6.2.1.3. Guidelines for Scoring Proteinuria for SELENA SLEDAI

The following guidelines should be followed for scoring for proteinuria in the SELENA SLEDAI disease activity index.

6.2.1.3.1. Scoring for Proteinuria for Eligibility and at Screening

In order to be assigned 4 points for proteinuria at screening, the screening spot urine proteinuria assessment must show:

- Proteinuria > 1 g/24 hour equivalent (irrespective of previous value).

OR

- New onset or recurrent proteinuria > 0.5 g/24 hour equivalent or a > 0.5 g/24 hour equivalent increase above the previously documented 24-hour proteinuria or equivalent value obtained within 26 weeks of the screening value. If the screening value is ≤ 1 g/24 hour equivalent and there are no previous assessments of 24-hour proteinuria or spot urine protein to creatinine ratio available within 26 weeks for a given subject, 4 points cannot be given for proteinuria on the SELENA SLEDAI index, unless a 2nd assessment is done during the screening period of the study (ie, before Day 0).

6.2.1.3.2. Scoring for Proteinuria at Day 0 and Subsequent Study Visits

According to the SELENA SLEDAI scoring rules, unless the proteinuria continues to rise such that it has increased by > 0.5 g/24 hour equivalent at Day 0 (ie, baseline), the subject, by default, will have an improving SELENA SLEDAI score prior to treatment. Two assessments of 24-hour proteinuria (by spot urine protein to creatinine ratio) obtained in a 2 to 3 week period are unlikely to show a > 0.5 g/24 hour equivalent increase except in acute renal flare. This is problematic for data analysis since the percent change in the disease activity scales are calculated from the baseline (not screening) SELENA SLEDAI score. As such, the following scoring rules will be applied:

Scoring for a Subject with Proteinuria and 4 Points Assigned in SELENA SLEDAI

If there is an increase from the last visit of > 0.5 g/24 hour equivalent, only 4 points for proteinuria will continue to be applied (so no subject can get more than 4 points for proteinuria at any 1 time point).

If the proteinuria has not improved (ie, there has not been a decrease in proteinuria of > 0.5 g/24 hour equivalent) since the previous assessment, then 4 points will continue to be assigned on the SELENA SLEDAI index at the current visit.

If proteinuria has improved (decrease of > 0.5 g/24 hour equivalent or a decrease to ≤ 0.5 g/24 hour equivalent) from the previous visit to the current visit, then 0 points will be assigned on the SELENA SLEDAI index at the current visit.

Scoring for a Subject with 0 Points for Proteinuria in SELENA SLEDAI

If the proteinuria score for SELENA SLEDAI is 0 and at the subsequent visit the assessment of 24-hour proteinuria (by spot urine protein to creatinine ratio) shows > 0.5 g/24 hour equivalent increase above the previous value or the subject develops new onset of proteinuria > 0.5 g/24 hour equivalent, 4 points will be assigned at this current visit.

6.2.1.4. SELENA SLEDAI Flare Index

The SLE Flare Index (SFI) categorises SLE flare as "mild or moderate" or "severe" based on a positive assessment for at least one of 5 variables [Buyon, 2005; Petri, 2001; Petri, 2005].

- Change in SELENA SLEDAI score from the most recent assessment to current. (not used for severe flare)
- Change in signs or symptoms of disease activity.
- Change in prednisone or prednisone equivalent dosage.
- Use of new medications for disease activity or hospitalization.
- Change in PGA score.

Hospitalization for SLE activity is an additional category included only for a severe flare. In this study a modification of the SFI will be used in which a severe flare will not be counted if it is triggered only by an increase in SELENA SLEDAI score.

A copy of the SLE Flare Index is provided in Appendix 7 SLE Flare Index

6.2.1.5. Physician's Global Assessment (PGA)

The PGA is a 10 cm visual analogue scale (VAS), anchored at 0 (none) and 3 (severe), designed for the physician to indicate the subject's overall disease activity at a particular visit as part of the validated SELENA SLEDAI index. Either the primary investigator or a subinvestigator will score the PGA for the subject, and the same person will evaluate the subject each time, unless agreed with the GSK medical monitor. Each PGA measurement will be transformed linearly (x 3/10) by the GSK statistician to obtain a value between 0.00 and 3.00.

PGA will be assessed at screening and prior to dosing at Day 0, Weeks 4, 8, 12, 16, 20, 24, 28, 32, 36, 40, 44, 48, and 52/or Exit, 8 week follow-up (Part A) and every 6 months in Part B and 8 week follow-up (Part B).

A copy of the PGA VAS is provided in Appendix 8 Physician's Global Disease Assessment.

6.2.1.6. Parent 's Global Assessment (ParentGA)

The Parent's Global Assessment (ParentGA) assesses the subject's overall well-being **at the moment** rated on a 21-numbered circle visual analog scale (VAS; 0 - very well, 10 - very poorly). The ParentGA will be assessed at screening and prior to dosing at Day 0, Weeks 4, 8, 12, 16, 20, 24, 28, 32, 36, 40, 44, 48, and 52/or Exit, 8 week follow-up (Part A) and every 6 months in Part B and 8 week follow-up (Part B).

A copy of the ParentGAVAS is provided in Appendix 9 Parent's Global Disease Assessment: Parent's Global Disease Assessment.

6.2.2. Pediatric Rheumatology International Trials Organization/ACR (PRINTO/ACR Juvenile SLE Response Evaluation)

The Pediatric Rheumatology International Trials Organization (PRINTO)/American College of Rheumatology (ACR) criteria for evaluation of juvenile SLE include objective measures for assessing clinical improvement. The five PRINTO/ACR Juvenile SLE Response Evaluation lupus lupus disease activity measures include assessments in the following domains: Physician's Global Assessment of overall disease activity utilizing a 10-cm visual analog scale (VAS); Parent's Global Assessment of the subject's overall well-being (21 circle VAS); 24 hour proteinuria to measure renal involvement; SELENA-SLEDAI to measure disease activity; and the PedsQL Generic Core physical functioning domain The individual measurements will be assessed at screening and prior to dosing at Day 0, Weeks 4, 8, 12, 16, 20, 24, 28, 32, 36, 40, 44, 48, and 52/or Exit, 8 week follow-up and every 6 month in the open label safety follow up (Part B), 8 week follow-up visit (Part B).

6.2.3. BILAG

The BILAG index is a clinical measure of lupus disease activity. The scoring system for the BILAG index was developed based upon the principle of the physician's intention to treat. The main distinguishing feature of the BILAG index from other disease activity indices is that disease activity in different organs/systems is reported separately. There are 8 organ systems: general, mucocutaneous, neurological, musculoskeletal, cardiorespiratory, vasculitis, renal, and hematological [Hay, 1993; Isenberg, 2000; Gordon, 2003]. A score is calculated for each system depending on the SLE clinical manifestations (or signs and symptoms) present and whether they are new, worse, the same, improving, or not present in the last 4 weeks compared with the previous 4 weeks. The SLE disease manifestations considered severe in each system are those that would normally require high dose steroids (prednisolone > 20 mg/day or equivalent) and/or cytotoxic agents; these define a BILAG A score. More moderate SLE disease manifestations that would be considered appropriate to treat with lower dose steroids, antimalarial drugs or NSAIDs contribute to a BILAG B score. Mild symptomatic SLE features that require only symptomatic therapy (e.g., analgesics and NSAIDs) contribute to a C score. If there are no current symptoms, but the system has previously been involved, then a D is recorded, while if the system has never been involved an E score is assigned.

The BILAG will be assessed at screening and prior to dosing at Day 0, Weeks 4, 8, 12, 16, 20, 24, 28, 32, 36, 40, 44, 48, and 52//Exit visit, 8 week follow-up (Part A), and every 6 months and 8 week follow-up in Part B.

A copy of the BILAG index is provided in Appendix 10 BILAG Index Assessment. All site staff scoring BILAG assessments are required to pass proficiency tests before carrying out assessments to ensure consistency across centres.

Brunner HI, *et al*. *Ann Rheum Dis* 2020; 79:1340–1348. doi: 10.1136/annrheumdis-2020-217101

6.2.4. Pediatric SLICC/ACR Damage Index

The pediatric Systemic Lupus International Collaborative Clinics/American College of Rheumatology (SLICC/ACR) Damage Index is a modification of the validated instrument developed to assess the accumulated damage in subjects with SLE, resulting from either the disease process or its sequelae.

The SLICC/ACR Damage Index can identify changes in damage seen in subjects with both active and inactive disease and records damage occurring in subjects with SLE regardless of its cause [Ginsberg, 1983; Gladman, 1996]. The index is a predictor of severe outcome and an indicator of morbidity in different ethnic groups [Stoll, 1996]. SLICC/ACR Damage Index was designed to be useful both as a descriptor for subject populations included in studies, and as an outcome measure for therapeutic trials and studies of prognosis [Ginsberg, 1983; Gladman, 1996].

The pediatric modified SLICC/ACR Damage Index consists of 41 items in 12 different organ systems. In addition to the items included in the SLICC/ACR Damage Index, the modified index includes assessments to evaluate growth failure and delayed puberty which are believed important in the assessment of damage in juvenile SLE [Gutiérrez, 2006].

In order for a feature to represent damage, it had to be present for at least 6 months, to enable discrimination between active inflammation and tissue damage [Ginsberg, 1983; Gladman, 1996].

Scoring is usually 0 or 1, although in 6 items, a score of 2 can be given if there is a repeat episode more than 6 months apart. The exception is end-stage renal disease, which scores 3.

Pediatric SLICC/ACR Damage Index will be assessed at Day 0 and the/52/or Exit visit and 8 week follow-up visit in Part A; every 6 months in Part B and 8 week follow-up visit (Part B); and annually in Part C.

A copy of the pediatric SLICC/ACR Damage Index is provided in Appendix 11 Pediatric SLICC/ACR Damage Index [Hay, 1993; Isenberg, 2000].

6.3. Safety

For Part A, the safety assessments will be as follows:

- Adverse Event (including infusion-related and hypersensitivity reactions, infections and malignancies) reported throughout the 52-week treatment period and 8 week follow-up.
- Haematological and clinical chemistry parameters (including urinalysis) throughout the 52-week treatment period and 8 week follow-up.
- Vital signs (i.e., pulse rate and systolic and diastolic blood pressure) throughout the 52-week treatment period and 8 week follow-up.

- Physical examination
- Suicidality assessment (for subjects ≥ 12 years of age)
- Immunogenicity during the 52-week treatment period and 8 week follow-up. A 6 month follow-up assessment will be included for all subjects that have a positive anti-belimumab antibody response at the 8 week follow-up assessment.
- Additional safety tests (such as vital signs, physical examinations and laboratory safety tests) or change in timing or addition of assessments may be performed during the course of the study based on newly available data to ensure appropriate safety monitoring. IRB/IEC will be informed of any safety issues that require alteration of the safety monitoring scheme.

6.3.1. Liver Chemistry Stopping and Monitoring Criteria

Liver chemistry stopping and increased monitoring criteria have been designed to assure subject safety and evaluate liver event etiology in alignment with the FDA premarketing clinical liver safety guidance [James, 2009; Le Gal, 2005].

Liver Chemistry Stopping Criteria- Liver Stopping Event	
ALT-absolute	ALT ≥ 8xULN
ALT Increase	ALT ≥ 5xULN but <8xULN persists for ≥2 weeks<br>ALT ≥ 3xULN but <5xULN persists for ≥4 weeks
Bilirubin[1, 2]	ALT ≥ 3xULN and bilirubin ≥ 2xULN (>35% direct bilirubin)
INR[2]	ALT ≥ 3xULN and INR>1.5, if INR measured
Cannot Monitor	ALT ≥ 5xULN but <8xULN and cannot be monitored weekly for ≥2 weeks<br>ALT ≥ 3xULN but <5xULN and cannot be monitored weekly for ≥4 weeks
Symptomatic[3]	ALT ≥ 3xULN associated with symptoms (new or worsening) believed to be related to liver injury or hypersensitivity

1) Serum bilirubin fractionation should be performed if testing is available. If serum bilirubin fractionation is not immediately available, discontinue study treatment for that subject if ALT ≥ 3xULN **and** bilirubin ≥ 2xULN. Additionally, if serum bilirubin fractionation testing is unavailable, **record presence of detectable urinary bilirubin on dipstick**, indicating direct bilirubin elevations and suggesting liver injury.
2) All events of ALT ≥ 3xULN **and** bilirubin ≥ 2xULN (>35% direct bilirubin) or ALT ≥ 3xULN **and** INR>1.5, if INR measured which may indicate severe liver injury (possible 'Hy's Law'), **must be reported as an SAE (excluding studies of hepatic impairment or cirrhosis)**; INR measurement is not required and the threshold value stated will not apply to subjects receiving anticoagulants.
3) New or worsening symptoms believed to be related to liver injury (such as fatigue, nausea, vomiting, right upper quadrant pain or tenderness, or jaundice) or believed to be related to hypersensitivity (such as fever, rash or eosinophilia).

6.3.1.1. Required Actions and Follow up Assessments following ANY Liver Stopping Event

ACTIONS:

- Immediately discontinue study treatment
- Report the event to GSK **within 24 hours**
- Complete the liver event CRF and complete SAE data collection tool if the event also meets the criteria for an SAE (All events of ALT ≥ 3xULN and bilirubin ≥ 2xULN (>35% direct bilirubin) or ALT ≥ 3xULN and INR>1.5, if INR measured which may indicate severe liver injury (possible 'Hy's Law'), must be reported as an SAE (excluding studies of hepatic impairment or cirrhosis); INR measurement is not required and the threshold value stated will not apply to subjects receiving anticoagulants)
- Perform liver event follow up assessments
- Monitor the subject until liver chemistries resolve , stabilize, or return to within baseline (see **MONITORING** below)

Do not restart/rechallenge subject with study treatment unless allowed per protocol and GSK Medical Governance approval **is granted** (refer to [Appendix 4]).

MONITORING

<u>For bilirubin or INR criteria:</u>

- Repeat liver chemistries (include ALT, AST, alkaline phosphatase, bilirubin) and perform liver event follow up assessments within **24 hours**
- Monitor subjects twice weekly until liver chemistries resolve, stabilize or return to within baseline
- A specialist or hepatology consultation is recommended

<u>For All other criteria:</u>

- Repeat liver chemistries (include ALT, AST, alkaline phosphatase, bilirubin) and perform liver event follow up assessments within **24-72 hours**
- Monitor subjects weekly until liver chemistries resolve, stabilize or return to within baseline

FOLLOW UP ASSESSMENTS

- Viral hepatitis serology (includes: Hepatitis A IgM antibody; Hepatitis B surface antigen and Hepatitis B Core Antibody (IgM); Hepatitis C RNA; Cytomegalovirus IgM antibody; Epstein-Barr viral capsid antigen IgM antibody (or if unavailable, obtain heterophile antibody or monospot testing); Hepatitis E IgM antibody)
- Blood sample for pharmacokinetic (PK) analysis, obtained within approximately one to two weeks after last dose (PK sample may not be required for subjects

known to be receiving placebo or non-GSK comparator treatments.) Record the date/time of the PK blood sample draw and the date/time of the last dose of study treatment prior to blood sample draw on the CRF. If the date or time of the last dose is unclear, provide the subject's best approximation. If the date/time of the last dose cannot be approximated OR a PK sample cannot be collected in the time period indicated above, do not obtain a PK sample. Instructions for sample handling and shipping are in the SPM.

- Serum creatine phosphokinase (CPK) and lactate dehydrogenase (LDH).
- Fractionate bilirubin, if total bilirubin≥2xULN
- Obtain complete blood count with differential to assess eosinophilia
- Record the appearance or worsening of clinical symptoms of liver injury, or hypersensitivity, on the AE report form
- Record use of concomitant medications on the concomitant medications report form including acetaminophen, herbal remedies, other over the counter medications
- Record alcohol use on the liver event alcohol intake case report form

For bilirubin or INR criteria:

- Anti-nuclear antibody, anti-smooth muscle antibody, Type 1 anti-liver kidney microsomal antibodies, and quantitative total immunoglobulin G (IgG or gamma globulins).
- Serum acetaminophen adduct HPLC assay (quantifies potential acetaminophen contribution to liver injury in subjects with definite or likely acetaminophen use in the preceding week [James, 2009]). **NOTE: not required in China**
- Liver imaging (ultrasound, magnetic resonance, or computerised tomography) and /or liver biopsy to evaluate liver disease; complete Liver Imaging and/or Liver Biopsy CRF forms.

6.3.2. Increased Monitoring Criteria with Continued Therapy

If met see required actions below:

- If ALT ≥5xULN and <8xULN **and** bilirubin <2xULN **without** symptoms believed to be related to liver injury or hypersensitivity, **and** who can be monitored weekly for 2 weeks OR
- ALT ≥3xULN **and** <5xULN and bilirubin <2xULN **without** symptoms believed to be related to liver injury or hypersensitivity, **and** who can be monitored weekly for 4 weeks

6.3.2.1. Required Actions and Follow Up Assessments for Increased Monitoring with Continued Therapy

- Notify the GSK medical monitor **within 24 hours** of learning of the abnormality to discuss subject safety.
- Subject can continue study treatment
- Subject must return weekly for repeat liver chemistries (ALT, AST, alkaline phosphatase, bilirubin) until they resolve, stabilise or return to within baseline
- If at any time subject meets the liver chemistry stopping criteria, proceed as described above for Required Actions and Follow up Assessments following ANY Liver Stopping Event
- If ALT decreases from ALT ≥5xULN and <8xULN to ≥3xULN but <5xULN, continue to monitor liver chemistries weekly.
- If, after 4 weeks of monitoring, ALT <3xULN and bilirubin <2xULN, monitor subjects twice monthly until liver chemistries normalize or return to within baseline.

6.3.3. Study Treatment Restart

Restart refers to resuming study treatment following liver stopping events in which there is a clear underlying cause (other than drug induced liver injury [DILI]) of the liver event (e.g. biliary obstruction, pancreatic events, hypotension, acute viral hepatitis). Furthermore, there should be no evidence of alcoholic hepatitis or hypersensitivity, and the study treatment should not be associated with HLA markers of liver injury.

Note: At investigator sites in the Russian Federation, restart of study treatment after liver event is not permitted.

Approval by GSK for study treatment restart can be considered where:

- Investigator requests consideration for study treatment restart if liver chemistries have a clear underlying cause (e.g., biliary obstruction, hypotension and liver chemistries have improved to normal or are within 1.5 x baseline and ALT <3xULN).
- Restart risk factors (e.g., fever, rash, eosinophilia, or hypersensitivity, alcoholic hepatitis, possible study treatment-induced liver injury or study treatment has an HLA genetic marker associated with liver injury (e.g., lapatinib, abacavir, amoxicillin/clavulanate) are reviewed and excluded
- Ethics Committee or Institutional Review Board approval of study treatment restart must be obtained, as required.
- If restart of study treatment is approved by GSK Medical Governance in writing, the subject must be provided with a clear description of the possible benefits and risks of study treatment administration, including the possibility of recurrent, more severe liver injury or death.

- The subject must also provide signed informed consent specifically for the study treatment restart. Documentation of informed consent must be recorded in the study chart.
- Study treatment must be administered at the dose specified by GSK.
- Subjects approved by GSK Medical Governance for restarting study treatment must return to the clinic once a week for liver chemistry tests until stable liver chemistries have been demonstrated and then laboratory monitoring may resume as per protocol.
- If after study treatment re-start, subject meets protocol-defined liver chemistry stopping criteria, follow usual stopping criteria instructions.
- GSK Medical Monitor, and the Ethics Committee or Institutional Review Board as required, must be informed of the subject's outcome following study treatment restart.
- GSK to be notified of any adverse events, as per Section 6.3.6.

6.3.4. IgG Stopping Criteria

Any subject who has a Grade 4 IgG level, by the DMID table, will have dosing with study agent withheld, and the appropriateness to continue study treatment must be discussed with Medical Monitor before the next dose. Any subject who has a Grade 4 IgG level associated with a severe or serious infection will have study agent discontinued and will be placed into Part C the safety follow up phase.

6.3.5. Independent Data Monitoring Committee

An IDMC comprised of experts in paediatric rheumatology, paediatric pharmacology, paediatric ethics and a statistician, none of whom are affiliated with the sponsor will be organized to monitor the study as it progresses. A charter will be developed to govern the activities of this committee. The IDMC will conduct its first safety and PK data review after the first cohort has been treated through the Week 8 (Day 56) visit, or within 4 months of the treatment of the first subject enrolled in Cohort 1, whichever is earlier. If the later time point of within 4 months of the first subject enrolled is met, the IDMC will review the available safety data only. The committee will continue to meet, at a minimum, approximately every 6 months from the first meeting until the last subject concludes Part A, the randomized treatment period of the study. After each meeting, the IDMC will generate a report of data reviewed and if applicable, actions taken. Investigators will be notified of the outcome of each IDMC meeting. Notification of local EC/IRB of the IDMC review outcomes will be at the discretion and requirements of local regulations.

If recruitment of subjects to Cohort 2 is slower than expected, the sponsor and the IDMC may decide to initiate an early PK analysis and safety review at 6 months from the first subject's first dose in Cohort 2 being dosed based on the Safety and PK data accrued up to this point from Cohorts 1 and 2. If the PK data analyzed is deemed sufficient, the sponsor and IDMC may recommend a dose adjustment for Cohort 2 subjects from the early PK review. After any early PK review, the sponsor and IDMC may re-review PK

and safety and possibly recommend a dose adjustment when PK data for all Cohort 2 subjects is available. Additionally, the sponsor and IDMC may decide that no additional PK review and dose adjustment is necessary if the data from the early PK review is deemed sufficient to support a finalized PK review.

6.3.6. Adverse Events

The investigator or site staff will be responsible for detecting, documenting and reporting events that meet the definition of an AE or SAE.

6.3.6.1. Definition of an AE

Any untoward medical occurrence in a subject or clinical investigation subject, temporally associated with the use of a medicinal product, whether or not considered related to the medicinal product.

Note: An AE can therefore be any unfavorable and unintended sign (including an abnormal laboratory finding), symptom, or disease (new or exacerbated) temporally associated with the use of a medicinal product. For marketed medicinal products, this also includes failure to produce expected benefits (i.e., lack of efficacy), abuse or misuse.

Events meeting the definition of an AE include:

- Exacerbation of a chronic or intermittent pre-existing condition including either an increase in frequency and/or intensity of the condition
- New conditions detected or diagnosed after study treatment administration even though it may have been present prior to the start of the study
- Signs, symptoms, or the clinical sequelae of a suspected interaction
- Signs, symptoms, or the clinical sequelae of a suspected overdose of either study agent or a concomitant medication (overdose per se will not be reported as an AE/SAE unless this is an intentional overdose taken with possible suicidal/self-harming intent. This should be reported regardless of sequelae).

"Lack of efficacy" or "failure of expected pharmacological action" per se will not be reported as an AE or SAE. However, the signs and symptoms and/or clinical sequelae resulting from lack of efficacy will be reported if they fulfill the definition of an AE or SAE.

Events that **do not** meet the definition of an AE include:

- Medical or surgical procedure (e.g., endoscopy, appendectomy); the condition that leads to the procedure is an AE
- Situations where an untoward medical occurrence did not occur (social and/or convenience admission to a hospital)
- Anticipated day-to-day fluctuations of pre-existing disease(s) or condition(s) present or detected at the start of the study that do not worsen

- The disease/disorder being studied, or expected progression, signs, or symptoms of the disease/disorder being studied, unless more severe than expected for the subject's condition

6.3.6.2. Definition of a SAE

A serious adverse event is any untoward medical occurrence that, at any dose:

a. Results in death

b. Is life-threatening

   NOTE: The term 'life-threatening' in the definition of 'serious' refers to an event in which the subject was at risk of death at the time of the event. It does not refer to an event, which hypothetically might have caused death, if it were more severe.

c. Requires hospitalization or prolongation of existing hospitalization

   NOTE: In general, hospitalization signifies that the subject has been detained (usually involving at least an overnight stay) at the hospital or emergency ward for observation and/or treatment that would not have been appropriate in the physician's office or out-subject setting. Complications that occur during hospitalization are AEs. If a complication prolongs hospitalization or fulfills any other serious criteria, the event is serious. When in doubt as to whether "hospitalization" occurred or was necessary, the AE should be considered serious.

   Hospitalization for elective treatment of a pre-existing condition that did not worsen from baseline is not considered an AE.

d. Results in disability/incapacity,

   NOTE: The term disability means a substantial disruption of a person's ability to conduct normal life functions. This definition is not intended to include experiences of relatively minor medical significance such as uncomplicated headache, nausea, vomiting, diarrhea, influenza, and accidental trauma (e.g. sprained ankle) which may interfere or prevent everyday life functions but do not constitute a substantial disruption.

e. Is a congenital anomaly/birth defect

f. Medical or scientific judgment should be exercised in deciding whether reporting is appropriate in other situations, such as important medical events that may not be immediately life-threatening or result in death or hospitalization but may jeopardize the subject or may require medical or surgical intervention to prevent one of the other outcomes listed in the above definition. These should also be considered serious. Examples of such events are invasive or malignant cancers, intensive treatment in an emergency room or at home for allergic bronchospasm, blood dyscrasias or convulsions that do not result in hospitalization, or development of drug dependency or drug abuse.

g. All events of possible drug-induced liver injury with hyperbilirubinaemia defined as ALT ≥ 3xULN **and** bilirubin ≥ 2xULN (>35% direct) (or ALT ≥ 3xULN and INR>1.5, if INR measured) termed 'Hy's Law' events (INR measurement is not

required and the threshold value stated will not apply to subjects receiving anticoagulants).

NOTE: bilirubin fractionation is performed if testing is available. If testing is unavailable, record presence of detectable urinary bilirubin on dipstick indicating direct bilirubin elevations and suggesting liver injury. If testing is unavailable and a subject meets the criterion of total bilirubin ≥ 2xULN, then the event is still reported as an SAE. If INR is obtained, include values on the SAE form. INR elevations >1.5 suggest severe liver injury.

6.3.7. Laboratory and Other Safety Assessment Abnormalities Reported as AEs and SAEs

Any abnormal laboratory test results (hematology, clinical chemistry, or urinalysis) or other safety assessments (e.g., ECGs, radiological scans, vital signs measurements), including those that worsen from baseline, and felt to be clinically significant in the medical and scientific judgement of the investigator are to be recorded as AEs or SAEs.

However, any clinically significant safety assessments that are associated with the underlying disease, unless judged by the investigator to be more severe than expected for the subject's condition, are **not** to be reported as AEs or SAEs.

6.3.8. Disease-Related Events and/or Disease-Related Outcomes Not Qualifying as SAEs

The following conditions (preferred terms; MedDRA v. 14.0) are disease-related events (DRE) that can occur in the study population regardless of belimumab exposure.

When these conditions are considered SAEs, they must be reported to the sponsor within 24 hours of site personnel becoming aware as described in Section 6.3.14 .However, because these events are typically associated with the disease under study, the sponsor will not submit these events as expedited reports to regulatory authorities, investigators, or IRBs/IECs (unless considered by the sponsor to be related to study agent).

Butterfly rash	Lupus pancreatitis
Cutaneous lupus erythematosus	Lupus pneumonitis
Glomerulonephritis membranoproliferative	Lupus vasculitis
Glomerulonephritis membranous	Nephritic syndrome
Glomerulonephritis proliferative	Nephritis
Lupus encephalitis	Neuropsychiatric lupus
Lupus endocarditis	Pericarditis lupus
Lupus enteritis	Peritonitis lupus
Lupus hepatitis	SLE arthritis
Lupus myocarditis	Systemic lupus erythematosus rash
Lupus nephritis	Systemic lupus erythematosus

Brunner HI, *et al*. *Ann Rheum Dis* 2020; 79:1340–1348. doi: 10.1136/annrheumdis-2020-217101

6.3.9. Suicidality Assessment

Some autoimmune diseases have an increased risk of suicidal behavior and/or ideation (Bachen, 2009, Timonen, 2003, Stenager, 1992). In order to objectively assess suicidality in belimumab clinical programs the C-SSRS will be utilized to collect information on suicidal behavior and ideation.

SLE subjects have an increased prevalence of mood and anxiety disorders compared with the general population and disease activity may contribute to this higher risk. The incidence of major depressive disorder (MDD) among SLE subjects has been reported to be as high as 47% [Bachen, 2009]. Since active SLE and MDD may increase the risk of suicidal ideation or behavior before or during clinical studies, subjects participating in this study will be assessed at every visit for suicidality. Suicidality will be assessed using the Columbia Suicide-Severity Rating Scale (C-SSRS) in children 12 years of age and older in this pediatric SLE study. Children less than 12 years of age will not be assessed with the C-SSRS as the C-SSRS has not been fully validated for use in children less than 12 years of age.

Subjects who answer “yes” to any suicidal behavior or “yes” to suicidal ideation questions 4 or 5 on the C-SSRS should be referred to appropriate psychiatric care and prompts the completion of an SAE worksheet. The medical monitor should be notified when these events occur. In addition, a “yes” to any suicidal behavior or “yes” to suicidal ideation questions 3, 4 or 5 on the C-SSRS prompts the completion of a Possible Suicidality Related Questionnaire (PSRQ) eCRF at all times this condition is met (see PSRQ Section 6.3.10 and PSRQ form in Appendix 13 Possible Suicidality-Related Questionnaire.

Baseline and during treatment assessment of suicidality will be performed in this study using C-SSRS. (Refer to Appendix 12 for C-SSRS).The C-SSRS is a brief measure which is designed to assess severity and change of suicidality by integrating both behavior and ideation [Posner, 2007]. The C-SSRS is administered by a qualified clinician and is designed to address the need for a summary measure to track change in the severity/density of suicidality across both clinical settings and treatment trials. It assesses intensity of ideation (a potentially important marker of severity) by specifically asking about frequency, duration, intrusiveness, controllability, and deterrents. In addition, it captures both the modal and most severe forms of ideation. The C-SSRS is to be completed by the investigator or his/her qualified designee at every visit during the double blind portion of the study.

Although assessment of suicidality using the C-SSRS will take place only during the double blinded portion of the study, investigators are reminded of the importance to clinically assess for suicidality at every visit given that SLE subjects are at increased risk of suicidal behavior and/or ideation.

6.3.10. Possible Suicidality Related Questionnaire (PSRQ)

The PSRQ eCRF form should be completed (Appendix 13 Possible Suicidality-Related Questionnaire) in addition to the AE or SAE pages, as appropriate) if a "yes" response is given to any suicidal behavior or a "yes" response to suicidal ideation questions 3, 4 or 5 on the C-SSRS. If the adverse event meets the definition of an SAE, which includes a "yes" answer to any suicidal behavior or a "yes" to suicidal ideation questions 4 or 5 on the C-SSRS, the site must ensure that there are no significant discrepancies between the PSRQ and SAE.

6.3.11. Investigator Evaluation of Adverse Events

The investigator will evaluate all adverse events with respect to seriousness (criteria for serious are listed in Section 6.3.6.2) severity (intensity) and causality (relationship to study agent). The severity of an AE is to be evaluated according to the Adverse Event Severity Grade Tables in Appendix 5 Clinical Laboratory Tests. If an AE does not have Adverse Event Severity Grade in Appendix 5 Clinical Laboratory Tests, the following severity classifications will be used:

SEVERITY:

Grade 1- Mild - causing no limitation of usual activities (Grade 1 DMID).

Grade 2- Moderate - causing some limitation of usual activities (Grade 2 DMID)

Grade 3- Severe - causing inability to carry out usual activities (Grade 3 or 4 DMID).

An AE that is assessed as severe will not be confused with an SAE. Severity is a category utilized for rating the intensity of an event; and both AEs and SAEs can be assessed as severe. An event is defined as 'serious' when it meets at least one of the pre-defined outcomes as described in the definition of an SAE.

6.3.12. Pregnancy

Any pregnancy that occurs during study participation and up to 16 weeks after last dose must be reported using a clinical trial pregnancy form. To ensure subject safety, each pregnancy must be reported to GSK within 2 weeks of learning of its occurrence. The pregnancy must be followed up to determine outcome (including premature termination) and status of mother and child. Pregnancy complications and elective terminations for medical reasons must be reported as an AE or SAE. Spontaneous miscarriage must be reported as an SAE.

Any SAE occurring in association with a pregnancy brought to the investigator's attention after the subject has completed the study and considered by the investigator as possibly related to the study treatment, must be promptly reported to GSK.

6.3.13. Time Period and Frequency of Detecting AEs and SAEs

The investigator or site staff is responsible for detecting, documenting and reporting events that meet the definition of an AE or SAE. AEs will be collected from the start of study treatment and until the follow up contact at 8 weeks after the last dose of study agent in subjects not entering the open-label (Part B) or safety follow-up phase (Part C), or every 4 weeks (± 7 days) for the first 3 months after entering the safety follow-up phase (Part C). AEs of special interest (AESI, as defined in Section 8.3.5.3.2) will be reported every 6 months for subjects in Part B and annually in subjects entering Part C.

SAEs will be collected over the same time period as stated above for AEs. However, any SAEs assessed **as related** to study participation (e.g., study treatment, protocol-mandated procedures, invasive tests, or change in existing therapy) or related to a GSK concomitant medication, and all AEs of special interest (including serious and non-serious events, as defined in Section 8.3.5.3.2) will be recorded from the time a subject consents to participate in the study up to and including any follow up contact. All SAEs will be reported to GSK within 24 hours, as indicated in Section 6.3.14.

6.3.14. Prompt Reporting of Serious Adverse Events and Other Events to GSK

SAEs and pregnancies meeting pre-defined criteria will be reported promptly by the investigator to GSK as described in the following table once the investigator determines that the event meets the protocol definition for that event.

	**Initial Reports**		**Follow-up Information on a Previous Report**	
**Type of Event**	**Time Frame**	**Documents**	**Time Frame**	**Documents**
All SAEs	24 hours	"SAE" data collection tool	24 hours	Updated "SAE" data collection tool
Pregnancy	2 Weeks	Pregnancy Notification Form	2 Weeks	Pregnancy Follow up Form
**Liver chemistry abnormalities:**				
ALT≥3xULN **and** Bilirubin≥2xULN (>35% direct) (or ALT≥3xULN **and** INR>1.5, if INR measured)***	24 hours*	SAE data collection tool. **Liver Event CRF and liver imaging and/or biopsy CRFs if applicable	24 hours	Updated SAE data collection tool. **Updated Liver Event CRF
ALT≥8xULN; ALT≥5xULN with hepatitis or rash or ≥3xULN and <5xULN that persists ≥4 weeks	24 hours*	**Liver event CRF	24 hours	**Updated Liver Event CRF

	Initial Reports		Follow-up Information on a Previous Report	
**Type of Event**	**Time Frame**	**Documents**	**Time Frame**	**Documents**
ALT≥5xULN plus bilirubin <2xULN	24 hours*	**Liver event CRF does not need completing unless elevations persist for 2 weeks or subject cannot be monitored weekly for 2 weeks	24 hours	
ALT≥5xULN and bilirubin <2xULN that persists ≥2 weeks	24 hours*	**Liver event CRF	24 hours	Updated liver event CRF
ALT≥3xULN and <5x ULN and bilirubin <2xULN	24 hours*	**Liver event CRF does not need completing unless elevations persist for 4 weeks or subject cannot be monitored weekly for 4 weeks		

1. *GSK to be notified at onset of liver chemistry elevations to discuss subject safety.
2. ** Liver event documents should be completed as soon as possible.
3. *** INR measurement is not required; if measured, the threshold value stated will not apply to subjects receiving anticoagulants

Criteria for liver chemistry stopping and follow up criteria can be found in Section 6.3.1.

The method of detecting, recording, evaluating and follow-up of AEs and SAEs plus procedures for completing and transmitting SAE reports to GSK are provided in the SPM. Procedures for post-study AEs/SAEs are provided in the SPM.

6.3.14.1. Regulatory reporting requirements for SAEs

Prompt notification of SAEs by the investigator to GSK is essential so that legal obligations and ethical responsibilities towards the safety of subjects are met.

GSK has a legal responsibility to notify both the local regulatory authority and other regulatory agencies about the safety of a product under clinical investigation. GSK will comply with country specific regulatory requirements relating to safety reporting to the regulatory authority, Institutional Review Board (IRB)/Independent Ethics Committee (IEC) and investigators.

Investigator safety reports are prepared for suspected unexpected serious adverse reactions according to local regulatory requirements and GSK policy and are forwarded to investigators as necessary.

An investigator who receives an investigator safety report describing a SAE(s) or other specific safety information (e.g., summary or listing of SAEs) from GSK will file it with the IB and will notify the IRB/IEC, if appropriate according to local requirements.

6.3.15. Laboratory Evaluations

Clinical laboratory tests will consist of a complete blood count (CBC) with differential, Chem-20, magnesium, and urinalysis (see list in Appendix 4 Liver Safety Criteria). At the discretion of the Investigator, additional samples may be taken for safety reasons. Immunoglobulins, autoantibodies and serum complement (C3 and C4) will also be assessed (see Section 11.5). Samples (blood and urine) for clinical laboratory tests will be collected as described in the Time and Events schedule (Table 2).

At screening, serum human immunodeficiency virus (HIV-1/2) antibody, Hepatitis B (Hepatitis B surface antigen (HBsAg), anti-HBc, anti-HBs), Hepatitis C antibody, prothrombin time (PT) and partial thromboplastin time (PTT) will be evaluated, as well as urine drug screens. Hepatitis B will be assessed on Exit from the study.

All clinical laboratory blood samples will be sent to a central laboratory for analysis (details provided in the SPM). Standard reference ranges will be used. Full details of the collection and shipping requirements for the central laboratory are provided in the Central Laboratory Investigator Manual. The central laboratory will fax laboratory results to the Investigator and will transmit the results electronically to GlaxoSmithKline. To maintain the treatment blind, the site will not be sent information on selected biomarkers from any visits post-randomisation either from the central laboratory or from GSK.

Blood sample collection

The largest volume of blood drawn from a subject occurs at the screening visit and the randomization visit (Day 0). The amount of blood collected at the screening visit and randomization visit (Day 0) may exceed the acceptable volume per country or institution specific guidelines for the pediatric population in this study. Therefore, the volume of blood to be collected should be monitored and blood samples may be drawn in separate visits within 1 week but prior to the scheduled study drug infusion. Alternatively, the blood draw for CRP and other biomarkers may be omitted at the baseline visit. It will be permissible to use the values for these laboratory parameters from the screening visit samples.

6.3.16. Infusion-related Reactions and Hypersensitivity Reactions

In the controlled clinical trials, adverse events associated with the infusion (occurring on the same day of the infusion) were reported in 17% (253/1,458) of subjects receiving belimumab and 15% (99/675) of subjects receiving placebo. Hypersensitivity reactions, including anaphylaxis and death, have been reported in association with belimumab. Delay in the onset of acute hypersensitivity reactions has been observed. Limited data suggest that subjects with a history of multiple drug allergies or significant hypersensitivity may be at increased risk. Investigators are instructed to assume a heightened awareness when screening subjects with a history of adverse reactions to IV

contrast agents, other IV foreign proteins or other monoclonal antibodies; subjects with anaphylaxis to this family of agents should be excluded from enrolment in the study.

Subjects will be monitored during all infusions and should remain under clinical supervision for 3 hours after completion of the first 3 infusions in Part A and for 3 hours after completion of the first 3 infusions in Part B the open label portion of the study according to study sites' guidelines or standard operating procedure for IV infusions. If symptoms of acute hypersensitivity occur, an extended period of monitoring may be appropriate, based on clinical judgment.

Subjects and parents/legal guardian should be made aware of the potential risk, the signs and symptoms of such reactions, and the importance of immediately seeking medical attention.

Premedication with an oral antihistamine, with or without an antipyretic, may be administered before the infusion of belimumab. There is insufficient evidence to determine whether premedication diminishes the frequency or severity of infusion reactions. In the over 15,000 belimumab infusions administered in the Phase III clinical studies, approximately 800 belimumab infusions were administered to subjects who had been premedicated with an antihistamine and antipyretic at the investigator's discretion. In these trials, subjects with a history of allergies were more likely to have been premedicated than subjects without a history of allergies. Although the proportion of infusions with infusion reactions was greater for premedicated infusions than non-premedicated infusions (3% vs <2%, respectively), the incidence of serious and/or severe infusion reactions was 0.1% for non-premedicated infusions while none occurred with premedicated infusions.

Infusion related reactions and/or hypersensitivity reactions will be reported if they fulfill the definition of an AE or SAE.

Delayed-type allergic reactions:

Delayed-type, non-acute hypersensitivity reactions have also been observed and included symptoms such as rash, nausea, fatigue, myalgia, headache, and facial edema.

6.3.17. Immunosuppression, Infections, and Malignancies

Although there is no evidence to date for an increased risk with belimumab treatment, the possibility of immunosuppression resulting in an increase in the frequency and/or severity of infection and/or an increased risk of malignancy cannot be excluded. Therefore, the protocol will exclude subjects who may be at increased risk of infection or malignancy based on medical/medication history and screening physical examination or laboratory findings. During the trial, subjects will be questioned at all study visits about adverse events and results recorded in the eCRF. Examinations and laboratory evaluations will be performed routinely and the results, including markers of potential immunosuppression, will be reported to the investigators.

Progressive Multifocal Leukoencephalopathy

Progressive multifocal leukoencephalopathy (PML) resulting in neurological deficits, including fatal cases, has been reported in SLE subjects receiving immunosuppressant pharmacotherapy, including belimumab. A diagnosis of PML should be considered in any subject presenting with new-onset or deteriorating neurological signs and symptoms. The subject should be referred to a neurologist or other appropriate specialist for evaluation. If PML is confirmed, study agent should be discontinued and consideration should be given to stopping immunosuppressant therapy.

If PML is suspected, this should be immediately reported to the Medical Monitor. The appropriateness of continuing study agent, while the case is being assessed, should be discussed.

6.3.18. Vital Signs, Height and Weight

Systolic and diastolic blood pressure (sitting), heart rate, and body temperature will be measured. Measurements of vital signs, height and weight will be taken for Part A, at screening and prior to dosing on Day 0 and at Weeks 2, 4, every 4 weeks up to week 52/or Exit; and at follow-up visit Week 8 (vital signs only) for subjects who withdraw prior to completion of the 52-week treatment period. At the discretion of the Investigator, vital signs may be assessed at unscheduled visits.

Vital signs, height and weight will be collected at each visit prior to dosing for subject participating in the open label continuation portion of the study (Part B) and annually for those participating in the 10 year post infusion portion of the study (Part C).

6.3.19. Physical Examination

Full physical examinations will be performed at screening. Abbreviated, symptom-driven examinations will be performed for Part A at Day 0 (predose), at every 4 weeks up to Week 52/or Exit; and at follow-up visit Week 8 for subjects who withdraw prior to completion of the 52-week treatment period. At the discretion of the Investigator, physical and neurological examinations may be performed at unscheduled visits.

For subjects participating in the open label continuation portion of the study (Part B), abbreviated, symptom-driven examinations will be performed at every 4 week visit and annually for those participating in the 10 year post infusion portion of the study (Part C).As a minimum complete physical examination will include assessment of the head, eyes, ears, nose, throat, skin, thyroid, neurological, lungs, cardiovascular, abdomen (liver and spleen), lymph nodes, joints, and extremities.

As a minimum brief physical examination will include assessment of the skin, lungs, cardiovascular system, and abdomen (liver and spleen).

6.3.20. Other Safety Outcomes

6.3.20.1. Immunogenicity

Sample Collection and Handling

Serum samples will be collected for belimumab immunogenicity assays prior to dosing at: Day 0, Weeks 8, 24, and 52/or Exit, and at the 8-week follow-up visit for subjects who withdraw prior to completion of the 52-week treatment period and for subjects who choose not to enter the continuation protocol.

At each collection time, 2 mL of blood will be collected in a 2 mL plain tube. Each specimen will be allowed to clot, and the tube then centrifuged at approximately 1,600 g for 15 minutes at room temperature to separate the clot from the serum. The serum (approximately 0.5 mL per sample) will be harvested and placed in 2-mL Sarstedt vials for storage. Serum samples will be stored at -20°C at the sites before weekly batch shipment to the central laboratory. Samples will be stored at -70°C at the central laboratory until shipment to HGS and stored at -80°C after they arrive at HGS. Samples will be shipped on dry ice in both shipments.

The total amount of blood drawn for immunogenicity testing during this study is approximately 10 mL.

Assay Methods

All immunogenicity testing will be carried out at HGS (Rockville, Maryland). HGS has developed a 3-part (screening, confirmation/specificity and neutralisation) belimumab immunogenicity assay paradigm.

In the screening assay, the presence of anti-belimumab antibodies will be assessed using an electrochemiluminescence (ECL)-based (Meso Scale Discovery [MSD]) bridging assay after dissociation of belimumab from the anti-drug antibodies (ADA). Any samples testing positive will then be tested using a confirmatory ECL-based assay which is able to distinguish between ADA (true positive) and BLyS-belimumab complexes in serum (false positive). The confirmed positive samples will be analyzed for the presence of neutralizing antibodies by a neutralisation assay. The results of the anti-belimumab antibody tests for all subjects will be reported at the end of the study.

6.4. Health Outcomes

6.4.1. Pediatric Quality of Life Inventory - Generic Core (PedsQL)

To understand the effect of belimumab on the quality of life in the pediatric lupus population the PedsQL Generic Core will be utilized. The PedsQL is a generic quality of life scale validated for the pediatric population which consists of 23 items, encompassing 4 health domains: Physical Functioning (8 items), Emotional Functioning (5 items), Social Functioning (5 items), and School Functioning (5 items). From the raw scores of the 23 items, a total summary score and individual domain scores can be calculated. The

total and domain scores are each transformed on a 0 to 100 score with higher scores indicating higher quality of life.

The PedsQL measurements will be assessed at screening and prior to dosing at Day 0, Weeks 4, 8, 12, 16, 20, 24, 28, 32, 36, 40, 44, 48, and 52/or Exit and every 6 month in the open label safety follow up (Part B) and every year in the safety follow up phase (Part C). [Brunner, 2009] (See Appendix 14 Pediatric Quality of Life Inventory - Generic Core Scale (PedsQL)).

Subjects ≥ 8 years of age will complete the PedsQL directly. For subjects aged 5-7 years, a parent/guardian will complete the Parent Report version of the PedsQL on their child's behalf.

The PedsQL will only be administered to those subjects for which a validated translation exists in their language.

6.4.2. Pediatric Quality of Life Multidimensional Fatigue Scale

The recently developed 18-item PedsQL Multidimensional Fatigue Scale was designed to measure fatigue in pediatric subjects and comprises the General Fatigue Scale (6 items), Sleep/Rest Fatigue Scale (6 items), and Cognitive Fatigue Scale (6 items). [Varni, 2004], (See Section 11.5, Appendix 15 Pediatric Quality of Life Multidimensional Fatigue Scale (PedsQL Fatigue)).

The PedsQL Fatigue measurements will be assessed at screening and prior to dosing at Day 0, Weeks 4, 8, 12, 20, 24, 36, and 52/or Exit and every 6 month in the open-label safety (Part B) and every year in the safety follow up phase (Part C).

Subjects ≥ 8 years of age will complete the PedsQL-Fatigue directly. For subjects aged 5-7 years, a parent/guardian will complete the Parent Report version of the PedsQL-Fatigue on their child's behalf. The PedsQL-Fatigue will only be administered to those subjects for which a validated translation exists in their language.

6.5. Pharmacokinetics

Blood samples of 0.5 mL will be collected for measurement of serum belimumab concentrations according to schedules which depend on the pediatric cohort.

For Cohort 1 (first 12 children in the study [12-17 years of age]) and Cohort 2 (at least the first 10 children ages 5 to 11 years), samples will be drawn according to the enriched time schedule in Table 3. For all other children in the study (Cohort 3), samples will be drawn according to the standard time schedule in Table 4.

Table 3 PK visit days and sample times for Cohort 1 and Cohort 2

Day (Week)	Time (Related to Dosing of Study agent)
0, 14 (Week 2), 28 (Week 4), and 56 (Week 8)	Before the start of infusion, 5 minutes and 2 hours after the end of the infusion
2±1, 7±1, 16±1, 21±1	Any time during visit
168 (Week 24)	0 - 4 hours after the end of infusion
364/Exit (Week 52)<br>(4 weeks after last dose)	Any time during visit<br>(or before start of infusion if going into the open-label continuation phase of the study)
8-week follow-up<br>(8 weeks after last dose)	Any time during visit<br>(if not going into the open-label continuation phase of the study)

Table 4 PK visit days and sample times for Cohort 3

Day (Week)	Time (Related to Dosing of Study agent)
0	Before the start of infusion and 0-4 hours after the end of infusion
14 (Week 2)	Before the start of infusion and 0 - 4 hours after the end of infusion
28 (Week 4)	Before the start of infusion and 0-4 hours after the end of infusion
56 (Week 8)	Before the start of infusion
168 (Week 24)	0 - 4 hours after the end of infusion
364/Exit (Week 52)<br>(4 weeks after last dose)	Any time during visit<br>(or before start of infusion if going into the open-label continuation phase of the study)
8-week follow-up<br>(8 weeks after last dose)	Any time during visit<br>(if not going into the open-label continuation phase of the study)

On days belimumab is administered and blood samples for pharmacokinetic analysis are obtained, subjects may have an intravenous cannula inserted in the contralateral arm to the arm in which belimumab is administered for laboratory safety assessments and serial pharmacokinetic sampling. Blood samples for pharmacokinetic analysis should not be taken from the same arm as that used for the administration of belimumab. Samples may be obtained by venipuncture.

The total, maximum, amount of blood drawn for PK during this study is approximately 9.5 mL for children in Cohorts 1 and 2 and 5 mL for all other children.

For subjects enrolled in Japan only, additional PK samples (before the start of infusion and 0-4 hours after the end of infusion) will be taken at the 12 week / 3 month and first 6 month visits only in Part B.

Please refer to the Study Procedures Manual for PK sample collection, processing, and shipping instructions.

6.6. Pharmacodynamics

6.6.1. Biomarker(s)

As yet, no clear surrogate endpoints have been widely accepted that clearly define clinical outcomes for the multi-organ system manifestations of SLE. However autoantibodies have been shown to be important early markers of specific disease processes or severity in SLE [Hahn, 1998; Leslie, 2001; Ravirajan, 2001] and are associated with disease activity [Villarreal, 1997]. Therefore autoantibody levels will be included as endpoints.

Blood samples for biomarker evaluations will be collected at the visits specified in the Time and Events schedule (Table 2), to enable the following endpoints to be assessed:

- Baseline values:
  - ANA
  - aCL (IgG, IgM and IgA)
  - BLyS protein
- Percent change from baseline:
  - Total serum immunoglobulin (IgG, IgM and IgA).
  - Autoantibodies (anti-dsDNA) and complement (C3, C4).

Samples will be collected pre-dose where taken at dosing visits. All biomarker samples will be analysed centrally.

Due to the potential for unblinding, the following lab results will not be provided to study sites after Day 0: serum immunoglobulin isotypes IgM/IgA, and B-cells.

6.6.2. Test for Functional Antibodies

Prior history of receiving influenza, diphtheria/tetanus, or pneumococcal vaccines will be obtained from each subject during screening as part of the subject's medical history. A blood sample will be drawn on all subjects for functional antibodies at the Week 0, Week 24 and Week 52 visits. Antibody titer tests will be completed on subjects that plan to receive a vaccine during the treatment period of this study. A blood sample to measure the prevaccination titer will be obtained during a study visit closest to the time prior to the planned vaccination. A post-vaccine titer will be drawn on the next study visit at which other blood is drawn, provided it has been at least 21 days post-immunization. If the immunization was administered less than 21 days prior to a study visit, the post-vaccination titer should be obtained at the following visit.

6.6.3. Vaccine Response

Because of the uncertainty of vaccine response in any immunosuppressed population, it is recommended that all vaccinations be up to date as per country specific guidelines prior to entry into this study. Live vaccinations are not to be administered less than 30 days before first dose or while subjects are receiving study agent.

In addition, consideration should be given to administration of certain vaccines, in particular pneumococcus and meningcoccus, prior to entry if the country specific vaccine schedule would require them soon after study start.

Subjects' immune responses to vaccines received during their participation in the study will be tested. Subjects receiving a primary or a booster dose of influenza, tetanus, or pneumococcal vaccine at anytime during the study may be tested for pre- and post-vaccination antibody titer. Subjects will be asked to inform the investigator if a vaccine is planned. A blood sample to measure the pre-vaccination titer will be obtained preferably prior to the planned immunization. In cases where blood may not be drawn immediately before vaccination, the baseline (Day 0) blood sample will be used as a reference for pre-vaccination titer. A post-vaccination antibody titer will be drawn on the next study visit provided it has been 21 to 60 days from the time the vaccine was given.

6.7. Pharmacogenetics

Information regarding pharmacogenetic research is included in Appendix 1 Pharmacogenetic Research. Participation in this aspect of the study is voluntary. The IEC/IRB and, where required, the applicable regulatory agency must approve the PGx assessments before these can be conducted at the site. The approval(s) must be in writing and will clearly specify approval of the PGx assessments (i.e., approval of Appendix 1 Pharmacogenetic Research). In some cases, approval of the PGx assessments can occur after approval is obtained for the rest of the study. If so, then the written approval will clearly indicate approval of the PGx assessments is being deferred and the study, except for PGx assessments, can be initiated. When PGx assessments will not be approved, then the approval for the rest of the study will clearly indicate this and therefore, PGx assessments will not be conducted.

7. DATA MANAGEMENT

For this study subject data will be entered into GSK defined electronic case report forms (eCRFs), transmitted electronically to GSK and combined with data provided from other sources in a validated data system.

Management of clinical data will be performed in accordance with applicable GSK standards and data cleaning procedures to ensure the integrity of the data, e.g., removing errors and inconsistencies in the data. Adverse events and concomitant medications terms will be coded using MedDRA and an internal validated medication dictionary, GSKDrug. eCRFs (including queries and audit trails) will be retained by GSK, and copies will be sent to the investigator to maintain as the investigator copy. In all cases, subject initials will not be collected or transmitted to GSK according to GSK policy.

8. DATA ANALYSIS AND STATISTICAL CONSIDERATIONS

8.1. Hypotheses

The study is designed to descriptively evaluate the efficacy and safety of belimumab, and as such no formal statistical hypothesis testing is planned.

8.2. Study Design Considerations

8.2.1. Sample Size Considerations

As juvenile SLE is a rare disease, recruitment of subjects will be challenging. The original decision to enrol 100 subjects was based on feasibility estimations. The following is a description of what statistical information these 100 subjects would have provided. Combining the subject cohorts, a total of 100 subjects would have been randomized. In the first two cohorts, at least 24 subjects would have been randomized in a 5:1 ratio (belimumab:placebo), and the remaining 76 subjects would have been randomized in a 1:1 allocation ratio. Therefore, 58 subjects would have been randomized to belimumab and 42 to placebo. Using the methods of PASS 2005 [Hintze, 2006] for the precision of a confidence interval around a single proportion; a sample size of 42 would have produced a 95% confidence interval around the sample proportion ± 0.15191 when the estimated proportion of subjects attaining the primary efficacy response at Week 52 is 0.39 (for placebo), and a sample size of 58 would have produced a 95% confidence interval around the sample proportion ± 0.12793 when the estimated proportion is 0.51 (for belimumab).

8.2.2. Sample Size Sensitivity

The estimated proportions cited above are the Week 52 results for placebo and belimumab 10mg/kg from the combined Phase 3 studies in adults with SLE. The estimated proportions of subjects attaining the primary efficacy response at Week 52 from the 76 week Phase 3 studies in adults with SLE were 34% and 43% for placebo and belimumab 10mg/kg, respectively, and for the Week 52 study in adults with SLE were 43% and 57%.

Sample size sensitivity calculations were performed considering these results. In this study, for estimated proportions ranging from 0.31 to 0.69, the precision for the 95% confidence interval ranges from approximately 0.12 to 0.13 when the sample size is 58, and from approximately 0.14 to 0.15 when the sample size is 42.

8.2.3. Sample Size Re-estimation

As a consequence of continuing enrolment challenges, despite an increase in the the number of participating clinical sites worldwide and concentrated outreach efforts, a sample size reduction based on the extrapolation of current recruitment performance to January 2017 has been agreed to with EMA/PDCO and FDA.

A reduction in subjects from 100 to ‘at least 70’, although affecting the sample size calculations, will not alter the fact that this study was designed to descriptively evaluate the efficacy and safety of belimumab in paediatric SLE.

In the first two cohorts, at least 22 subjects will be randomized in a 5:1 ratio (belimumab:placebo), and the remaining subjects (at least 48) will be randomized in a 1:1 allocation ratio. Therefore, approximately 42 subjects will be randomized to belimumab and 28 to placebo. Using the methods of PASS 2005 [Hintze, 2006] for the precision of a confidence interval around a single proportion; a sample size of 28 will produce a 95% confidence interval around the sample proportion ±0.18143 when the estimated proportion of subjects attaining the primary efficacy response at Week 52 is 0.39 (for placebo), and a sample size of 42 will produce a 95% confidence interval around the sample proportion±0.15286 when the estimated proportion is 0.51 (for belimumab).

Sample size sensitivity calculations were performed considering the primary efficacy response at Week 52 in the Phase 3 studies in adults with SLE. In this study, for estimated proportions ranging from 0.31 to 0.69, the precision for the 95% confidence interval ranges from approximately 0.14 to 0.15 when the sample size is 42 (Belimumab), and from approximately 0.17 to 0.20 when the sample size is 28 (Placebo).

8.3. Data Analysis Considerations

8.3.1. Analysis Populations

The analysis of the double-blind treatment phase will be performed on the intention-to-treat (ITT) population, defined as all subjects who are randomized and treated with at least 1 dose of study agent. The ITT analysis will be performed according to the treatment that a subject was randomized to receive, regardless of the actual treatment received.

The analysis populations for Parts B and C will include all subjects who enter the phase and have at least one assessment.

8.3.2. Analysis Data Sets

The double-blind treatment phase is the primary phase of the study for evaluating the efficacy and safety of the treatments. Data from Part B and Part C will be analyzed separately.

Treatment Failure = Nonresponder (TF=NR)

The TF=NR dataset (treatment failure is described in Section 8.3.5) will be used for the primary response endpoint and each of the 3 components of the primary response. The basic premise of the TF=NR is that a subject who drops out prior to the Week 52 (Section 8.3.5 describes handling of missing data) visit and/or uses a prohibited medication or a non-allowed dose of a restricted medication will be considered a treatment failure and thus counted as a nonresponder in the analysis.

LOCF

The last observation carried forward (LOCF) principle is applied whereby missing values will be replaced with the last previous non-missing value in Part A, B or C. If the first assessment of the treatment phase is missing, then the missing data will be imputed with the baseline value.

Observed

Observed data are the data collected for the subject with no imputation for missing data

8.3.3. Analysis strategy and timing

Following completion of the double-blind treatment phase of the study, this portion of the study database will be locked and the final analysis will be performed. This will not be considered an interim analysis.

Part B and Part C of the study will be summarized separately, and the final analysis performed after completion of each of the phases. Periodic analyses of these two phases may be performed before their completion, but following final analysis of the double-blind phase.

8.3.4. Interim Analysis

IDMC Safety Data Reviews

The IDMC will conduct its first safety and PK data review after Cohort 1 has been treated through the Week 8 (Day 56) visit, or within 4 months of the treatment of the first subject enrolled, whichever is earlier. If the first review occurs at the 4 month time point, then the IDMC will only review safety data. The IDMC will also conduct such a review for Cohort 2. After the initial review, the committee will review the data approximately every 6 months. After each meeting, the IDMC will generate a report of data reviewed and if applicable, recommendations made. Investigators will be notified of the outcome of each IDMC meeting.

The safety data analyses for the IDMC reviews will be performed by an independent statistical analysis data center (SDAC). The PK data analyses will be performed as described below. In addition, GSK GCSP staff may perform unblinding as described in Section 5.4. Otherwise, all personnel having direct responsibility for the conduct of the study, or involved in the belimumab program, will remain blinded to treatment groups for all data until such time that the study is completed by protocol design or the IDMC recommends the termination of the entire study.

PK Interim Data Reviews

Two separate dose assessments will be made by the GSK Safety Review Team based on safety, tolerability and preliminary PK data obtained from Cohorts 1 and Cohorts 2, respectively. The study dose may be revised based upon this review. The Study Team will remain blinded to treatment during these dose assessment meetings, through the use of a PK dataset with scrambled subject identifiers (which may include subject demographics and other PK relevant covariates) prepared by a GSK statistician/ programmer independent to the study team and analyzed by a GSK pharmacokineticist independent to the study team. Decisions regarding dose confirmation/adjustment will be summarized and distributed to study team members, investigators, the IDMC, and IRBs and/or regulatory authorities according to local regulations..

8.3.5. Key Elements of Analysis Plan

This section provides definitions for the primary time point, Week 52.

Treatment Failures

A treatment failure is defined as any subject who

- withdraws from the study prior to Day 364 (Week 52) and has no visit within ± 28 days of Day 364, and/or
- receives a protocol-prohibited medication or a dose of permitted (but protocol-restricted) medication that results in treatment failure designation (see Section 5.6.1 prior to Day 364 (Week 52).

Missing Data Rules

Any subject not otherwise classified as a failure who misses the Day 364 (Week 52) assessments will be handled as follows:

If the subject does not have a visit within ± 28 days of Day 364, the subject will be considered a treatment failure for the Week 52 analysis.

If a subject has at least 1 visit within ± 28 days of Day 364, the data from the visit closest to Day 364 will be used for the Week 52 analysis.

If a subject has 2 visits with equal distance within ± 28 days of Day 364, the data from the visit prior to Day 364 will be used for the Week 52 analysis.

If a subject has a visit within the required window, but partial data of the primary endpoint are missing (including individual items of any component of the primary endpoint), LOCF will be used for the missing item or component. This will be modified for items in which scoring is dependent on both the actual score and the change from the previous visit (BILAG dipstick, BILAG 24 hour urinary protein, BILAG creatinine, and BILAG creatinine clearance). For these items, both the actual value from the last visit and the change observed at that visit will be carried forward.

8.3.5.1. Laboratory Data for Disease Activity Scales

The BILAG and SELENA SLEDAI disease activity scales require laboratory data results for evaluation; therefore, the following procedure will be followed when there are no laboratory data with a sample date that matches the BILAG or SELENA SLEDAI assessment date:

An analysis window will be defined for laboratory data to map values to the BILAG and SELENA SLEDAI assessments.

- For BILAG, the analysis window is -28 days to + 7 days from the BILAG assessment date, excluding the sample that was used in the previous visit assessment.
- For SELENA SLEDAI, the analysis window is -10 days to + 7 days from the SELENA SLEDAI assessment date, excluding the sample that was used in the previous visit assessment.

If there are 2 or more laboratory tests performed within the window, the test with a sample date closest to the disease activity assessment that is prior to the date of assessment will be used. If all tests within the window are after the assessment date, then sample date closest to the assessment date will be used.

If no laboratory data was collected in the window then the last lab test prior to the assessment date will be carried forward.

8.3.5.2. Efficacy Analyses

Since the study is not sized based on statistical power considerations, no p-values will be presented. The data will be summarized using descriptive statistics and confidence intervals. Exact methods may be employed for modelling and confidence intervals, if appropriate for the response rates and sample size. In the event that the overall or within-strata sample sizes are too small to allow for adjustment for randomization stratification factors or other covariates, unadjusted analyses may be performed. The analyses of the primary and major secondary endpoints are described below. Details of analyses for other endpoints including exploratory and sensitivity analyses, will be described in the reporting and analysis plan (RAP).

The primary time point for efficacy analyses will be Week 52. Analysis of the disease activity data collected during Parts B and C of the study will be presented separately and analyzed using descriptive statistics and confidence intervals, using methods similar to the treatment phase.

8.3.5.2.1. Primary Efficacy Endpoint

The primary efficacy endpoint is SLE Responder Index (SRI) response rate at Week 52.

A response is defined as:

≥ 4 point reduction from baseline in SELENA SLEDAI score,

AND

No worsening (increase of < 0.30 points from baseline) in Physician's Global Assessment PGA),

AND

No new BILAG A organ domain score or 2 new BILAG B organ domain scores compared with baseline at the time of assessment (i.e., at Week 52).

The proportion and 95% confidence interval of subjects achieving a response at Week 52 will be presented by treatment group as will the estimated treatment difference and 95% confidence interval. A logistic regression model with main effects for treatment group (belimumab vs. placebo), baseline age group (5-11 vs. 12-17 years), and baseline SLEDAI score group (6-12 vs. ≥ 13) will be run to estimate the odds ratio and 95% confidence interval for belimumab vs. placebo.

Treatment failures and handling of missing data will be managed as described in Section 8.3.5.

As supportive of the primary endpoint, the percentage of subjects meeting each of the three components of the primary endpoint at Week 52 will be presented. To evaluate the response over time, the percentage of subjects achieving a response on the primary endpoint and each of the components of the primary endpoint will be presented by visit.

As a sensitivity analysis or in the event that the overall or within-strata sample sizes are too small to allow for adjustment, a logistic regression model without adjustment for any covariates, and a model with adjustment for baseline SELENA SLEDAI scores ≤ 7 vs. ≥ 8, will be used to analyze the response at Week 52. To examine the robustness of the study results, a LOCF analysis and a completer analysis (i.e., those subjects who complete through Week 52 of the protocol) will also be performed. More details will be described in the statistical analysis plan.

Subgroup Analyses

For the primary endpoint results will be displayed by the randomization stratification subgroups and by complement/anti-dsDNA status at baseline (has low complement [low C3 and/or low C4] AND is anti-dsDNA positive). Other subgroups may be considered if sample size permits.

Brunner HI, *et al. Ann Rheum Dis* 2020; 79:1340–1348. doi: 10.1136/annrheumdis-2020-217101

8.3.5.2.2. *Major Secondary Efficacy Endpoints*

Proportion of subjects meeting PRINTO/ACR Juvenile SLE Response Evaluation criteria for improvement in SLE at Week 52 using two definitions

The five endpoints considered in the PRINTO/ACR Juvenile SLE Response Evaluation definition are percent change in ParentGA, PGA, SELENA SLEDAI score, 24-hour proteinuria and change in PedsQL GC physical functioning domain score at Week 52. The two definitions of PRINTO/ACR Juvenile SLE Response Evaluation responders are:

- PRINTO/ACR Juvenile SLE Response Evaluation 1: At least 50% improvement in 2 of 5 endpoints, with no more than 1 of the remaining worsening by more than 30%
- PRINTO/ACR Juvenile SLE Response Evaluation 2: At least 30% improvement in 3 of 5 endpoints above, with no more than 1 of the remaining worsening by more than 30%

The proportions of subjects (with 95% confidence intervals) meeting PRINTO/ACR Juvenile SLE Response Evaluation criteria (by these two different definitions) at Week 52 will be assessed using a logistic regression model with main effects for treatment group, baseline age group, and baseline SELENA-SLEDAI score group.

Percent change in Parent's Global Assessment (ParentGA) at Week 52

An analysis of covariance (ANCOVA) model will be used to evaluate each treatment group on the percent changes from baseline in ParentGA at Week 52, adjusted for baseline ParentGA score, baseline SELENA SLEDAI score group and age group. The last observation carried forward (LOCF) method will be employed for subjects with missing data on ParentGA score at Week 52. Specifically, if a subject misses the Week 52 visit (± 28 day window allowed), the missing data will be handled by using the last observation available. If a subject takes a protocol-prohibited medication or a dose of allowable medication that results in treatment failure designation prior to Week 52, the data on change from baseline on ParentGA will be handled by using the score from the last visit on or prior to the date that the medication was started.

Percent change in Physician's Global Assessment (PGA) at Week 52

The percent change from baseline to Week 52 in PGA will be analyzed in the same manner as the ParentGA.

Percent change in SELENA-SLEDAI score at Week 52

The percent change from baseline to Week 52 in SELENA SLEDAI score will be analyzed in the same manner as the ParentGA.

Change in PedsQL Physical Functioning Domain Score at Week 52

The changes from baseline in the PedsQL GC physical functioning domain score at Week 52 will be assessed using an analysis of covariance (ANCOVA) model, adjusted for baseline physical functioning domain score, baseline SELENA SLEDAI score group, and

age group. The calculation of the PedsQL physical functioning domain score will be based on the developer instructions [Varni, 2002]. The last observation carried forward (LOCF) method will be employed for subjects with missing data at Week 52. Specifically, if a subject misses the Week 52 visit (± 28 day window allowed), the missing data will be handled by using the last observation available. If a subject takes a protocol-prohibited medication or a dose of allowable (but protocol-restricted) medication that results in treatment failure designation prior to Week 52, the data on change from baseline will be handled by using the score from the last visit on or prior to the date that the medication was started.

Percent change in 24 hour proteinuria at Week 52 (g/24hour equivalent by spot urine protein to creatinine ratio)

The percent change from baseline to Week 52 in 24 hour proteinuria will be analyzed using summary statistics and 95% confidence intervals, without any adjustment for covariates.

Proportion of subjects with a sustained SRI response (defined as having a response on the primary efficacy endpoint at Weeks 44, 48, and 52)

The proportion of subjects with a sustained SRI response will be evaluated by treatment group using a logistic regression model, adjusted for baseline SELENA SLEDAI score group, and age group.

Handling of missing data, including concomitant medication changes resulting in treatment failure designation, will be the same as described for the primary efficacy analysis (Section 8.3.5.2.1).

Proportion of subjects with a sustained ParentGA response (defined as having >0.7 improvement at Weeks 44, 48, and 52 compared at baseline)

The proportion of subjects with a sustained ParentGA response will be analyzed in the same manner as the proportion of subjects with a sustained SRI response. An improvement of 0.7 corresponds to the minimally clinically important difference (MCID) for the instrument using the physician external rating. [Filocamo, 2010]

8.3.5.3. Safety Analyses

Descriptive statistics will be used to summarize adverse events (AEs), changes in laboratory parameters, vital signs and immunogenicity. Unless otherwise specified, safety data will be summarized separately for each study phase (Parts A, B and C).

8.3.5.3.1. Extent of Exposure

Exposure to study agent will be summarized by the number of infusions received and the duration of exposure in days. The total subject-years of exposure will be calculated by treatment group.

8.3.5.3.2. Adverse Events and Disease Related Events

Adverse events and Disease Related Events (DRE) will be coded using MedDRA and summarized by System Organ Classes (SOCs) and Preferred Term. Additionally, AEs will be graded for severity by the investigator using Adverse Event Severity Grading Tables (Appendix 5 Clinical Laboratory Tests). A summary of the number and percentage of subjects with AEs occurring on or after the treatment start date will be displayed by treatment group. Additionally, adverse events will be presented for baseline age stratification subgroups.

The frequency of subjects experiencing an AE will be summarized by:

- All AEs
- Serious AEs
- Severe AEs
- Study Agent Related AEs
- AEs leading to permanent discontinuation of study agent

DRE's will be summarized separately, by SOC, Preferred Term, and treatment group.

Adverse events occurring prior to treatment start date will also be summarized.

The hierarchical relationship between MedDRA, SOCs and Preferred Terms and verbatim text will be displayed for relevant AEs.

Adverse events of special interest are death, malignancies, infusion and hypersensitivity reactions, infections, and suicidality. Separate analyses of these events will be performed as described above for adverse events.

8.3.5.3.3. Laboratory Parameters

The frequency and percent of subjects experiencing a laboratory abnormality will be tabulated by treatment group. Laboratory values will be assessed for significant changes from baseline. For analysis, laboratory toxicity will be graded using Adverse Event Severity Grading tables (Appendix 5 Clinical Laboratory Tests) when possible. Lymphopenia will be graded using Common Terminology Criteria for Adverse Events (CTCAE) Version 4.0 (U.S. Department of Health and Human Services, 2010). Shift tables will be used to determine if subjects move from normal to abnormal (high or low) during the course of the study. Shifts of ≥ 2 grades and Grade 3 or 4 laboratory abnormalities will be summarized.

8.3.5.3.4. Vital Signs

Summary statistics for absolute values and changes from baseline to each visit for each of the vital signs will be presented by treatment group. The number of subjects in each treatment group with vital sign values outside the normal ranges will be tabulated by visit.

8.3.5.3.5. Immunogenicity

Immunogenicity (anti-belimumab antibodies) will be summarized at baseline, and following the start of treatment.

8.3.5.4. Pharmacokinetic Analyses

All randomized subjects will be sampled for serum belimumab levels. A pharmacokinetic (PK) sample will be drawn on Days 0, 14 (Week 2), 28 (Week 4), 56 (Week 8), 168 (Week 24), 364 (Week 52), and at the 8-week follow-up visit (if applicable). For subjects enrolled in Japan only, PK samples will also be taken at the 12 week / 3 month and the first 6 month visits only in Part B. Additional pharmacokinetic samples will be obtained from Cohorts 1 (12 subjects) and 2 (at least the first 10 subjects) for interim pharmacokinetic assessment as specified in the Time and Events Table (Table 2). The PK data for the 10 subjects receiving active treatment that are a part of the first 12 subjects randomized in the 12 and over age group will be reviewed, and dose adjustment may be made based on this review. The same analysis will be performed on the PK data for the subjects on active treatment that are part of the subjects randomized in the 5-11 age group (at least 10 subjects). Population pharmacokinetic analysis is planned, possibly also utilizing adult data.

PK analysis of subjects in Japan will be used to determine the pharmacokinetics of belimumab in pediatric subjects of Japanese descent.

8.3.5.5. Pharmacogenetic Analyses

Any pharmacogenetic analyses will be described in a separate pharmacogenetic analysis plan and will be reported separately from the main clinical study report. All endpoints of interest, all comparisons, will be descriptively and/or graphically summarized as appropriate to the data. Analyses of pharmacogenetic data are described in Appendix 1 Pharmacogenetic Research

8.3.5.6. Biological Markers

Changes from baseline (absolute and percent) and shifts relative to the reference range in collected biomarkers will be evaluated, and the analysis details will be included in the RAP. Pre- and post- vaccination titres will be summarized using descriptive statistics.

9. STUDY CONDUCT CONSIDERATIONS

9.1. Posting of Information on Clinicaltrials.gov

Study information from this protocol will be posted on clinicaltrials.gov before enrolment of subjects begins.

9.2. Regulatory and Ethical Considerations, Including the Informed Consent Process

Prior to initiation of a study site, GSK/HGS will obtain approval from the appropriate regulatory agency to conduct the study in accordance with ICH Good Clinical Practice (GCP) and applicable country-specific regulatory requirements.

The study will be conducted in accordance with all applicable regulatory requirements.

The study will be conducted in accordance with ICH GCP, all applicable subject privacy requirements, and the ethical principles that are outlined in the Declaration of Helsinki 2008, including, but not limited to:

- Institutional Review Board (IRB)/Independent Ethics Committee (IEC) review and approval of study protocol and any subsequent amendments.
- Subject informed consent/assent.
- Investigator reporting requirements.

GSK will provide full details of the above procedures, either verbally, in writing, or both.

Written informed consent must be obtained from each subject's parent(s) or legal guardian prior to the subject's participation in the study. The subject's parent(s) or legal guardian will be re-consented whenever new information becomes available that may affect their willingness for their child to continue to participate in the trial. Assent will be obtained from each pediatric subject in accordance with applicable country or IRB/Ethics Committee regulations. Written informed consent will be obtained from subjects turning 18 years of age to continue participation in the study.

9.3. Quality Control (Study Monitoring)

In accordance with applicable regulations, GCP, and GSK procedures, GSK monitors or those acting on behalf of GSK will contact the site prior to the start of the study to review with the site staff the protocol, study requirements, and their responsibilities to satisfy regulatory, ethical, and GSK requirements. When reviewing data collection procedures, the discussion will include identification, agreement and documentation of data items for which the CRF will serve as the source document.

GSK will monitor the study to ensure that the:

- Data are authentic, accurate, and complete.
- Safety and rights of subjects are being protected.

- Study is conducted in accordance with the currently approved protocol and any other study agreements, GCP, and all applicable regulatory requirements.

The investigator and the head of the medical institution (where applicable) agrees to allow the monitor direct access to all relevant documents.

9.4. Quality Assurance

To ensure compliance with GCP and all applicable regulatory requirements, GSK may conduct a quality assurance audit of the site records, and the regulatory agencies may conduct a regulatory inspection at any time during or after completion of the study. In the event of an audit or inspection, the investigator (and institution) must agree to grant the auditor(s) and inspector(s) direct access to all relevant documents and to allocate their time and the time of their staff to discuss any findings/relevant issues.

9.5. Study and Site Closure

Upon completion or termination of the study, the GSK monitor will conduct site closure activities with the investigator or site staff (as appropriate), in accordance with applicable regulations, GCP, and GSK Standard Operating Procedures.

GSK reserves the right to temporarily suspend or terminate the study at any time for reasons including (but not limited to) safety issues, ethical issues, or severe non-compliance. If GSK determines that such action is required, GSK will discuss the reasons for taking such action with the investigator or head of the medical institution (where applicable). When feasible, GSK will provide advance notice to the investigator or head of the medical institution of the impending action.

If a study is suspended or terminated for **safety reasons**, GSK will promptly inform all investigators, heads of the medical institutions (where applicable),and/or institutions conducting the study. GSK will also promptly inform the relevant regulatory authorities of the suspension/termination along with the reasons for such action. Where required by applicable regulations, the investigator or head of the medical institution must inform the IRB/IEC promptly and provide the reason(s) for the suspension/termination.

9.6. Records Retention

Following closure of the study, the investigator or head of the medical institution (where applicable) must maintain all site study records (except for those required by local regulations to be maintained elsewhere) in a safe and secure location. The records must be easily accessible when needed (e.g., for a GSK audit or regulatory inspection) and must be available for review in conjunction with assessment of the facility, supporting systems, and relevant site staff.

Where permitted by local laws/regulations or institutional policy, some or all of the records may be maintained in a format other than hard copy (e.g., microfiche, scanned, electronic); however, caution must be exercised before such action is taken. The investigator must ensure that all reproductions are legible and are a true and accurate copy of the original. In addition, they must meet accessibility and retrieval standards,

including regeneration of a hard copy, if required. The investigator must also ensure that an acceptable back-up of the reproductions exists and that there is an acceptable quality control procedure in place for creating the reproductions.

GSK will inform the investigator of the time period for retaining the site records in order to comply with all applicable regulatory requirements. The minimum retention time will meet the strictest standard applicable to a particular site, as dictated by local laws/regulations, GSK standard operating procedures, and/or institutional requirements.

The investigator must notify GSK of any changes in the archival arrangements, including, but not limited to archival of records at an off-site facility or transfer of ownership of the records in the event that the investigator is no longer associated with the site.

9.7. Provision of Study Results to Investigators, Posting to the Clinical Trials Register and Publication

Where required by applicable regulatory requirements, an investigator signatory will be identified for the approval of the clinical study report. The investigator will be provided reasonable access to statistical tables, figures, and relevant reports and will have the opportunity to review the complete study results at a GSK site or other mutually-agreeable location.

GSK will also provide the investigators with the full summary of the study results. The investigator is encouraged to share the summary results with the study subjects, as appropriate.

GSK will provide the investigator with the randomization codes for their site only after completion of the full statistical analysis.

The results summary will be posted to the Clinical Study Register at the time of the first regulatory approval or within 12 months of any decision to terminate development. In addition, a manuscript will be submitted to a peer-reviewed journal for publication within 12 months of the first approval or within 12 months of any decision to terminate development. When manuscript publication in a peer-reviewed journal is not feasible, further study information will be posted to the GSK Clinical Study Register to supplement the results summary.

9.8. Independent Data Monitoring Committee (IDMC)

An IDMC, previously described in Section 3.1, will be utilized in this study to ensure external objective medical and/or statistical review of safety and/or efficacy issues in order to protect the ethical and safety interests of subjects and to protect the scientific validity of the study. The schedule of any planned interim analysis and the analysis plan for IDMC review is described in the charter, which is available upon request.

Brunner HI, *et al. Ann Rheum Dis* 2020; 79:1340–1348. doi: 10.1136/annrheumdis-2020-217101

10. REFERENCES

Ad Hoc Working Group on Steroid-Sparing Criteria in Lupus. Criteria for steroid-sparing ability of interventions in systemic lupus erythematosus: report of a consensus meeting. Arthritis Rheum. 2004;50:3427–31.

Bachen E, Chesney M, Criswell L. Prevalence of Mood and Anxiety Disorders inWomen With Systemic Lupus Erythematosus. Arthritis & Rheumatism (Arthritis Care & Research) Vol. 61, No. 6, June 15, 2009, pp 822–829.

Bader Meunier B, Armengaud JB, Haddad E, et al.. Initial Presentation of Childhood-Onset Systemic Lupus Erythemathosus: A French Multicenter Study. J Pediatr. 2005; 146 (5): 648- 653.

Baldassano R, Braegger CP,Escher JC, et al.. Infliximab (REMICADE) Therapy in the Treatment of Pediatric Crohn's Disease, American Journal of Gastroenterology, 2003: 98 (4): 833-838.

Bandeira M et al. Relationship between damage accrual, disease flares and cumulative drug therapies in juvenile-onset systemic lupus erythematosus. Lupus 2006; 15: 515-520.

Barron KS, Silverman ED. Clinical serologic and Immunogenetic studies in childhood-onset Systemic Lupus Erythematosus. Arthritis & Rheumatism 1993;36(3): 348-354

Bombardier C, Gladman DD, Urowitz MB, et al. Derivation of the SLEDAI: a disease activity index for lupus subjects. The Committee on Prognosis Studies in SLE. Arthritis Rheum. 1992;35(6):630-640.

Brocard A, Barbarot S, Milpied B, et al. Thalidomide in the treatment of chronic discoid lupus erythematosus. Ann Dermatol Venereol 2005;132(11 Pt 1):853-6(ISSN:0151-9638).

Brunner HI, Gladman DD, Ibanez D, et al. Difference in disease features between childhood-onset and adult-onset systemic lupus erythematosus. Arthritis Rheum 2008;58(2):556–62.

Brunner HI, Higgins GC, Wiers K, et al. Health-related Quality of Life and Its Relationship to Subject Disease Course in Childhood-onset Systemic Lupus Erythematosus. J Rheumatol 2009;36;1536-1545

Brunner HI, Silverman ED, To T, et al.Risk factors for damage in childhood-onset systemic lupus erythematosus: cumulative disease activity and medication use predict disease damage. Arthritis Rheum 2002;46:436–44.

Buyon JP, Petri MA, Kim MY,et al. The Effect of Combined Estrogen and Progesterone Hormone Replacement Therapy on Disease Activity in Systemic Lupus Erythematosus: A Randomised Trial. Ann Internal Med. 2005;142(12 Pt 1):953-62.

Cevera R, Khamashta MA et al. Systemic Lupus Erythematosus: Clinical and Immunologic patterns of disease expression in a cohort of 1000 subjects. Medicine 1993; 93: 113-123

Chatham WW, Kimberly RP. Treatment of lupus with corticosteroids. Lupus 2001;10(3):140-7.

Cheema DS, Roschke V, Hilbert DM, and Stohl W. Elevated serum B-lymphocyte stimulator levels in subjects with systemic immune-based rheumatic diseases. Arthritis Rheum. 2001;44(6):1313-9.

Cockcroft DW, Gault MH. Prediction of creatinine clearance from serum creatinine. Nephron. 1976;16:31-41

Costallat LT, Coimbra AM. Systemic lupus erythematosus: clinical and laboratory aspects related to age at disease onset. Clin Exp Rheumatol. 1994 Nov-Dec;12(6):603-7.

Dadoniene J et al. The prevalence of systemic lupus erythematosus in Lithuania: the lowest rate in Northern Europe. Lupus 2006; 15: 544-546.

Eibl MM, Rosen FS. Intravenous gamma globulin and plasmapheresis. In: Frank MM, editor. Samter's Immunologic Diseases (5th ed.). Boston:Little, Brown, 1995:1529-36.

El-Hallak M, Binstadt BA, Leichtner AM, et al. Clinical effects and safety of rituximab for treatment of refractory paediatric autoimmune diseases. J Pediatr. 2007 Apr;150(4):376-82.

Filocamo et al. Evaluation of 21-Numbered Circle and 10-Centimeter Horizontal Line Visual Analog Scales for Physician and Parent Subjective Ratings in Juvenile Idiopathic Arthritis. J Rheumatol 2010;37:1534–41

Fish AJ, Blau EB et al. Systemic Lupus Erythematosus within the first two decades of life. The American Journal of Medicine 1977; 62:99-117

Ginsberg JM, Chang BS, Matarese RA, Garella S. Use of single voided urine samples to estimate quantitative proteinuria. N Engl J Med. 1983;309(25):1543-6.

Gladman EM, Ginzler E, Goldsmith C, et al. The development and initial validation of the systemic lupus international collaborating clinics/American college of rheumatology damage index for systemic lupus erythematosus. Arthritis Rheum. 1996;39(3):363-9.

Goldfarb NS, Avery RK, Goormastic M, et al. Hypogammaglobulinemia in lung transplat recipients. Transplantation. 2001;71(2):242-6.

Gordon C, Sutcliffe N, Skan J, Stoll T, Isenberg DA. Definition and treatment of lupus flares measured by the BILAG index. Rheumatology (Oxford). 2003;42(11):1372-9.

Gourley IS, Patterson CC and Bell AL. The Prevalence of Systemic Lupus Erythematosus in Northern Ireland. Lupus 1997; 6: 399-403.

Gudmundsson S and Steinsson K. Systematic Erythematosus in Iceland 1975 through 1984. A Nationwide Epidemiological Study in an Unselected Population. The Journal of Rheumatology 1990; 17:9 1162-1167.

Gutiérrez Suárez R, Ruperto N, Gastaldi R, et al. A proposal for a pediatric version of the Systemic Lupus International Collaborating Clinics/American College of Rheumatology Damage Index based on the analysis of 1,015 subjects with juvenile-onset systemic lupus erythematosus. Arthritis and Rheumatism 2006;54:2989-2996

Hahn BH. Antibodies to DNA. N Engl J Med. 1998;338(19):1359-68.

Härtel C, Adam N, Strunk T, Temming P, Müller-Steinhardt M, Schultz C. Cytokine responses correlate differentially with age in infancy and early childhood. Clin Exp Immunol. 2005 Dec;142(3):446-53.

Hay EM, Bacon PA, Gordon C, et al. The BILAG index: a reliable and valid instrument for measuring clinical disease activity in systemic lupus erythematosus. Q J Med. 1993;86(7):447-58.

Hintze, J. (2006) NCSS, PASS, and GESS. NCSS. Kaysville, Utah. WWW.NCSS.COM.

Hochberg MC. Updating the American College of Rheumatology revised criteria for the classification of systemic lupus erythematosus. Arthritis Rheum. 1997;40(9):1725.

Hong SD, Reiff A, Yang H, et al. B Lymphocyte Stimulator Expression in Pediatric Systemic Lupus Erythematosus and Juvenile Idiopathic Arthritis Subjects, Arthritis & Rheum. 2009; 60(11): 3400–3409

Hopkinson ND, Doherty M and Powell RJ. The Prevalence and Incidence of Systemic Lupus Erythematosus in Nottingham, UK 1989-1990. British Journal of - Rheumatology 1993; 32: 110-115.

Houssiau FA, Vasconcelos C, D'Cruz D et al. Early Response to Immunosuppressive therapy predicts good renal outcome in lupus nephritis. Arthrhritis Rheum 2004;50(12):3934-40.

Isenberg DA, Gordon C. From BILAG to BLIPS--disease activity assessment in lupus past, present and future. Lupus. 2000;9(9):651-4.

James LP, Letzig L, Simpson PM, Capparelli E, Roberts DW, Hinson JA, Davern TJ, Lee WM. Pharmacokinetics of Acetaminophen-Adduct in Adults with Acetaminophen Overdose and Acute Liver Failure. Drug Metab Dispos 2009; 37:1779-1784.

Jimenez S, Cevera R et al. The epidemiology of SLE. Clinical reviews in Allergy and Immunology 2003; 25: 3-11

KDOQI Clinical Practice Guidelines for Chronic Kidney Disease: evaluation, classification, and stratification. Part 5. Evaluation of laboratory measurements for clinical assessment of kidney disease. Guideline 5. Assessment of proteinuria. National

Kidney Foundation, 2002. Retrieved 2 February 2006 from http://www.kidney.org/professionals/kdoqi/guidelines_ckd/p5_lab_g5.htm

King KK, Kornreich HK. The clinical spectrum of SLE in childhood 1977: 287-294

Kovarik JM, Offner G, Broyer M, Niaudet P, Loirat C,Mentser M, Lemire J, Crocker JF, Cochat P, Clark G, Gerbeau C, Chodoff L, Korn A, Hall M A rational dosing algorithm for basiliximab (Simulect) in pediatric renal transplantation based on pharmacokinetic-dynamic evaluations. Transplantation 2002;74(7):966–971.

Le Gal F, Gordien E, Affolabi D, Hanslik T, Alloui C, Dény P, Gault E. Quantification of Hepatitis Delta Virus RNA in Serum by Consensus Real-Time PCR Indicates Different Patterns of Virological Response to Interferon Therapy in Chronically Infected Subjects. J Clin Microbiol. 2005;43(5):2363–2369.

Lehman TJ, McCurdy DK, Bernstein et al. Systemic Lupus Erythematosus in the first decade of life. Pediatrics 1989; 83:235.

Lehman TJ. Paediatric rheumatology in the 21st century--where's the common sense? J Rheumatol. 1997 Sep;24(9):1855-6.

Lehman TJ. The future of paediatric rheumatology. Many questions remain. Arthritis and Rheumatism 2007; 56(9) 2815-2816.

Leslie D, Lipsky P, Notkins AL. Autoantibodies as predictors of disease. J Clin Invest. 2001;108(10):1417-22.

Lopez P et al. Epidemiology of systemic lupus erythematosus in a northern Spanish population: gender and age influence on immunological features. Lupus 2003; 12:860-865.

Marks SD and Tullus K. Targeted B cell depletion therapy. Paediatric drugs 2007; 9(6): 371-378

Naleway AL, Davis ME, Greenlee RT, Wilson DA, McCarty DJ. Epidemiology of systemic lupus Erythematosus in rural Wisconsin. Lupus 2005; 14:862-866.

National Women's Health Information Center (NWHIC), 2003; US Department of Health and Human Services. Available on-line at www.hhs.gov.

Nightingale AL, Farmer RDT, de Vries CS. Systemic lupus erythematosus prevalence in the UK: methodological issues when using the General Practice Research Database to estimate frequency of chronic relapsing-remitting disease. Pharmacoepidemiology and Drug Safety 2007; 16: 144-151.

Nossent HC. Systematic Lupus Erythematosus in the Arctic Region of Norway. The Journal of Rheumatology 2001.

Pescovitz MD, Knechtle S, Alexander SR, et al. Safety and pharmacokinetics of daclizumab in pediatric renal transplant recipients. Pediatr Transplant 2008 Jun; 12 (4): 447-55.

Petri M, Buyon J, Kim M. Classification and definition of major flares in SLE clinical trials. Lupus. 1999;8:685-91.

Petri M, Kim MY, Kalunian KC, Grossman J, Hahn BH, Sammaritano LR, Lockshin M, Merrill JT, Belmont HM, Askanase AD, McCune WJ, Hearth-Holmes M, Dooley MA, Von Feldt J, Friedman A, Tan M, Davis J, Cronin M, Diamond B, Mackay M, Sigler L, Fillius M, Rupel A, Licciardi F, Buyon JP. Combined oral contraceptives in women with systemic lupus erythematosus. N Engl J Med. 2005;353:2550-8.

Petri M, Stohl W. Chatham W, et al. Association of plasma B-Lymphocyte stimulator (BLyS) levels and disease activity in systemic lupus erythematosus. Arthritis Rheum 2008; 58(8):2453-9.

Petri M. Systemic lupus erythematosus: clinical aspects. In: Koopman W, editor. Arthritis and Allied Conditions: A Textbook of Rheumatology. 14th ed. Philadelphia, PA:Lippincott Williams and Wilkins, 2001:1455-79.

Petty RE, Laxer RM (2005) Systemic lupus erythematosus. In: Cassidy JT, Petty RE, Laxer RM, Lindsey CB (redacteuren) Textbook of Pediatric Rheumatology. Amsterdam: Elsevier Saunders, hoofdstuk 16, pp 342-385

Pisetsky DS. Systemic lupus erythematosus: epidemiology, pathology, and pathogenesis. In: Primer on the Rheumatic Diseases. Arthritis Foundation, Atlanta 2001;17:329-35.

Pluchinotta FR, Schiavo B, Vittadello F, Martini G, Perilongo G, Zulian F. Distinctive clinical features of paediatric systemic lupus erythematosus in three different age classes. Lupus. 2007;16(8):550-5.

Podolskaya A, Stadermann M, Pilkington C, Marks SD, Tullus K. B cell depletion therapy for 19 subjects with refractory systemic lupus erythematosus. Arch Dis Child. 2008 May;93(5):401-6. Epub 2007 Nov 26.

Posner K, Oquendo MA, Gould M, Stanley, Davies M**.** Columbia Classification Algorithm of Suicide Assessment (C-CASA): Classification of Suicidal Events in the FDA's Pediatric Suicidal Risk Analysis of Antidepressants**.** Am J Psychiatry 2007; 164:1035–1043).

Press J. Palagew K. antiribosomal antibodies. Arthritis and Rheumatism 1996: 671-676

Price CP, Newall RG, Boyd JC. Use of Protein: Creatinine Ratio measurements on random urine samples for prediction of significant proteinuria: A Systematic Review. Clinical Chemistry. 2005;51(9):1-11.

Ravirajan CT, Rowse L, MacGowan JR, Isenberg DA. An analysis of clinical disease activity and nephritis-associated serum autoantibody profiles in subjects with systemic lupus erythematosus: a crosssectional study. Rheumatology. 2001;40(12):1405-12

Reichlin M, Wolfson-Reichlin M. Evidence for the participation of anti-ribosomal P antibodies in lupus nephritis. Arthritis Rheum. 1999 Dec;42(12):2728-9.

Reveille JD. The treatment of systemic lupus erythematosus. In: Koopman W, ed. Arthritis and Allied Conditions: Textbook of Rheumatology. 14th ed. Philadelphia, PA: Lippincott Williams and Wilkins 2001;1533-45.

Ruggenenti P, Gaspari F, Perna A, Remuzzi G. Cross sectional longitudinal study of spot morning urine protein: creatinine ratio, 24 hour urine protein excretion rate, glomerular filtration rate, and end stage renal failure in chronic renal disease in subjects without Diabetes. Erratum in BMJ 1998;317(7171):1491. BMJ. 1998;316(7130):504-9.

Ruiz-Irastorza G, Khamashta M, Castellino G, et al. Systemic lupus erythematosus. Lancet 2001; 357(9261):1027-32.

Ruperto et al. for the Pædiatric Rheumatology International Trials Organisation (PRINTO) and the Pediatric Rheumatology Collaborative Study Group (PRCSG). The Pediatric Rheumatology International Trials Organization/American College of Rheumatology provisional criteria for the evaluation of response to therapy in juvenile systemic lupus erythematosus. Prospective validation of the definition of improvement. . Arthritis Rheum*,* 2006;55:355-363.

Ruperto et al. for the Pediatric Rheumatology International Trials Organisation (PRINTO) and the Pediatric Rheumatology Collaborative Study Group (PRCSG). The Pediatric Rheumatology International Trials Organisation criteria for the evaluation of response to therapy in juvenile systemic lupus erythematosus. Prospective validation of the core set. Arthritis Rheum 2005;52(9):2854-2864.

Ruperto N, Martini A. International research networks in pediatric rheumatology: the PRINTO/ACR perspective. Curr Opin Rheumatol 2004;16:566–70.

Schwartz G et al. New Equations to Estimate GFR in Children with CKD. J Am Soc Nephrol 2009; 20:629-637.

Singsen DH. Rheumatic diseases of childhood epidemiology of Rheumatic disease 1998; 16(3): 581-599.

Somers EC et al. Incidence of Systemic Lupus erythematosus in the United Kingdom, 1990-1999. Arthritis & Rheumatism (Arthritis Care and Research) 2007; 57: 601-618.

Stenager EM, Stenager E, Koch-Henriksen N, Br0nnum-Hansen H, Hyllested K, Jensen K, Bille-Brahe U. Suicide and multiple sclerosis: an epidemiological investigation Journalof Neurology, Neurosurgery, and Psychiatry 1992;55:542-545

Stiehm ER, Fletcher CV, Mofenson LM, Palumbo PE, Kang M. Fenton T. Sapan CV, Meyer III WA, Shearer WT, Hawkins E, Fowler MG, Bouquin P, Purdue L, Sloand EM, Nemo GJ, Wara D, Bryson YJ, Starr SE, Petru A, Burchett, for the Pediatric AIDS Trials Group Protocol 273 Study Group. Use of human immunodeficiency virus (HIV) human hyperimmune immunoglobulin in HIV type 1-infected children The Journal of Infectious Diseases 2000;181:548-554

Stoll T, Seifert B, Idenberg DA. SLICC/ACR Damage Index is valid, and renal and pulmonary organ scores are predictors of severe outcome in subjects with Systemic Lupus Erythematosus. Brit. J. Rheumatology. 1996;35(3):248-254.

Tan EM, Cohen AS, Fries JF, Masi AT, McShane DJ, Rothfield NF, Schaller JG, Talal N, Winchester RJ. The 1982 revised criteria for the classification of systemic lupus erythematosus. Arthritis Rheum. 1982;25(11):1271-7.

Timonen M, Viilo K, Hakko H, Sa¨rkioja T, Ylikulju M, Meyer-Rochow VB, Va¨isa¨nen E, Ra¨sa¨nen P. Suicides in persons suffering from rheumatoid. Arthritis. Rheumatology 2003;42:287–291.

Traggiai E, Volpi S, Schena F, Gattorno M, Ferlito F, Moretta L, Martini A. Bone marrow-derived mesenchymal stem cells induce both polyclonal expansion and differentiation of B cells isolated from healthy donors and systemic lupus erythematosus subjects. Stem Cells. 2008 Feb;26(2):562-9.

Tucker LB, Uribe AG, Fernandez M, et al. Adolescent onset of lupus results in more aggressive disease and worse outcomes: results of a nested matched case-control study within LUMINA, a multiethnic US cohort (LUMINA LVII). Lupus 2008;17(4):314–22.

Tucker LB. Adult and childhood SLE. British Journal of Rheumatology 1995; 34; 866-872

U.S. Department of Health and Human Services, National Institutes of Health, National Cancer Institute. Common Terminology Criteria for Adverse Events (CTCAE) Version 4.0 Published: May 28, 2009 (v4.03: June 14, 2010).

Uziel Y et al. Outcome of a national Israeli cohort of paediatric systemic lupus erythematosus. Lupus 2007; 16: 142-146.

Varni J, Burwinkle T, Szeri I. The PedsQL Multidimensional Fatigue Scale in Pediatric Rheumatology: Reliability and Validity. The Journal of Rheumatology 2004; 31:12:2495-2500.

Varni, J, et al. The PedsQL™ in Pediatric Rheumatology: Reliability, Validity, and Responsiveness of the Pediatric Quality of Life Inventory™ Generic Core Scales and Rheumatology Module. Arthriris & Rheumatism, Vol. 46, No. 3, March 2002, pp 714–725.

Villarreal GM, Drenkard C, Villa AR, Slor H, Shafrir S, Bakimer R, Shoenfeld Y, Alarcón-Segovia D. Prevalence of 13 autoantibodies and of the 16/6 and related

pathogenic idiotypes in 465 subjects with systemic lupus erythematosus and their relationship with disease activity. Lupus. 1997;6:425-35.

Wallace DJ. Management of lupus erythematosus: recent insigts. Current Opinions in Rheumetology 2002;14:212-9.

Wang W, Wang EQ, Balthasar JP. Monoclonal antibody pharmacokinetics and pharmacodynamics. Clin Pharmacol Ther 2008;84(5):548-58.

Yamini MH, Avery RK, Mawhorter SD, Young JB, Ratliff NB, Hobbs RE, McCarthy PM, Smedira NG, Goormastic M, Pelegrin D, Starling RC. Hypogammaglobulinemia following cardiac transplantation: a link between rejection and infection. J Heart Lung Transplant. 2001;20(4):425-30.

11. APPENDICES

11.1. Appendix 1: Pharmacogenetic Research

Pharmacogenetics – Background

Pharmacogenetics (PGx) is the study of variability in drug response due to hereditary factors in different populations. There is increasing evidence that an individual's genetic composition (i.e., genotype) may impact the pharmacokinetics (absorption, distribution, metabolism, elimination), pharmacodynamics (relationship between concentrations and pharmacologic effects or the time course of pharmacologic effects) and/or clinical outcome (in terms of efficacy and/or safety and tolerability). Some reported examples of PGx analysis include:

Drug	Disease	Gene	Outcome
Abacavir	HIV [Hetherington, 2002; Mallal, 2002]	HLA –B*5701	Individuals with HLA-B*5701 variant may be at increased risk for experiencing hypersensitivity to abacavir. Clinical assays are available for HLA-B*5701 but none has been validated. HLA-B*5701 screening would supplement but never replace abacavir clinical risk management strategies aimed at minimising rare but serious outcomes associated with abacavir hypersensitivity.
Warfarin	Cardiovascular [Neergard, 2006; Wilke, 2005]	CYP2C9	Serious adverse events (SAEs) experienced by some subjects on warfarin may be explained by variations in the CYP2C9 gene that influences the degree of anticoagulation achieved.
Irinotecan	Cancer [FDA News Release, 2005]	UGT1A1	Variations in the UGT1A1 gene can influence a subject's ability to break down irinotecan, which can lead to increased blood levels of the drug and a higher risk of side effects. A dose of irinotecan that is safe for one subject with a particular UGT1A1 gene variation, might be too high for another subject without this variation, raising the risk of certain side-effects.<br>A genetic blood test (Invader UGT1A1 molecular assay) is available that can detect variations in the gene.

A key component to successful PGx research is the collection of samples during the conduct of clinical studies.

Collection of whole blood samples, even when no *a priori* hypothesis has been identified, may enable PGx analysis to be conducted if at any time it appears that there is a potential unexpected or unexplained variation in handling or response to belimumab.

Pharmacogenetic Research Objectives

The objective of the PGx research (if there is a potential unexpected or unexplained variation) is to investigate a possible genetic relationship to handling or response to belimumab. If it appears there is potential variability in response in this clinical study or in a series of clinical studies with belimumab that may be attributable to genetic variations of subjects, the following objectives may be investigated:

- Relationship between genetic variants and the pharmacokinetics and/or pharmacodynamics of study treatment
- Relationship between genetic variants and safety and/or tolerability of study treatment
- Relationship between genetic variants and efficacy of study treatment

Study Population

Any subject who has given informed consent to participate in the clinical study, has met all the entry criteria for the clinical study, and receives study treatment may take part in the PGx research. Any subject who has received an allogeneic bone marrow transplant must be excluded from the PGx research.

Subject participation in the PGx research is voluntary and refusal to participate will not indicate withdrawal from the clinical study. Refusal to participate will involve no penalty or loss of benefits to which the subject would otherwise be entitled.

Study Assessments and Procedures

As saliva is being collected for analysis, no additional whole blood samples will be necessary for the PGx analysis. Deoxyribonucleic acid (DNA) will be extracted from cells obtained from the subject’s saliva. Saliva (2mL) is spit into the DNA self-collection kit. A single sample will be taken but can be duplicated if the first sample is unusable. It is recommended that the saliva sample be collected at Day 0/Baseline visit.

The PGx sample is labelled (or “coded”) with a study specific number that can be traced or linked back to the subject by the investigator or site staff. Coded samples do not carry personal identifiers (such as name or social security number). The saliva sample is taken on a single occasion unless a duplicate sample is required due to inability to utilize the original sample.

The DNA extracted from the saliva sample may be subjected to sample quality control analysis. This analysis will involve the genotyping of several genetic markers to confirm the integrity of individual samples. If inconsistencies are noted in the analysis, then those samples may be destroyed.

The need to conduct PGx analysis may be identified after a study (or a set of studies) of belimumab has been completed and the study data reviewed.

In some cases, the samples may not be studied. e.g., no questions are raised about how people respond to belimumab.

Samples will be stored securely and may be kept for up to 15 years after the last subject completes the study or GSK may destroy the samples sooner. GSK or those working with GSK (for example, other researchers) will use samples collected from the study for the purpose stated in this protocol and in the informed consent form.

Subjects can request their sample to be destroyed at any time.

Subject Withdrawal from Study

If a subject who has consented to participate in PGx research and has a sample taken for PGx research withdraws from the clinical study for any reason other than lost to follow-up, the subject will be given the following options:

- The sample is retained for PGx research
- Any PGx sample is destroyed.

If a subject withdraws consent from the PGx research or requests sample destruction for any reason, the investigator must complete the appropriate documentation to request sample destruction within the timeframe specified by GSK and maintain the documentation in the site study records. If the sample has already been processed, it will be destroyed after all steps are complete. GSK will ensure that any data related to the sample will not be analysed. The sample will be destroyed after processing is complete.

Screen and Baseline Failures

If a saliva sample for PGx research has been collected and it is determined that the subject does not meet the entry criteria for participation in the clinical study, then the investigator must complete the appropriate documentation to request sample destruction within 5 days. The sample will be destroyed and documentation sent to the site within 30 working days of receipt of the request for destruction. All documents pertaining to sample destruction must be maintained in the site study records.

Pharmacogenetics Analyses

1. Specific sections of DNA may be selected from areas of the genome (e.g., candidate genes) known to encode the drug target, drug metabolizing enzymes, areas associated with mechanisms underlying adverse events, and those linked to study disease and, thus, linked to drug response.

The candidate genes that may be investigated in this study include genes that code for enzymes, transporters, proteins or receptors that may be involved in response to belimumab.

2. By evaluating large numbers of polymorphic markers (e.g., single nucleotide polymorphisms or SNPs) throughout the genome, sets of markers may be identified that correspond to differential drug response.

The results of PGx investigations will be reported either as part of the main clinical study report or as a separate report. All endpoints of interest from all comparisons will be descriptively and/or graphically summarised as appropriate to the data. In all cases,

appropriate statistical methods will be used to analyse the genetic markers in the context of other clinical data. Statistical methods may include, but are not limited to Hardy-Weinberg Equilibrium testing, Comparison of Demographic and Baseline Characteristics by Genotype, Evaluation of Genotypic Effects, Evaluation of Treatment by Genotype and Gene-Gene Interaction, Linkage Disequilibrium, Multiple Comparison and Multiplicity and/or Power and Sample Size Considerations. Detailed description of the analyses to be conducted will be documented in the Pharmacogenetics Reporting and Analysis Plan.

3. Genome-wide scans involving a large number of polymorphic markers (e.g., single nucleotide polymorphisms) located throughout the genome. This approach is often employed when potential genetic effects are not well understood.

Informed Consent/Assent

Subjects who do not wish to participate in the PGx research may still participate in the clinical study. PGx informed consent must be obtained prior to any saliva being taken for PGx research.

Provision of Study Results and Confidentiality of Subject's PGx Data

GSK may summarize the cumulative PGx research results in the clinical study report.

In general, GSK does not inform the investigator, subject, or anyone else (e.g., family members, study investigators, primary care physicians, insurers, or employers) of the PGx research results that are not known to be relevant to the subject's medical care at the time of the study, because the information generated from PGx studies is preliminary in nature, and the significance and scientific validity of the results are undetermined at such an early stage of research, under any circumstances unless required by law.

References

Hetherington S, Hughes AR, Mosteller M, Shortino D, Baker KL, Spreen W, Lai E, Davies K, Handley A, Dow DJ, Fling ME, Stocum M, Bowman C, Thurmond LM, Roses AD. Genetic variations in HLA-B region and hypersensitivity reactions to abacavir. Lancet. 2002; 359:1121-2.

Mallal S, Nolan D, Witt C, Masel G, Martin AM, Moore C, Sayer D, Castley A, Mamotte C, Maxwell D, James I. Association between presence of HLA-B*5701, HLA-DR7, and HLA-DQ3 and hypersensitivity to HIV-1 reverse-transcriptase inhibitor abacavir. Lancet. 2002; 359:727-32.

Neergard. Reducing the risk of blood thinners. Associated press, September 2006.

U.S. Food and Drug Administration, FDA Clears Genetic Test That Advances Personalized Medicine Test Helps Determine Safety of Drug Therapy 22 August 2005, http://www.fda.gov/bbs/topics/NEWS/2005/NEW01220.html.

Wilke RA, Musana AK, Weber WW. Cytochrome P450 gene-based drug prescribing, and factors impacting translation into routine clinical practice. Personalized Med 2005; 2: 213-224.

11.2. Appendix 2: Country Specific Requirements

Information pending determination of country participation.

Study Inclusion Criterion – age of subjects

At sites in the Russian Federation, only subjects ≥ 12 years of age will be eligible to participate.

Study Inclusion Criterion specific for sites in the Russian Federation:

Subjects eligible for enrolment in the study must be ≥ 12 years of age to participate.

Study Treatment Restart per Section 6.3.3

At sites in the Russian Federation, restart of study treatment after liver event is not permitted.

11.3. Appendix 3: American College of Rheumatology (ACR) Criteria for SLE

The ACR Criteria for the Classification of Systemic Lupus Erythematosus* [Tan, 1982; Hochberg, 1997]

Criterion	Definition
1. Malar "butterfly" rash	Fixed erythema, flat or raised, over the malar eminences, tending to spare the nasolabial folds.
2. Discoid rash	Erythematosus raised patches with adherent keratotic scaling and follicular plugging; atrophic scarring may occur in older lesions.
3. Photosensitivity	Skin rash as a result of unusual reaction to sunlight, by subject history or physician observation.
4. Oral ulcers	Oral or nasopharyngeal ulceration usually painless.
5. Arthritis	Nonerosive arthritis involving 2 or more peripheral joints characterized by tenderness.
6. Serositis	a. Pleuritis (convincing history or pleuritic pain or rub heard by physician or evidence of pleural effusion), *OR*<br>b. Pericarditis (documented by ECG, rub, or evidence or pericardial effusion).
7. Renal disorder	a. Persistent proteinuria (> 0.5 grams/day or > 3 + if quantitation not performed) ***OR***<br>b. Cellular casts (may be red cell, hemoglobin, granular, tubular, or mixed).
8. Neurologic disorder	a. Seizures (in the absence of offending drugs or known metabolic derangements; ie, uremia, ketoacidosis, or electrolyte imbalance) *OR*<br>b. Psychosis (in the absence of offending drugs or known metabolic derangements; ie, uremia, ketoacidosis, or electrolyte imbalance.
9. Hematologic disorder	a. Hemolytic anemia (with reticulocytosis)<br>***OR***<br>b. Leukopenia (< 4000/mL total on 2 or more occasions), *OR*<br>c. Lymphopenia (< 1500/mL on 2 or more occasions), *OR*<br>d. Thrombocytopenia (< 100,000/mL in the absence of offending drugs).
10. Immunologic disorder	a. Anti-DNA (antibody to native DNA in abnormal titer), *OR*<br>b. Anti-Sm (presence of antibody to Sm nuclear antigen), *OR*<br>c. Positive-finding of antiphospholipid antibodies based on 1) an abnormal serum level of IgG or IgM anticardiolipin antibodies, 2) a positive test result for lupus anticoagulant using a standard method, or 3) a false-positive serologic test for syphilis known to be positive for at least 6 months and confirmed by *Treponema pallidum* immobilization (TPI) or fluorescent treponemal antibody (FTA) absorption test.
11. Antinuclear antibody (ANA)	Abnormal titer of ANA by immunofluorescence or an equivalent assay at any point in time and in the absence of drugs known to be associated with "drug-induced lupus" syndrome.

1. * The proposed classification is based on 11 criteria. For the purpose of identifying subjects in clinical studies, a person shall be said to have systemic lupus erythematosus if any 4 or more of the 11 criteria are present, serially or simultaneously, during any interval or observation.

11.4. Appendix 4: Liver Safety Criteria

11.4.1. Liver Stopping Event Algorithm

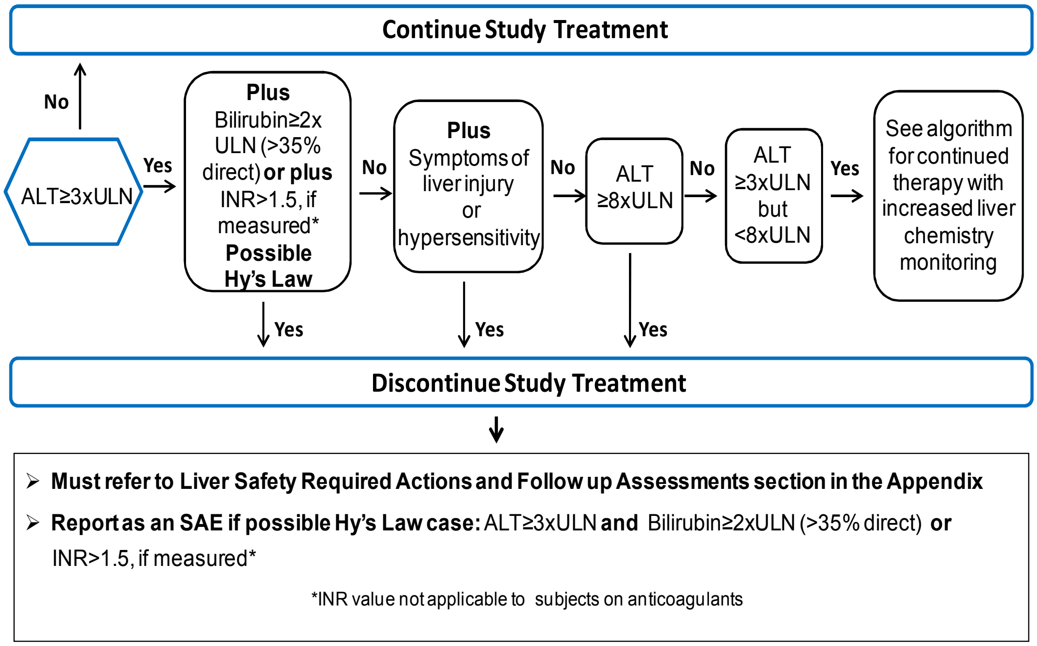

Liver Safety Required Actions and Follow up Assessments Section can be found in Section 6.3.1.1 and Section 6.3.2.1.

11.4.2. Liver Monitoring Event Algorithm with Continued Therapy for ALT≥3xULN but <8xULN

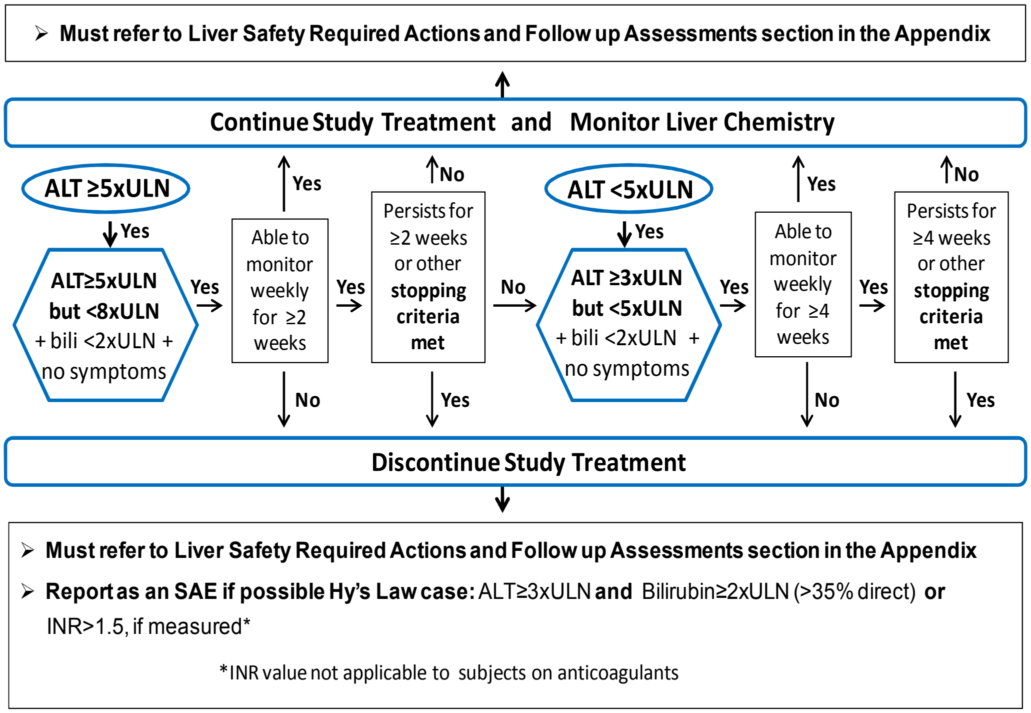

Liver Safety Required Actions and Follow up Assessments Section can be found in Section 6.3.1.1 and Section 6.3.2.1

References

Andrade RJ, Robles M, Lucena MI. Rechallenge in drug-induced liver injury: the attractive hazard. Expert Opin Drug Saf. 2009;8:709-714.

Hunt, CM. Mitochondrial and immunoallergic injury increase risk of positive drug rechallenge after drug-induced liver injury: A systematic review. Hepatol. 2010;52:2216-2222.

Papay JI, Clines D, Rafi R, Yuen N, Britt SD, Walsh JS, Hunt CM. Drug-induced liver injury following positive drug rechallenge. Regul Tox Pharm. 2009;54:84-90.

11.5. Appendix 5: Clinical Laboratory Tests

Hematology

Total white blood cell count
Differential:
- Absolute Neutrophils
  - Segmented Neutrophils
  - Band Neutrophils
  - Myelocytes
  - Metamylocytes
  - Promyelocytes
- Lymphocytes
- Monocytes
- Eosinophils
- Basophils

Hemoglobin
Hematocrit
Red blood cell (RBC) count
Platelet count
Prothrombin time (PT)
Partial thromboplastin time (PTT)
Serum Pregnancy

Urinalysis

Protein
Glucose
Ketones
Occult blood
Microscopic examination including:WBC per hpf, RBC per hpf, Dysmorphic RBC
Casts (specified by type eg, RBC, WBC)
Spot Urine (protein : creatinine ratio)
Urine Pregnancy
Alcohol and Drug Screen

Modified Chem-20

Electrolytes:Sodium
Potassium
Magnesium
Chloride
Carbon dioxide
- Calcium adjusted for Albumin
- Inorganic Phosphate

Enzymes:
- SGOT (AST)
- SGPT (ALT)
- Alkaline Phosphatase
- Gamma glutanyl transpeptidase (GGT)
- Lactic dehydrogenase (LDH)

Other:
- Creatinine
- Blood urea nitrogen (BUN)
- BUN/creatinine ratio
- Bilirubin, total
- Protein, total
- Albumin
- Uric acid
- Glucose
- HIV-1/2 antibody
- Hepatitis C antibody
- Hepatitis B surface antigen
- Estimated Creatinine Clearance/ GFR (Cockroft-Gault)

Biological Markers
BLyS protein
Serum complement (C3 and C4)
C-Reactive Protein (CRP)
B-cell subtypes ($CD20^+$, $CD20^+/27^+$ memory, $CD20^+/27^-$ naïve, $CD20^+/69^+$ activated, $CD20^+/138^+$ plasmacytoid, $CD19^+/27^{BRIGHT}/38^{BRIGHT}$ SLE subset and $CD20^-/138^+$ plasma cells).

Immunoglobulins
Serum immunoglobulin isotypes: IgG, IgM, IgA

PK and Immunogenicity

Autoantibodies
ANA titer and OD
Anti-dsDNA
aCL

Adverse Event and Laboratory Value Severity Grade Tables

HEMATOLOGY	GRADE 1 MILD	GRADE 2 MODERATE	GRADE 3 SEVERE	GRADE 4 POTENTIALLY LIFE-THREATENING
Hemoglobin	> 9.5 - 11.0 g/dL	> 8.0 – 9.5 g/dL	6.5 - 8.0 g/dL	< 6.5 g/dL
Leukocytes	3000-3999/mm3	2000-2999/mm3	1000-1999/mm3	< 1000/mm3
Absolute Neutrophil Count	1500-1999/mm3	1000-1499/mm3	500-999/mm3	< 500/mm3
Platelets	75,000 - 99,999/mm3	50,000 – 74,999/mm3	25,000 - 49,999/mm3	< 25,000/mm3
Prothrombin Time (PT)	> 1.0-1.25 x ULN*	> 1.25-1.5 x ULN	> 1.5-3.0 x ULN	> 3.0 x ULN
Partial Thromboplastin Time (PTT)	> 1.0-1.66 x ULN	> 1.66-2.33 x ULN	> 2.33-3.0 x ULN	> 3.0 x ULN
Methemoglobin	5.0-10.0 %	10.1-15.0 %	15.1-20.0 %	> 20%

(continued)

1. *ULN = Upper Limit of Normal.
2. Modified from DMID Adult Toxicity Tables, 2001

Adverse Event and Laboratory Value Severity Grade Tables (continued)

CARDIOVASCULAR	GRADE 1 MILD	GRADE 2 MODERATE	GRADE 3 SEVERE	GRADE 4 POTENTIALLY LIFE-THREATENING
Cardiac Arrhythmia	-	Asymptomatic/transient; dysrhythmia; no treatment req	Recurrent/persistent dysrhythmia. Symptomatic; treatment req	Unstable dysrhythmia hospitalization and treatment required
Hypotension	Transient orthostatic hypotension, no treatment	Symptoms correctable with oral fluid treatment	IV fluid req, no hospitalization req	Hospitalization req
Hypertension	Transient, increase > 20 mm/Hg; no treatment	Recurrent; chronic increase > 20 mm/Hg, treatment req	Acute treatment req; out subject hospitalization possible	Hospitalization req
Pericarditis	Minimal effusion	Mild/moderate asymptomatic effusion, no treatment	Symptomatic effusion, pain, ECG changes	Tamponade OR pericardiocentesis OR surgery req
Hemorrhage, Blood Loss	-	Mildly symptomatic; no treatment required	Gross blood loss OR 1-2 units transfused	Massive blood loss OR > 2 units transfused

(continued)

1. Modified from DMID Adult Toxicity Tables, 2001

Adverse Event and Laboratory Value Severity Grade Tables (continued)

CHEMISTRIES	GRADE 1 MILD	GRADE 2 MODERATE	GRADE 3 SEVERE	GRADE 4 POTENTIALLY LIFE-THREATENING
Sodium				
Hyponatremia	130-135 meq/L	123-129 meq/L	116-122 meq/L	< 116 meq/L
Hypernatremia	146-150 meq/L	151-157 meq/L	158-165 meq/L	> 165 meq/L
Potassium				
Hypokalemia	3.0-3.4 meq/L	2.5-2.9 meq/L	2.0-2.4 meq/L	< 2.0 meq/L
Hyperkalemia	5.6-6.0 meq/L	6.1-6.5 meq/L	6.6-7.0 meq/L	> 7.0 meq/L
Phosphate				
Hypophosphatemia	2.0-2.4 mg/dL	1.5-1.9 mg/dL	1.0-1.4 mg/dL	< 1.0 mg/dL
Calcium- (Corrected For Albumin)				
Hypocalcemia	7.8-8.4 mg/dL	7.0-7.7 mg/dL	6.1-6.9 mg/dL	< 6.1 mg/dL
Hypercalcemia	10.6-11.5 mg/dL	11.6-12.5 mg/dL	12.6-13.5 mg/dL	>13.5 mg/dL
Magnesium				
Hypomagnesemia	1.2-1.4 meq/L	0.9-1.1 meq/L	0.6-0.8 meq/L	< 0.6 meq/L
Albumin				
Hypoalbuminemia	3.00-3.49 g/dL	2.50-2.99 g/dL	2.00-2.49 g/dL	< 2.00 g/dL
Bilirubin (Total)				
Hyperbilirubinemia (Total)	> 1.0-1.5 x ULN	> 1.5-2.5 x ULN	> 2.5-5 x ULN	> 5 x ULN
Glucose				
Hypoglycemia	55-64 mg/dL	40-54 mg/dL	30-39 mg/dL	< 30 mg/dL
Hyperglycemia (nonfasting & no prior diabetes)	116-160 mg/dL	161-250 mg/dL	251-500 mg/dL	> 500 mg/dL
Triglycerides	151-399 mg/dL	400-750 mg/dL	751-1200 mg/dL	> 1200 mg/dL
Creatinine	> 1.0-1.5 x ULN	> 1.5-3.0 x ULN	> 3.0-6.0 x ULN	> 6.0 x ULN

1. Modified from DMID Adult Toxicity Tables, 2001

Adverse Event and Laboratory Value Severity Grade Tables (continued)

CHEMISTRIES (continued)	GRADE 1 MILD	GRADE 2 MODERATE	GRADE 3 SEVERE	GRADE 4 POTENTIALLY LIFE-THREATENING
Uric Acid				
Hyperuricemia	7.5-10.0 mg/dL	10.1-12.0 mg/dL	12.1-15.0 mg/dL	> 15.0 mg/dL
Liver Transferases (AST, ALT, and GGT)	1.25-2.5 x ULN	> 2.5-5.0 x ULN	> 5.0-10.0 x ULN	> 10.0 x ULN
Alkaline Phosphatase	1.25-2.5 x ULN	> 2.5-5.0 x ULN	> 5.0-10.0 x ULN	> 10.0 x ULN
Pancreatic Enzymes				
Amylase	> 1.0-1.5 x ULN	> 1.5-2.0 x ULN	> 2.0-5.0 x ULN	> 5.0 x ULN
Pancreatic amylase	> 1.0-1.5 x ULN	> 1.5-2.0 x ULN	> 2.0-5.0 x ULN	> 5.0 x ULN
Lipase	> 1.0-1.5 x ULN	> 1.5-2.0 x ULN	> 2.0-5.0 x ULN	> 5.0 x ULN
*Hypoglobulinemia (IgG)**	550-700 mg/dL	400-549 mg/dL	250-399 mg/dL	< 250 mg/dL
				(continued)

1. *[Eibl, 1995; Goldfarb, 2001; Yamini, 2001].
2. Modified from DMID Adult Toxicity Tables, 2001

Adverse Event and Laboratory Value Severity Grade Tables (continued)

GASTROINTESTINAL	GRADE 1<br>MILD	GRADE 2<br>MODERATE	GRADE 3<br>SEVERE	GRADE 4<br>POTENTIALLY LIFE-THREATENING
Nausea	Mild OR transient; reasonable intake maintained	Mod discomfort OR intake decreased for < 3 days	Severe discomfort OR minimal intake for ≥ 3 days	Hospitalization required
Vomiting	Mild OR transient; 2-3 episodes/day OR mild vomiting lasting < 1 week	Mod OR persistent; 4-5 episodes per day; OR vomiting lasting ≥ 1 week	Severe vomiting of all foods/fluids in 24 hours OR orthostatic hypotension OR IV treatment req	Hypotensive shock OR hospitalization required for IV treatment req
Diarrhea	Mild or transient; 3-4 loose stools per day OR mild diarrhea lasting < 1 week	Mod OR persistent; 5-7 loose stools per day or diarrhea lasting ≥ 1 week	Bloody diarrhea; OR orthostatic hypotension OR > 7 loose stools/day OR IV treatment req	Hypotensive shock OR hospitalization req
Oral Discomfort/Dysphagia	Mild discomfort, no difficulty swallowing	Difficulty swallowing but able to eat and drink	Unable to swallow solids	Unable to drink fluids; IV fluids req
Constipation	Mild	Moderate	Severe	Distention with vomiting

Modified from DMID Adult Toxicity Tables, 2001

Adverse Event and Laboratory Value Severity Grade Tables (continued)

RESPIRATORY	GRADE 1 MILD	GRADE 2 MODERATE	GRADE 3 SEVERE	GRADE 4 POTENTIALLY LIFE-THREATENING
Cough (for aerosol studies)	Transient; no treatment	Treatment associated cough; inhaled bronchodilator	Uncontrolled cough; systemic treatment req	
Bronchospasm Acute	Transient; no treatment; FEV1 70% to < 80% (or peak flow)	treatment req; normalizes with bronchodilator; FEV1 50% to < 70% (or peak flow)	No Normalization with bronchodilator; FEV 25% to < 50% (or peak flow), retractions	Cyanosis; FEV1 < 25% (or peak flow) OR intubated
Dyspnea	Dyspnea on exertion	Dyspnea with normal activity	Dyspnea at rest	Dyspnea requiring O2 therapy

URINALYSIS	GRADE 1 MILD	GRADE 2 MODERATE	GRADE 3 SEVERE	GRADE 4 POTENTIALLY LIFE-THREATENING
Proteinuria				
Dispstick Protein	1 +	2-3 +	4 +	Nephrotic syndrome
Spot Urine: Protein:Creatinine Ratio mg/mg	0.2-1.0	> 1.0-2.0	> 2.0-3.5	> 3.5
24 Hour Urine: Protein	200 mg - 1g loss/day	> 1-2 g loss/day	> 2-3.5 g loss/day	Nephrotic syndrome OR > 3.5 g loss/day
Hematuria	Microscopic only > 3 to < 10 RBC/hpf	Gross, No clots ≥ 10 RBC/hpf	Gross plus clots OR RBC casts	Obstructive OR transfusion required

RBC = red blood cell; hpf = high power field.
Modified from DMID Adult Toxicity Tables, 2001

Adverse Event and Laboratory Value Severity Grade Tables (continued)

MISCELLANEOUS	GRADE 1 MILD	GRADE 2 MODERATE	GRADE 3 SEVERE	GRADE 4 POTENTIALLY LIFE-THREATENING
Fever (oral > 12 hours)	37.7-38.5°C or 100.0-101.5°F	38.6-39.5°C OR 101.6-102.9°F	39.6-40.5°C OR 103-105°F	> 40.5°C OR > 105°F
Headache	Mild; No treatment req	Mod; or non-narcotic analgesia treatment	Severe; OR responds to initial narcotic treatment	Intractable; OR requiring repeated narcotic treatment
Allergic Reaction	Pruritus without rash	Localized urticaria	Generalized urticaria angioedema	Anaphylaxis
Cutaneous/Rash/ Dermatitis	Erythema, pruritus rash OR dry desquamation	Diffuse maculopapular OR dry desquamation	Vesiculation OR moist desquamation ulceration	ANY ONE: mucous membrane involvement, suspected Stevens-Johnson (TEN), erythema multiforme, necrosis req surgery, exfoliative dermatitis
Local Reaction (secondary to parenteral treatment- not vaccination or skin test)	Erythema	Induration < 10 mm OR inflammation OR phlebitis	Induration > 10 mm OR ulceration	Necrosis of skin
Fatigue	Normal activity Reduced < 25%	Normal activity Reduced 25-50%	Normal activity reduced > 50%; cannot work	Unable to care for self

1. Modified from DMID Adult Toxicity Tables, 2001

Brunner HI, *et al. Ann Rheum Dis* 2020; 79:1340–1348. doi: 10.1136/annrheumdis-2020-217101

Adverse Event and Laboratory Value Severity Grade Tables (continued)

NEUROLOGIC	GRADE 1<br>MILD	GRADE 2<br>MODERATE	GRADE 3<br>SEVERE	GRADE 4<br>POTENTIALLY LIFE-THREATENING
Neuro-cerebellar	Slight incoordination OR dysdiadochokinesia	Intention tremor OR dysmetria OR slurred speech OR nystagmus	Ataxia requiring assistance to walk or arm incoordination interfering with ADLs	Unable to stand
Neuro-psych/ mood		none	Severe mood changes requires medical intervention	Acute psychosis requiring Hospitalization
Paresthesia (burning, tingling, etc)	Mild discomfort; no treatment needed	Mod discomfort non-narcotic analgesia req	Severe discomfort; OR narcotic analgesia req with symptomatic improvement	Incapacitating; OR not responsive to narcotic analgesia
Neuro-motor	Mild weakness in muscle of feet but able to walk and/or mild increase or decrease in reflexes	Mod weakness in feet (unable to walk on heels and/or toes), mild weakness in hands, still able to do most hand tasks and/or loss of previously present reflex or development of hyperreflexia and/or unable to do deep knee bends due to weakness	Marked distal weakness (unable to dorsiflex toes or foot drop), and mod proximal weakness ie, in hands interfering with ADLs and/or requiring assistance to walk and/or unable to rise from chair unassisted	Confined to bed or wheelchair because of muscle weakness
Neuro-sensory	Mild impairment sensations, (ie, vibratory, pinprick, hot/cold in great toes) in focal area or symmetrical distribution	Mod impairment mod de-sensation, (ie, of vibratory, pinprick, hot/cold to ankles) and/or joint position or mild impairment that is not symmetrical.	Severe impairment (dec or loss of sensation to knees or wrists) or loss of sensation of at least mod degree in multiple different body areas (ie, upper and lower extremities)	Sensory loss involves limbs and trunk

Brunner HI, *et al. Ann Rheum Dis* 2020; 79:1340–1348. doi: 10.1136/annrheumdis-2020-217101

11.6. Appendix 6: SELENA SLEDAI Disease Assessment Scales

CCI - This section contained Clinical Outcome Assessment data collection questionnaires or indices, which are protected by third party copyright laws and therefore have been excluded.

[redacted]

11.7. Appendix 7: SLE Flare Index

CCI - This section contained Clinical Outcome Assessment data collection questionnaires or indices, which are protected by third party copyright laws and therefore have been excluded.

[redacted]

11.8. Appendix 8: Physician's Global Disease Assessment

CCI - This section contained Clinical Outcome Assessment data collection questionnaires or indices, which are protected by third party copyright laws and therefore have been excluded.

[redacted]

11.9. Appendix 9: Parent's Global Disease Assessment

Considering all the ways the illness affects your child, please evaluate how he/she feels <u>at the moment</u>

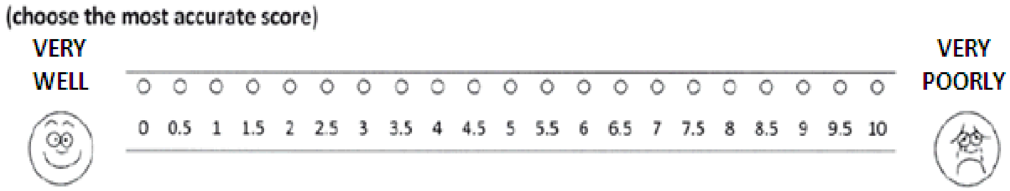

11.10. Appendix 10: BILAG Index Assessment

CCI - This section contained Clinical Outcome Assessment data collection questionnaires or indices, which are protected by third party copyright laws and therefore have been excluded.

[redacted]

11.11. Appendix 11: Pediatric SLICC/ACR Damage Index

CCI - This section contained Clinical Outcome Assessment data collection questionnaires or indices, which are protected by third party copyright laws and therefore have been excluded.

[redacted]

11.12. Appendix 12: Columbia Suicide Severity Rating Scale C-SSRS

CCI - This section contained Clinical Outcome Assessment data collection questionnaires or indices, which are protected by third party copyright laws and therefore have been excluded.

[redacted]

Brunner HI, *et al*. *Ann Rheum Dis* 2020; 79:1340–1348. doi: 10.1136/annrheumdis-2020-217101

Since Last Visit

Columbia Suicide-Severity Rating Scale (C-SSRS) Since Last Visit

CCI - This section contained Clinical Outcome Assessment data collection questionnaires or indices, which are protected by third party copyright laws and therefore have been excluded.

[redacted]

11.13. Appendix 13: Possible Suicidality-Related Questionnaire

CCI - This section contained Clinical Outcome Assessment data collection questionnaires or indices, which are protected by third party copyright laws and therefore have been excluded.

[redacted]

11.14. Appendix 14: Pediatric Quality of Life Inventory - Generic Core Scale (PedsQL)

CCI - This section contained Clinical Outcome Assessment data collection questionnaires or indices, which are protected by third party copyright laws and therefore have been excluded.

[redacted]

11.15. Appendix 15: Pediatric Quality of Life Multidimensional Fatigue Scale (PedsQL Fatigue)

CCI - This section contained Clinical Outcome Assessment data collection questionnaires or indices, which are protected by third party copyright laws and therefore have been excluded.

[redacted]

CONFIDENTIAL 2018N394147_00

The GlaxoSmithKline group of companies

Division: Worldwide Development
Retention Category: GRS019
Information Type: Clinical Statistics Report

**Title:**	Bayesian Extrapolation Analyses of Belimumab Efficacy in Paediatric SLE Subjects.

Compound Number: GSK1550188

Effective Date: 15-JAN-2019

Description: This report describes a Bayesian analysis conducted in support of the extrapolation of the efficacy of belimumab in adults (≥18 years old) to paediatric subjects (≥5-17 years old) with SLE using data from studies BEL114055, HGS1006-C1056 and HGS10076-C1057.

Subject: BEL114055, GSK1550188, belimumab, paediatrics, efficacy, extrapolation, Bayesian inference

Authors: PPD (Biostatistics, GlaxoSmithKline)

Approved by:

PPD<br>Director, Biostatistics	15-JAN-2019

Copyright 2019 the GlaxoSmithKline group of companies. All rights reserved.
Unauthorized copying or use of this information is prohibited.

CONFIDENTIAL 2018N394147_00

TABLE OF CONTENTS

PAGE

ABBREVIATIONS 3

1. INTRODUCTION 4

2. OBJECTIVES 4
   2.1. Objectives 4
   2.2. Endpoints 4

3. STUDIES INCLUDED 4

4. METHODS 4
   4.1. Analysis Inputs 5
      4.1.1. SRI 5
      4.1.2. SELENA SLEDAI 6
   4.2. Robust Mixture Prior Bayesian Analysis 7

5. RESULTS 8
   5.1. SRI 8
   5.2. SELENA SLEDAI 10

6. DISCUSSION 13

7. REFERENCES 14

ABBREVIATIONS

CrI	Credible Interval
GSK	GlaxoSmithKline
IV	Intravenous
kg	kilogram
mg	milligram
OR	Odds Ratio
RMP	Robust Mixture Prior
SE	Standard Error

Trademark Information

Trademarks of the GlaxoSmithKline group of companies
BENLYSTA

Trademarks not owned by the GlaxoSmithKline group of companies
None

1. INTRODUCTION

The intravenous (IV) formulation of belimumab (BENLYSTA) is currently approved for the treatment of adult patients with active, autoantibody-positive systemic lupus erythematosus (SLE) who are receiving standard therapy. The original marketing approvals of belimumab were supported by two pivotal Phase 3 studies (C1056 and C1057) in adult subjects.

To aid in interpretation of the results of study BEL114055 in paediatric SLE patients, the previous adult studies (C1056 and C1057) may provide some useful relevant information.

The aim of this analysis is to further evaluate the efficacy of belimumab in paediatric SLE patients by utilising relevant information from the adult patients.

A Bayesian statistical approach can be used to explicitly borrow information from the adult studies to provide inferences on the paediatric population [Gamalo-Siebers, 2017].

2. OBJECTIVES

2.1. Objectives

The objective of this analysis is to further support the effect of BENLYSTA on paediatric SLE patients.

2.2. Endpoints

Two key endpoints have been assessed.

1. SRI (SLE Response Index) response rate at Week 52 [Furie, 2009].
    a. The primary endpoint in all studies.
2. Change from baseline in SELENA SLEDAI score at Week 52 [Petri, 2005].

3. STUDIES INCLUDED

Paediatric study (BEL114055) and the Phase 3 pivotal IV adult studies (C1056 and C1057) were included. Only the 10 mg/kg approved adult dose was used in this anlaysis.

4. METHODS

A Bayesian approach was used to draw inference about the treatment effect in the paediatric population by supplementing the paediatric data with data from the adult population. For the SRI response, a binary outcome, the treatment effect of interest is taken to be the log-odds ratio of response in Benlysta to Placebo. For the SELENA SLEDAI score, the treatment effect is the difference in means in the change from baseline between Benlysta and Placebo. Separate analyses were carried out for each outcome.

A robust mixture prior (RMP) [Schmidli, 2014] was used for the treatment effect in paediatrics δ_p. A mixture of Normals with two components was used: an informative one based on the efficacy results from adults and a vague one to enable the prior to downweight the information from adults in case of conflict between the observed results in the two populations. That is, the prior for δ_p has the form

$$p(\delta_p) = w \cdot p_A(\delta_p) + (1 - w) \cdot p_V(\delta_p) \quad (1)$$

where $p_A(\delta_p)$ is the component containing the information from adults and $p_V(\delta_p)$ is the vague component with variance scaled to represent information equivalent to one subject per am.

The prior weight, w, assigned to the adult prior component represents the prior degree of belief in the similarity of the paediatric and adult treatment effects. At lower prior weights the mixture prior presents a heavier tailed distribution with more prior weight being applied to the vague prior component. When the mixture prior is combined with the observed paediatric efficacy data, w is updated according to how consistent the paediatric data are with the adult prior: the stronger the evidence of consistency, the greater the increase in the posterior weight (w∗) relative to the prior weight (w). Conversely, when there is prior-data conflict, w∗ will be lower than w and will tend to zero as evidence of conflict increases, so that the adult information is down-weighted and posterior inference is based almost entirely on the paediatric data. For details on the approach see Section 4.2.

The above analysis was carried out for weights $w = 0, 0.05, 0.1, 0.15, \ldots, 1$ covering the full range of possible values from $w = 0$, representing no borrowing at all from adults, to $w = 1$, implying a pooled analysis of adults and paediatrics data. In the spirit of a tipping point analysis, for each weight, how the posterior distribution of the treatment effect δ_p supports the superiority of Benlysta versus Placebo in paediatrics was assessed by:

i. Calculating the 95% credible interval for the estimated paediatric treatment effect
ii. Calculating an indicator of whether the posterior probability of superiority of Benlysta over placebo in paediatrics exceeded 97.5%, i.e. $\Pr(\delta_p > 0 \mid data) > 0.975$.

These analyses are to inform of the smallest prior weight w, i.e. the minimum prior belief in the similarity of adult and paediatric treatment effects, leading to robust evidence supporting superiority of Benlysta over Placebo in paediatrics.

The analyses were implemented using R v3.5.0 and the RBesT package v 1.3-3 [Weber, 2018].

4.1. Analysis Inputs

4.1.1. SRI

The inputs for the present analysis are the estimated log-odds ratio of response in Benlysta to Placebo in adults and paediatrics as well the total sample size in the paediatric study.

The above quantities were estimated from the number of treated subjects n_A and responders r_A per arm of the pooled analysis of the two studies in adults and their counterparts n_p and r_p in the paediatric study. With these results the corresponding proportion of responders in the two populations $\hat{p}_A$ and $\hat{p}_p$ for each treatment arm were estimated (see Table 1).

Table 1 SRI analyses: number of responders r, number of subjects treated n and estimated proportion of SRI response $\hat{p}$ in the two studies in adults combined and in the paediatrics study

Treatment	Adults	Paediatrics
	r_A/n_A $(\hat{p}_A)$	r_p/n_p $(\hat{p}_p)$
Placebo	218/562 (0.388)	17/39 (0.436)
Benlysta (10 mg/kg)	285/563 (0.506)	28/53 (0.528)

Based on the results observed (Table 1), the corresponding estimated odds ratios (OR) of response in Benlysta to Placebo are:

- Adults: $OR_A = \frac{\frac{0.506}{1-0.506}}{\frac{0.388}{1-0.388}} = 1.616$
- Paediatrics: $OR_p = \frac{\frac{0.528}{1-0.528}}{\frac{0.436}{1-0.436}} = 1.447$

For this analysis a normal approximation for the log(OR) was used. The observed log-odds in adults and paediatrics is denoted by y_A and y_p, respectively, and will be taken as the observed data in this analysis. Analogously, the associated standard errors will be denoted by s_A and s_p. The values of those four quantities are:

- Adults:
  - $y_A = \log(OR_A) = \log(1.616) = 0.48$
  - $s_A = \sqrt{\left(\frac{1}{218} + \frac{1}{(562-218)} + \frac{1}{285} + \frac{1}{563-285}\right)} = 0.121$
- Paediatrics:
  - $y_p = \log(OR_p) = \log(1.447) = 0.369$
  - $s_p = \sqrt{\frac{1}{17} + \frac{1}{39-17} + \frac{1}{28} + \frac{1}{53-28}} = 0.424$

4.1.2. SELENA SLEDAI

The inputs for this analysis are the difference between Benlysta and Placebo in the mean change from baseline in the SELENA SLEDAI score and associated standard errors.

Table 2 shows the observed results for the change from baseline in SELENA SLEDAI score in each treatment arm in the two combined studies in adults and in the paediatrics study.

Brunner HI, *et al. Ann Rheum Dis* 2020; 79:1340–1348. doi: 10.1136/annrheumdis-2020-217101

Table 2 SELENA SLEDAI analyses: mean change from baseline and SE in SELENA SLEDAI score for each treatment arm in the two studies in adults combined (with n_A subjects treated) and in the paediatrics study (with n_P subjects treated)

**Treatment**	**Adults**	**Paediatrics**
	Mean (SE)<br>n_A	Mean (SE)<br>n_P
Placebo	-3.18 (0.18)<br>562	-4.1 (0.71)<br>39
Benlysta (10 mg/kg)	-4.36 (0.19)<br>563	-4.9 (0.62)<br>53

As before, let y_A and y_P be the difference between treatments (Benlysta - Placebo) in the change from baseline in the SELENA SLEDAI score and let s_A and s_P their associated standard errors. These four quantities are:

- Adults:
  - $y_A = -4.36 + 3.18 = -1.18$
  - $s_A = \sqrt{0.18^2 + 0.19^2} = 0.262$
- Paediatrics:
  - $y_P = -4.9 + 4.1 = -0.8$
  - $s_P = \sqrt{0.71^2 + 0.62^2} = 0.943$

4.2. Robust Mixture Prior Bayesian Analysis

To infer about the paediatric treatment effect δ_P the following Bayesian model was used:

$$y_P \sim N(\delta_P, s_P^2)$$
$$\delta_P \sim w \cdot N(y_A, s_A^2) + (1-w) \cdot N(0, m \cdot s_P^2)$$

Where y_P is the estimated treatment effect in the paediatric study and s_P is the corresponding SE, which is assumed to be known.

The prior distribution for δ_P is a mixture of the form in expression (1), with $p_A(\delta_P) = N(y_A, s_A^2)$, where y_A, s_A are the estimated treatment effect and SE, respectively, from the combined adult studies, and the vague component is $p_V(\delta_P) = N(0, m \cdot s_P^2)$; w is the prior weight to be given to the information from adults.

It was conservatively assumed that the mean of the vague component is 0 (i.e. centred at the null hypothesis of no effect). The variance is assumed to be $m \cdot s_P^2$, where m is chosen such that the effective sample size of this component is worth just 1 paediatric subject per arm. Assuming $s_P^2 = SD^2/N_P$, where SD is the standard deviation and N_P is the total sample size in the paediatric study, then the variance of the vague component is set to be $m \cdot s_P^2 = SD^2/2$, which leads to

$$m \cdot s_p^2 = \frac{SD^2}{2} \rightarrow m\frac{SD^2}{N_p} = \frac{SD^2}{2} \rightarrow m = \frac{N_p}{2}$$

Since the total sample size in the paediatric study is $N_p = 39 + 53$, it follows that $m = (39 + 53)/2 = 46$.

Based on the above consideration, the specific priors for the treatment effect δ_p used for each endpoint are as follows:

- SRI: $\delta_p \sim w \cdot N(0.48, 0.015) + (1 - w) \cdot N(0, 8.27)$
- SELENA SLEDAI: $\delta_p \sim w \cdot N(-1.18, 0.00069) + (1 - w) \cdot N(0, 40.91)$

The mean and variance of the two components of the mixture and the prior weight are updated with the observed data y_p from the paediatric study as described in Schmidli et al [Schmidli, 2014].

To find the minimum weight w for which superiority is shown in the paediatric population, these steps were followed for each of 21 different values of the prior weight w ranging between 0 and 1 in steps of 0.05:

1. Build the mixture prior with two components described above.
2. Update that prior parameters, including the prior weight, with results from the paediatric study.
3. Compute posterior summaries for δ_p: mean, median and the equal-tail posterior 95% credibility interval.

Note that if the 95% credible interval (CrI) for δ_p excludes 0, then the posterior probability of showing superiority of Benlysta over Placebo is at least 97.5%.

5. RESULTS

5.1. SRI

Table 3 shows numerical summaries of the posterior distribution of the log odds ratio δ_p obtained for each prior weight w considered. Specifically, the posterior mean and median as well as the equal-tail 95% CrIs is shown. To aid visualisation, Figure 1 shows a point range graph with posterior means (points) and 95% CrIs. Values of the prior weight w of 0.55 or larger lead to posterior distributions with 95% CrIs lying to the right of 0, i.e. with more than 97.5% probability mass in the range of treatment effect values favouring Benlysta over Placebo (Table 3, Figure 1).

Brunner HI, *et al. Ann Rheum Dis* 2020; 79:1340–1348. doi: 10.1136/annrheumdis-2020-217101

Table 3 Posterior mean, median and 95% credibility interval for the log odds ratio of SRI response in Benlysta to Placebo for several prior weights *w* ranging from 0 to 1 in steps of 0.05

w	Mean	Median	95% CrI
0.00	0.36	0.36	(-0.46, 1.18)
0.05	0.39	0.42	(-0.41, 1.13)
0.10	0.41	0.44	(-0.36, 1.08)
0.15	0.42	0.45	(-0.32, 1.04)
0.20	0.43	0.46	(-0.27, 1.00)
0.25	0.44	0.46	(-0.23, 0.95)
0.30	0.44	0.46	(-0.19, 0.91)
0.35	0.45	0.46	(-0.15, 0.87)
0.40	0.45	0.46	(-0.11, 0.84)
0.45	0.45	0.47	(-0.06, 0.80)
0.50	0.46	0.47	(-0.01, 0.78)
0.55	0.46	0.47	(0.04, 0.76)
0.60	0.46	0.47	(0.09, 0.75)
0.65	0.46	0.47	(0.14, 0.74)
0.70	0.46	0.47	(0.17, 0.73)
0.75	0.47	0.47	(0.19, 0.72)
0.80	0.47	0.47	(0.21, 0.72)
0.85	0.47	0.47	(0.22, 0.71)
0.90	0.47	0.47	(0.23, 0.71)
0.95	0.47	0.47	(0.24, 0.70)
1.00	0.47	0.47	(0.24, 0.70)

Brunner HI, *et al*. *Ann Rheum Dis* 2020; 79:1340–1348. doi: 10.1136/annrheumdis-2020-217101

Figure 1 Posterior mean (points) and 95% credibility intervals (lines) of the odds ratio of SRI response in Benlysta to Placebo for several prior weights w ranging from 0 to 1 in steps of 0.05

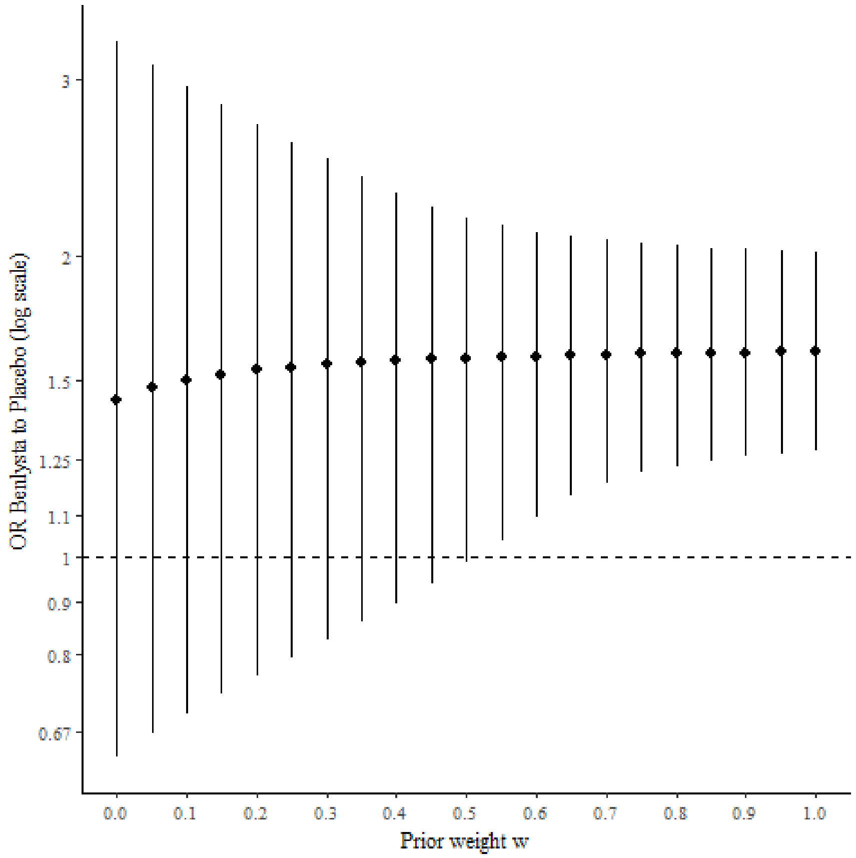

5.2. SELENA SLEDAI

Table 4 shows numerical summaries of the posterior distribution of the treatment difference δ_p obtained for each prior weight w considered. Specifically, the posterior mean and median as well as the equal-tail 95% CrI is shown. These results are also displayed graphically (Figure 2). Values of the prior weight of 0.55 or larger lead to posterior distributions with 95% CrIs lying to the left of 0, i.e. with more than 97.5% probability mass in the range of treatment effect values favouring Benlysta over Placebo (Table 4, Figure 2).

Table 4 Posterior mean, median and 95% credibility interval for the difference in the SELENA SLEDAI change from baseline between Benlysta and Placebo for several prior weights *w* ranging from 0 to 1 in steps of 0.05

w	Mean	Median	95% CrI
0.00	-0.78	-0.78	(-2.61, 1.05)
0.05	-0.87	-0.98	(-2.50, 0.93)
0.10	-0.93	-1.05	(-2.39, 0.83)
0.15	-0.98	-1.08	(-2.30, 0.73)
0.20	-1.01	-1.1	(-2.21, 0.64)
0.25	-1.03	-1.11	(-2.12, 0.55)
0.30	-1.05	-1.12	(-2.03, 0.46)
0.35	-1.07	-1.13	(-1.95, 0.37)
0.40	-1.08	-1.13	(-1.88, 0.28)
0.45	-1.09	-1.13	(-1.83, 0.18)
0.50	-1.1	-1.14	(-1.78, 0.07)
0.55	-1.11	-1.14	(-1.75, -0.04)
0.60	-1.12	-1.14	(-1.73, -0.17)
0.65	-1.12	-1.14	(-1.71, -0.31)
0.70	-1.13	-1.15	(-1.70, -0.43)
0.75	-1.13	-1.15	(-1.69, -0.51)
0.80	-1.14	-1.15	(-1.68, -0.56)
0.85	-1.14	-1.15	(-1.67, -0.60)
0.90	-1.15	-1.15	(-1.66, -0.62)
0.95	-1.15	-1.15	(-1.65, -0.64)
1.00	-1.15	-1.15	(-1.65, -0.66)

Figure 2 Posterior mean (points) and 95% credibility intervals (lines) for the difference in the SLEDAI change from baseline between Benlysta and Placebo for several prior weights ranging from 0 to 1 in steps of 0.05

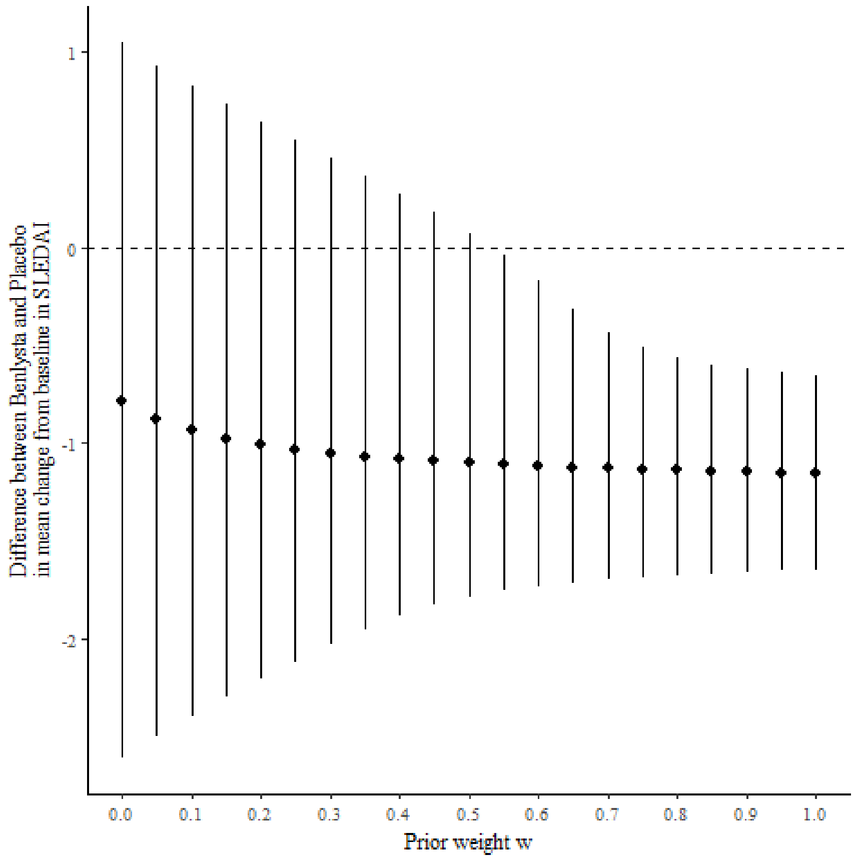

Brunner HI, *et al. Ann Rheum Dis* 2020; 79:1340–1348. doi: 10.1136/annrheumdis-2020-217101

6. DISCUSSION

Given the rarity of SLE in children, the ability to show statistically significant results in paediatrics alone is limited.

The presented robust mixture prior analyses combine an informative prior based on the adult data with a vague prior centred on no treatment effect with a large uncertainty interval.

These analyses therefore consider a range of possible beliefs regarding the acceptability of extrapolation of adult data to paediatric data. The choice of prior beliefs (with prior weights on the adult data ranging from 0, representing no borrowing, to 1 representing pooling information for both populations) could be selected based on the degree of confidence in the relevance of the adult data when estimating effect in paediatrics.

The results for both endpoints were very consistent and both suggest that if it is reasonable to assume a priori at least 55% probability that efficacy in paediatrics is similar to that in adults, then the Bayesian mixture analysis demonstrates superiority of Benlysta over placebo in paediatrics with at least 97.5% probability.

7. REFERENCES

Furie RA, Petri MA, Wallace DJ, et al. Novel evidence-based systemic lupus erythematosus responder index. *Arthritis Rheum.* 2009;61:1143-51.

Gamalo-Siebers M, Savic J, Basu C, Zhao X, Gopalakrishnan M, Gao A, Song G, et al. Statistical modeling for Bayesian extrapolation of adult clinical trial information in pediatric drug evaluation. *Pharm Stats.* 2017;4:232-49.

Petri M, Kim MY, Kalunian KC, et al. Combined oral contraceptives in women with systemic lupus erythematosus. *N Engl J Med* 2005;353:2550-8.

Schmidli H, Gsteiger S, Roychoudhury S, O'Hagan A, Spiegelhalter D, Neuenschwander B. Robust meta-analytic-predictive priors in clinical trials with historical control information. *Biometrics* 2014;70:1023-32.

Weber S. 2018. RBesT: R Bayesian Evidence Synthesis Tools. R package version 1.3-3. https://CRAN.R-project.org/package=RBesT.
